# A Mechanism to Transform Complex Salicinoids with Caffeoylquinic Acids in Lepidopteran Specialist Herbivores (Notodontidae)

**DOI:** 10.1007/s10886-023-01464-9

**Published:** 2023-11-30

**Authors:** Florian Schnurrer, Yoko Nakamura, Christian Paetz

**Affiliations:** Department NMR/Biosynthesis, Max Planck Institute for Chemical Ecology, Hans-Knöll- Straße 8, Jena, 07745 Germany

**Keywords:** *Cerura vinula*, Salicortinoids, Salicaceae, Detoxification, Caffeoylquinic acids, Chlorogenic acid, Stable isotope labeling

## Abstract

Larvae of the Salicaceae-adapted Notodontidae have developed a unique mechanism to metabolize the chemical defenses of their Salicaceae host plants. Salicinoids and salicortinoids are enzymatically transformed into salicyloyl, benzoyl and mixed salicyloyl-benzoyl quinates. The source of quinates and benzoates was previously unknown. To elucidate the origin of quinate and benzoate in the metabolic end-products, we fed *Cerura vinula* caterpillars with ^13^C-labelled poplar defense compounds. Caffeoylquinic acids (CQAs), such as chlorogenic acid, neochlorogenic acid and their methyl esters, were identified as the source of quinates in the caterpillar’s metabolism. Benzoyl substituents in the quinate end-products were found to originate from compounds such as tremulacin or trichocarpin. Salicaceae-adapted Notodontidae caterpillars have the ability to overcome their host plant’s chemical defense by metabolizing CQAs and salicinoids, both abundant defense compounds in Salicacea plants, by a strategy of transformation and recombination. We believe that our study opens up avenues for understanding salicortinoid biotransformation at the enzymatic level.

**Supplementary Information:**

The online version contains supplementary material available at 10.1007/s10886-023-01464-9.

## Introduction

**Fig. 1:**
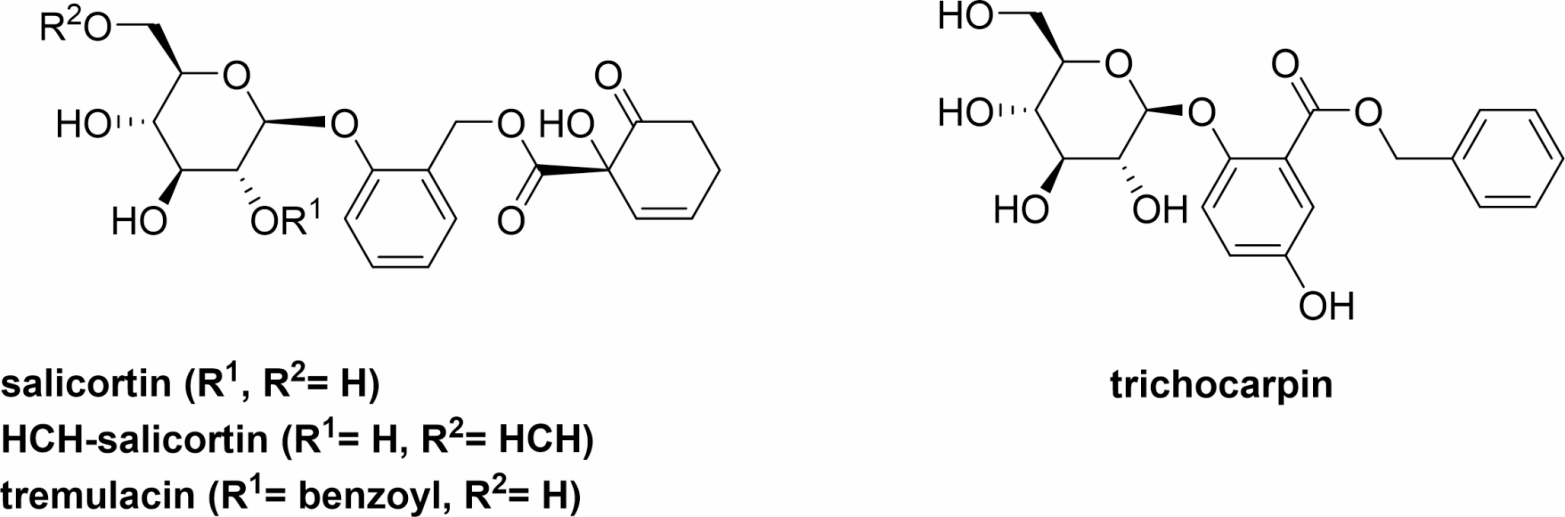
The main defensive compounds in *Populus sp.* salicortinoids (salicortin, HCH-salicortin and tremulacin) and the salicylate trichocarpin

Plants of the genus *Populus* are chemically defended against insect herbivores (Boeckler et al. 2011). The structurally complex salicinoids possessing a 1-hydroxy-6-oxocyclo-hex-2-ene-1-carboxylate (HCH) moiety (salicortinoids) belong to the most abundant defense compounds in *Populus* (Fig. 1) (Thieme and Benecke 1971).

Because salicortin can be found in all poplar species (Boeckler et al. 2011), studies of its metabolization and detrimental effects on lepidopteran generalist herbivores have been conducted over the past few decades with *Papilio glaucus* (Lindroth 1989), *Choristoneura conflictica* (Clausen et al. 1989), *Malacosoma disstria* (Hemming and Lindroth 2000), *Operophtera brumata* (Ruuhola et al. 2001).

It has been shown that salicortin degradation in the generalist *Lymantria dispar* leads to the formation of catechol, which can be oxidized to *ortho*-quinone, a harmful reactive compound. To prevent this, the insects conjugated catechol with glucose or *N*-acetyl-cysteine (Boeckler et al. 2016). The metabolism of salicortin in the lepidopteran Salicaceae specialist *Cerura vinula* (Notodontidae) has recently been investigated. Larvae excreted salicyloyl quinates with their frass after feeding on *Populus nigra* leaves supplemented with [U-^13^C]salicortin. Because no catechol derivatives were detected in the frass, it was concluded that salicortin was deglucosylated and metabolized into two equivalents of salicylate, which were subsequently conjugated with quinic acid (Feistel et al. 2018). As these findings were in contrast to the metabolism described for generalists, a detailed study was conducted involving several Salicaceae specialist herbivores. *Cerura vinula*, *Cerura erminea*, *Clostera anachoreta*, *Furcula furcula*, *Notodonta ziczac* and *Pheosia tremula* (all Notodontidae) were shown to use the same reductive transformations to modify salicortinoids, producing quinate conjugates as end metabolites (Schnurrer and Paetz 2023).

**Fig. 2:**
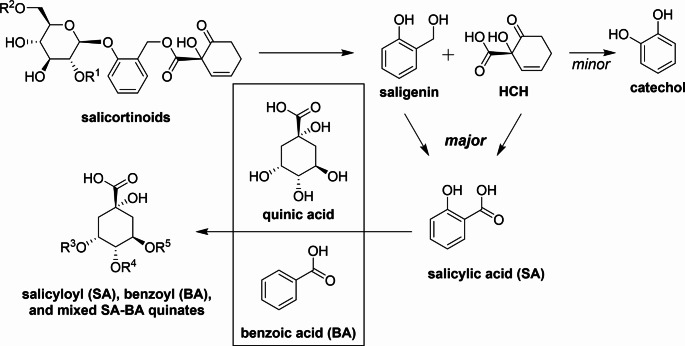
Metabolism of salicortin and salicortinoids in *C. vinula*. After salicortinoid degradation, the metabolites saligenin and HCH are converted to salicylic acid (SA). Quinic acids conjugated with salicylic and/or benzoic acid form the metabolites found in the frass. The sources of quinic acid and benzoic acid (BA) remain to be elucidated

Although several mechanistic details regarding salicortinoid metabolism in Notodontidae have been described two questions remain (Fig. 2): What is the source of the quinates that form the final conjugates of salicortinoid metabolism? What is the source of the benzoates, which occur in conjugation with quinic acids? Answering these questions will complete our understanding of how the specialist lepidopteran herbivores of the Salicaceae have adapted to their host plants. Possible candidates for quinates include caffeoylquinic acids (CQAs), which are chemical defense compounds that also occur in the Salicaceae Poblocka-Olech et al. (2021). Their involvement in the metabolism of another specialist herbivore, *Manduca sexta*, has been recently reported (Heiling et al. 2022). Benzoates are part of the salicortinoid tremulacin and can also be formed from the modified salicylate trichocarpin; both compounds are abundant in trembling aspen (Fig. 1) (Thieme and Benecke 1971). We aimed to answer questions regarding the metabolism (Fig. 2) by conducting feeding assays using ^13^C-labelled compounds with the model organism *C. vinula*.

## Materials and Methods

### General Information

HR-HPLC-ESI-MS was performed on an Agilent 1260 UPLC system; the system consisted of a quartenary pump G1311B, an autosampler G1367E, a column oven G1316A and a photodiode array detector G1315D (Agilent Technologies GmbH, Waldbronn, Germany) connected to a Bruker Compact QTOF mass spectrometer (Bruker Daltonics GmbH, Bremen, Germany). Standard parameters for small-molecule analysis were used as implemented in Bruker Compass version 1.9. The samples were measured in positive and negative ionization mode using a mass range of *m/z* 100 to *m/z* 700. NMR spectra were recorded on a Bruker Avance III HD 700 MHz spectrometer equipped with a cryoprobe and a 1.7 mm TCI microcryoprobe or on a Bruker Avance III HD 500 MHz NMR spectrometer equipped with a cryoprobe and a 5 mm TCI cryoprobe (Bruker Biospin GmbH, Rheinstetten, Germany). All NMR spectra were recorded at 298 K with MeOH-d_3_ as the solvent. Chemical shifts were referenced to the residual solvent peaks at δ_H_ 3.31 and δ_C_ 49.15. Data acquisition and processing was accomplished using Bruker TopSpin version 3.2. Standard pulse programs as implemented in Bruker TopSpin version 3.2 were used.

[U-^13^C]salicortin, [U-^13^C]HCH-salicortin and [U-^13^C]tremulacin were obtained as described previously (Schnurrer and Paetz 2023). Homogenization of plant material was carried out with a Bertin Minilys cell disruptor (Bertin Technologies, Montigny-le-Bretonneux, France). Chromatographic separation was accomplished using an Agilent 1100 HPLC system, which consisted of a degasser G1322A, a binary pump G1312A, an autosampler G1313A and a photodiode array detector G1315B (Agilent Technologies GmbH, Waldbronn, Germany). The column outlet was connected to an Advantec CHF122SB fraction collector (Advantec Toyo Kaisha Ltd., Tokyo, Japan) triggered by the HPLC via an G1351A Agilent relay contact board. HPLC separations were carried out using Macherey-Nagel columns (Macherey-Nagel, Düren, Germany). To purify [U-^13^C]trichocarpin and [U-^13^C]chlorogenic acid, a phenyl-hexyl-column (250 × 4.6 mm, 5 μm particle size) (Macherey-Nagel, Düren, Germany), was used. To purify [U-^13^C]neochlorogenic acid and [U-^13^C]chlorogenic acid methyl ester a π^2^-column (250 × 10 mm, 5 μm particle size, Macherey-Nagel, Düren, Germany) was used. Medium-pressure chromatographic (MPLC) separations were accomplished using a Biotage Isolera One chromatograph (Biotage Sweden AB, Uppsala, Sweden) using linear gradient elution on a 30 g Biotage Sfär C18 Duo column (solvents were H_2_O + 0.1% FA and MeOH + 0.1%FA). Detailed information on the purification procedures is given in the SI. To recover the compounds from the fractions, solvents were evaporated using a rotary evaporator (Büchi Rotavapor R-114, Büchi Labortechnik, Flawil, Switzerland). Solvents used for extraction and chromatographic separation were purchased from Carl Roth GmbH (Karlsruhe, Germany) and VWR International GmbH (Darmstadt, Germany), and used without further purification. The acetonitrile, water (LCMS grade) and formic acid (eluent additive for LC-MS) used for UHPLC-ESI-HRMS analyses were purchased from Merck KGaA (Darmstadt, Germany).Water used for HPLC separations was obtained from a Milli-Q Synthesis A 10 purifier (Merck KGaA, Darmstadt, Germany). HR-X SPE cartridges (500 mg sorbent/6 mL volume and 200 mg sorbent/3 mL volume), folded paper filters (90 mm) and paper disk filters (MN 615 ¼, 125 mm) were purchased from Macherey-Nagel. Syringe filters (0.45 μm, PA) were purchased from Carl Roth. Salicin, saligenin, hippuric acid, *o*-hydrohyhippuric acid, caffeic acid, protocatechuic acid, D-(−)-quinic acid, chlorogenic acid, and neochlorogenic acid were purchased from Merck KGaA, Darmstadt, Germany.

### Plant Material and Insect Larvae

Black poplar (*P. nigra*), hybrid trembling aspen (*P. tremula x tremuloides*), *P. deltoides x trichocarpa* plants (for rearing *C. vinula*) and *Physalis peruviana* were grown at the greenhouse facilities of the Max Planck Institute for Chemical Ecology in Jena, Germany. Puss moth larvae (*C. vinula*) for metabolic studies were taken from continuously reared insects maintained in the outdoor butterfly facility of the Max Planck Institute for Chemical Ecology in Jena, Germany.

### Isolation of [U-^13^C]Trichocarpin and [U-^13^C]CQAs

An extract (1.5 g) of ^13^C-labelled *P. deltoides x trichocarpa* leaf material, prepared as described previously (Schnurrer and Paetz 2023), was used to isolate the ^13^C-labelled compounds. After reconstitution with MeOH, aliquots (146.2 mg mL^− 1^) were subjected to HPLC separation on a Macherey-Nagel (MN) Isis RP-18e column (250 × 4.6 mm, 5 μm particle size) using the chromatographic conditions described previously (Schnurrer and Paetz 2023). Chlorogenic acid was eluted at *R*_t_ 27.3 min, trichocarpin at *R*_t_ 57.3 min, HCH-salicortin at *R*_t_ 62.0 min and tremulacin d at *R*_t_ 86.3 min. For monitoring, the UV trace at 285 nm was used. Compounds were subsequently re-purified to remove traces of impurities.

A binary solvent system consisting of 0.1% formic acid in H_2_O (solvent A) and 0.1% formic acid in MeOH (solvent B) was used. The HPLC column temperature was set to 35 °C and the flow rate was set to 0.8 ml min^− 1^. For analytical data describing the purified [U-^13^C]-labelled compounds, see the SI.

#### [U-^13^C]Trichocarpin

An aliquot solution (33.8 mg ml^− 1^) was purified with a MN phenyl-hexyl-column (250 × 4.6 mm, 5 μm particle size). The HPLC gradient started with a concentration of 30% solvent B and increased linearly to 60% B in 35 min. Afterwards, the column was washed for 10 min with 100% solvent B followed by an equilibration for 10 min with 30% B. The [U-^13^C]trichocarpin peak (λ = 285 nm) appeared at *R*_t_ 28.0. We isolated 12.88 mg of [U-^13^C]trichocarpin.

#### [U-^13^C]Chlorogenic acid

An aliquot solution (29.1 mg ml^− 1^) was purified with a MN phenyl-hexyl column (250 × 4.6 mm, 5 μm particle size). The HPLC elution was isocratic for 10 min at 40% B. Afterwards, the column was washed for 10 min with 100% B followed by an equilibration to 40% B for 10 min. The [U-^13^C]chlorogenic acid peak (λ = 285 nm) appeared at *R*_t_ 7.3 min. We isolated 3.1 mg of [U-^13^C]chlorogenic acid.

Salicortin, HCH-salicortin, tremulacin, trichocarpin and chlorogenic acid isolated from the ^13^C-labeled *P. deltoides x trichocarpa* tissue were analyzed by NMR (^1^ H, ^13^C and ^1^H-^13^C HSQC), and the acquired data were compared with previously published data. UHPLC-ESI-HRMS measurements were taken using a binary solvent system of H_2_O (solvent A) and acetonitrile (solvent B), both containing 0.1% (*v*/v) formic acid at a flow rate of 500 µl min^− 1^. The linear gradient started with 5% solvent B for 2 min and increased to 95% B within 28 min. Afterwards, the column was washed for 10 min with 95% solvent B followed by equilibration to 5% B for 5 min. ^13^C-enrichment was calculated from HRMS data (Jehmlich et al. 2010). The in vivo-generated [U-^13^C]-labelled compounds showed a total ^13^C-enrichment of > 75%. Detailed information about the calculation of the ^13^C-enrichment can be found in the SI.

### Isolation of [U-^13^C]Neochlorogenic Acid and [U-^13^C]Chlorogenic Acid Methyl Ester

An extract of lyophilized ^13^C-labeled *Physalis peruviana* leaf material (20.1 g) was prepared by MeOH extraction, and the solvent was evaporated in a vacuum. The crude extract was reconstituted in MeOH and subjected to MLPC (Isolera One, Biotage Sweden AB, Uppsala, Sweden) on MCI gel using a linear H_2_O (solvent A) - MeOH (solvent B) gradient (0-100%). The derived fractions were combined as indicated in the UV chromatogram, and aliquots of the combined MPLC fractions were subjected to UHPLC-ESI-HRMS analysis.

The fraction containing the title compounds (707.7 mg) was reconstituted with MeOH and subjected to MLPC (Isolera One, Biotage Sfär C18 Duo column) separation. The gradient started with 0% B and increased linearly to 20% B over 15 min. At 20% B, the gradient was kept isocratic for 30 min until no UV-absorbing compounds were eluted. Afterwards the column was rinsed with 100% B. The derived fractions were combined as indicated in the UV-chromatogram, and aliquots were again analyzed by UHPLC-ESI-HRMS. Fraction 2 (30.8 mg) contained neochlorogenic acid, while fraction 3 (69.7 mg) contained chlorogenic acid methyl ester.

#### [U-^13^C]Neochlorogenic Acid

An aliquot solution (102.7 mg ml^− 1^) of fraction 2 was purified with a MN π^2^-column (250 × 10 mm, 5 μm particle size). The HPLC gradient started with a concentration of 5% B and increased linearly to 20% B in 30 min. Afterwards the column was washed for 10 min with 100% B and subsequently equilibrated for 10 min with 5% B. The [U-^13^C]neochlorogenic acid peak (λ = 285 nm) appeared at *R*_t_ 22.1 min. We isolated 4.2 mg of [U-^13^C]neochlorogenic acid.

#### [U-^13^C]Chlorogenic Acid Methyl Ester

An aliquot solution (87.1 mg ml^− 1^) of fraction 3 was purified with an MN π^2^-column (250 × 10 mm, 5 μm particle size). The compound was purified using 10% B for 45 min. Afterwards, the column was washed for 10 min with 100% B followed by equilibration for 10 min with 10% B. The [U-^13^C]chlorogenic acid methyl ester peak (λ = 285 nm) appeared at *R*_t_ 41.1 min. We isolated 11.7 mg of [U-^13^C]chlorogenic acid methyl ester. The characterization of the ^13^C-labeled neochlorogenic acid and chlorogenic acid methyl ester was accomplished as described above. The in vivo-generated neochlorogenic acid and chlorogenic acid methyl ester showed a ^13^C-labeling > 24%. For spectroscopic data, see the SI.

### Feeding Assays with *C. vinula*

Freshly cropped leaves of *P. nigra* and *P. tremula x tremuloides* were used for feeding assays. The stalk of each leaf was placed in an Eppendorf micro reaction vessel filled with 1.5 ml tap water and sealed with parafilm to hold the stalk in place (for details, see the SI). Methanolic solutions of either [U-^13^C]salicortin (5 mg ml^− 1^), [U-^13^C]HCH-salicortin (5 mg ml^− 1^), [U-^13^C]tremulacin (5 mg ml^− 1^), [U-^13^C]trichocarpin (2.5 mg ml^− 1^), [U-^13^C]neochlorogenic acid (2.5 mg ml^− 1^), [U-^13^C]chlorogenic acid (2.5 mg ml^− 1^) or [U-^13^C]chlorogenic acid methyl ester (2.5 mg ml^− 1^) were dispersed evenly on the surface of five poplar leaves (200 µl solution per leaf) using a pipette. Control leaves were supplemented with the same volume of MeOH. To exclude the possibility of degradation during the feeding trial, the stability of salicortin was tested in a separate assay (see SI). After solvents evaporated, the leaves were photographed to determine area. For the assay, *C. vinula* larvae (3rd and 4th instar) were reared for 24 h on either of the two poplar species, followed by a period of starvation (6 h) prior to feeding. For the feeding assay, one *C. vinula* larva (early 4th instar or late 3rd instar for [U-^13^C] trichocarpin) per leaf was placed into an Aldrich Phytacon vessel (Merck KGaA, Darmstadt, Germany). Each feeding assay was replicated five times. The larvae were taken from the vessel 12 h after the leaves were fully consumed. Frass released during the assays was collected, pooled, and immediately dried in a vacuum.

### Extraction of *C. vinula* Frass

Desiccated frass samples were extracted with MeOH (5 × 5 ml) using a Bertin Minilys cell disruptor. Tubes of 7 ml volume, equipped with 1.4 mm o.d. ZrO_2_ beads, were shaken for 60 s at 4000 rpm. After centrifugation, the supernatants of each sample were pooled and finally centrifuged in 15 ml Falcon tubes for 10 min at 6000 rpm. The supernatants were then transferred into round-bottom glass flasks, and the solvent was removed in a vacuum at room temperature by means of a rotary evaporator. The crude extract from chlorogenic acid feeding assays was reconstituted with MeOH (20 mL) and passed through an MN HR-X SPE cartridge (200 mg/3mL) to remove chlorophyll and other highly lipophilic compounds; the filtrate was evaporated with N_2_ gas. Crude extracts from salicortinoid and trichocarpin feeding assays were subjected to pre-separation on MN HR-X SPE cartridges (500 mg/6 mL) applying the protocol described previously (Feistel et al. 2017). The methanolic and aqueous eluates were dried by N_2_ gas, resulting in pre-purified extracts (for masses, see the SI). Subsequently, extract samples from all feeding and control assays were subjected to UHPLC-ESI-HRMS analysis.

### Metabolite Isolation from *C. vinula* Frass Extracts

An aliquot of the pre-purified frass extract, reconstituted with MeOH (37.4 mg ml^− 1^), was separated by HPLC using an MN Isis RP-18e column (250 × 4.6 mm, 5 μm particle size). The column temperature was set to 35 °C and the solvent flow rate was 0.8 ml min^− 1^. A binary solvent system of water (solvent A) and MeOH (solvent B), both containing 0.1% *v/v* formic acid, was used. After an initial isocratic period of 5 min with 0% B, the gradient reached 50% B after 90 min and increased further to 80% B after 115 min. The column was then washed with 100% B for 5 min, after which an equilibration period of 10 min with 0% solvent B preceded the subsequent run. The column outlet was connected to a fraction collector; all separated peaks were collected. Eluted compounds were dried by N_2_ gas at room temperature. A detailed list of the isolated compounds can be found in the SI. All structures were confirmed by NMR spectroscopy and UHPLC-ESI-HRMS.

## Results

With the present study, we aimed to determine the sources of quinates and benzoates as part of the metabolites in the frass of Salicaceae-adapted lepidopteran herbivores of the Notodontidae. We assumed that quinates originate from CQAs and benzoates from other defense substances such as the salicortinoid tremulacin or the salicylate trichocarpin. As in previous metabolism studies (Feistel et al. 2018) (Schnurrer and Paetz 2023), we performed feeding assays using ^13^C-labelled compounds. For this series of assays, the leaves of two poplar species, *P. tremula x tremuloides* and *P. nigra*, were supplemented with the ^13^C-labelled compounds and then fed to the model organism *C. vinula*. We analyzed the metabolites in the frass and found the composition of metabolites to be very similar. All identified compounds were derived from salicinoids/salicortinoids and flavonoids. The main difference between metabolites from the *P. nigra* and *P. tremula x tremuloides* assays was in the composition of flavonoids. All flavonoids were found as glycosides. The flavonoids 3-*O*-glycosyl-2’-*O*-xylosyl-6’-*O*-rhamnosyl-quercetin, 3-*O*-glycosyl-2’-*O*-xylosyl-quercetin and kaempferol-3-*O*-glucoronide were found only as metabolites from *P. tremula x tremuloides.* Quercetin-3-*O*-glucoside and quercetin-3-*O*-glucuronide were found in both poplar species. Rutin was absent in the frass from larvae fed on *P. tremula x tremuloides*. ^13^C-labelling was found only in salicinoids and salicortinoids. A detailed list of metabolites with analytical data can be found in the SI.

### Analysis of [U-^13^C]CQA Metabolites in Larvae Fed on *P. nigra*

After feeding leaves supplemented with the [U-^13^C]CQAs neochlorogenic acid, chlorogenic acid, and [U-^13^C]chlorogenic acid methyl ester to the larvae, we found ^13^C-labelling in metabolites of the frass of *C. vinula*. We identified three fully ^13^C-labelled metabolites: quinic acid (**1**), caffeic acid (**9**) and protocatechuic acid (PCA) (**3**). The former two acids were the ester hydrolysis products of CQAs. We also identified caffeic acid-4-*O*-glucoside (**4**) by comparison with a synthesized reference standard. Traces of an additional structure similar to caffeic acid glucoside were putatively annotated as caffeic acid-3-*O*-glucoside (**6**) and caffeic acid glucosyl ester (**5**). Another trace compound was putatively annotated as protocatechuic acid glucoside (**2**). Low amounts of the aforementioned compounds prevented us from being able to isolate them for NMR structure elucidation, and therefore the definite position for the glucoside substituents could not be determined. Other structures resulting from caffeate were not detected. The remaining 16 partially ^13^C-labelled metabolites are listed in Table 1. All identified mono- and bis-salicyloyl/benzoyl quinic acid conjugates (**7**,**8**,**10**–**19**) consisted of ^13^C-labelling in the quinate part of the molecule. Quinic acid (**1**) released from the CQAs was identified as the source of the quinates in the conjugates (SI Fig.:**275**–**279**). The externally applied CQAs were not fully metabolized by the larvae during digestion. Therefore, we observed a complex mixture of different CQA isomers as by-products in the frass extracts.

**Table 1:** ^13^C-labelled metabolites from *C. vinula* frass after larvae were feeding on *P. nigra* leaves supplemented with [U-^13^C]CQAs. (++) indicate completely ^13^C-labelled metabolites, (+) partially ^13^C-labelled metabolites. PCA: protocatechuic acid (3,4-dihydroxybenzoic acid), *) putatively annotated

No.	R_t_ in min	metabolite	^13^C-labelling after supplementing with			SI-Fig.:
			[U-^3^C]neo-chlorogenic acid	[U-^3^ C]chlorogenic acid	[U-^13^C] chlorogenic acid methyl ester	
1	3.2	quinic acid	++	++	++	194
2	9.6	PCA glucoside*	+	+	+	196
3	10.3	PCA	++	++	++	195
4	10.9	caffeic acid-4-*O*-glucoside	+	+	+	198
5	11.2	caffeic acid glucosyl ester*	+	+	+	
6	11.6	caffeic acid-3-*O*-glucoside	+	+	+	199
7	11.7	3-*O*-benzoylquinic acid	+	+	+	200
8	12.8	3-*O*-salicyloylquinic acid	+	+	+	203
9	13.0	caffeic acid	++	++	++	197
10	13.4	4-*O*-benzoylquinic acid	+	+	+	201
11	14.0	5-*O*-benzoylquinic acid	+	+	+	202
12	14.2	4-*O*-salicyloylquinic acid	+	+	+	204
13	14.6	5-*O*-salicyloylquinic acid	+	+	+	205
14	19.9	3,4-*O*-bisbenzoylquinic acid	+	+	+	206
14	19.9	3,5-*O*-bisbenzoylquinic acid	+	+	+	
15	20.4	3-*O*-salicyloyl-4-*O*-benzoylquinic acid	+	+	+	208
15	20.4	4-*O*-salicyloyl-5-*O*-benzoylquinic acid	+	+	+	
16	20.9	3,4-*O*-bissalicyloylquinic acid	+	+	+	210
16	20.9	3,5-*O*-bissalicyloylquinic acid	+	+	+	
17	21.2	4,5-*O*-bisbenzoylquinic acid	+	+	+	207
18	21.7	3-*O*-salicoyl-5-*O*-benzoylquinic acid	+	+	+	209
19	22.2	4,5-*O*-bissalicyloylquinic acid	+	+	+	211

### Analysis of [U-^13^C]salicortinoid Metabolites in Larvae Fed on *P. nigra*

We identified 28 ^13^C-labelled metabolites in frass from larvae fed on *P. nigra*. The abundance of ^13^C-labeling in the metabolites is shown in Table 2. We did not find any ^13^C-labelling in the flavonoids. The metabolites salicin (**1**), saligenin (**5**) and salicylic acid (**17**) were completely ^13^C-labelled and must therefore be products resulting from hydrolysis or oxidation. A part of the salicin seems to result from re-glucosylated saligenin, because the labelling pattern differed from that of the salicin fragment in salicortinoids. In comparison to the latter, salicin isolated from frass showed two pronounced peaks in the labelling pattern, one at [M-H+7]^−^ and one at [M-H+13]^−^. Such a pattern typifies the recombination products of two ^13^C-labelled compounds. Similarly, tremuloidin (**16**) and populin (**18**) were rearranged after feeding larvae [U-^13^C]tremulacin. Both metabolites were fully ^13^C-labelled but also show an additional labelling peak at [M-H+7]^−^, corresponding to a benzoyl moiety. In contrast, after [U-^13^C]salicortin and [U-^13^C]HCH-salicortin were fed to larvae, only the salicin in **16** and **18** was ^13^C-labelled. Metabolite **2** was identified as isosalicin. Interestingly, this compound was mainly ^13^C-labelled in the glucosyl moiety, as indicated by ^13^C-satellite signals in the ^1^H-NMR spectrum and by the isotopic pattern of the glucosyl fragment in the MS spectrum. Another glycosylated metabolite was catechol glucoside (**3**), but in contrast to **2**, the major portion of ^13^C-labelling occurred in the phenolic part of the molecule. Hippuric acid (**6**) and *o*-hydroxyhippuric acid (**13**) are glycine conjugates of benzoic acid and salicylic acid, respectively. ^13^C-labelling in **6** exclusively occurred after feeding larvae [U-^13^C]tremulacin, while ^13^C-labelling in **13** was present after larvae were fed the other [U-^13^C]salicortinoids. Additionally, we detected *N*-salicyloylalanine (**15**) and *N*-benzoylalanine (**10**) as amino acid conjugates. Because both compounds were formed in low amounts, their annotation is not definitive. The compounds **5**, **13**, **14**, **23**, **24** and **27**, mono- and bis-substituted salicyloylquinic acid esters, were previously described as metabolites from *C. vinula* (Feistel et al. 2018). In accordance with previous results, ^13^C-labelling occurred only in the salicyloyl part of the molecules (SI Figs. 276 and 278). ^13^C-labelled salicyloyl units were also present in mixed salicyloyl-benzoyl quinic acids **21**, **22** and **26** (SI Fig. 279). Additionally, the frass of larvae fed [U-^13^C]tremulacin contained ^13^C-labelled benzoyl moieties in the mixed quinic acid esters and the monosubstituted benzoyl-quinic acid esters **8** and **10** (SI Figs. 275 and 279).

**Table 2:** ^13^C-labelled metabolites from *C. vinula* frass after larvae were feeding on *P. nigra* leaves supplemented with [U-^13^C]salicortinoids. (++) indicate completely ^13^C-labelled metabolites, (+) partially ^13^C-labelled metabolites, (-) not detected, *) putatively annotated

	R_t_ in min	metabolite	^13^C-labelling after supplementing with			SI-Fig.:
			[U-^13^C] Salicortin	[U-^13^C] HCH-Salicortin	[U-^13^C] tremulacin	
1	10.3	salicin	++	++	++	212
2	11.0	isosalicin	+	+	+	213/214
3	11.0	catechol glucoside	+	+	+	215
4	11.6	3-*O*-benzoylquinic acid	-	-	+	216
5	12.2	saligenin	++	++	++	233
6	12.6	hippuric acid	-	-	+	234
7	12.7	3-*O*-salicyloylquinic acid	+	+	+	219
8	13.2	4-*O*-benzoylquinic acid	-	-	+	217
9	13.7	5-*O*-benzoylquinic acid	-	-	+	218
10	14.1	*N*-benzoylalanine*	-	-	-	241
11	14.1	4-*O*-salicyloylquinic acid	+	+	+	220
12	14.3	5-*O*-salicyloylquinic acid	+	+	+	221
13	14.5	*o*-hydroxyhippuric acid	+	+	+	235
14	15.1	nigracin	-	-	+	243
15	16.2	*N*-salicyloylalanine*	+	+	+	242
16	16.5	tremuloidin	-	-	++	244
17	17.5	salicylic acid	++	++	++	232
18	17.6	populin	+	+	++	231
19	18.8	tremulacinol*	-	-	++	236
20	19.6	3,4-*O*-bisbenzoylquinic acid	-	-	+	222
21	19.7	3,5-*O*-bisbenzoylquinic acid	-	-	+	223
22	20.2	3-*O*-salicyloyl-4-*O*-benzoylquinic acid	+	+	+	225
23	20.4	4-*O*-salicyloyl-5-*O*-benzoylquinic acid	+	+	+	226
24	20.7	3,4-*O*-bissalicyloylquinic acid	+	+	+	228
25	21.0	4,5-*O*-bisbenzoylquinic acid	+	+	+	224
25	21.0	3,5-*O*-salicyloylquinic acid	+	+	+	229
26	21.6	3-*O*-salicyloyl-5-*O*-benzoylquinic acid	+	+	+	227
27	22.0	4,5-*O*-salicyloylquinic acid	+	+	+	230
28	22.6	2’,6’-*O*-bisbenzoylsalicin	-	-	++	246
w1	8.6	DHCH glucoside*	+	+	+	240
w2	9.1	saligenin sulfate*	+	+	+	237
w3	10.2	catechol sulfate*	+	+	+	238
w4	10.4	*o*-hydroxyhippuric acid glucoside*	+	+	+	239

The molecular formula of compound **4** was elucidated as C_14_H_16_O_7_ (calcd. for C_14_H_15_O_7_, *m/z* 295.0823, measured *m/z* 295.0829 [M-H]^−^) by HRESIMS. The MS spectrum showed a ^13^C-labelled benzoyl unit, which indicated a compound similar to previously reported derivatives (Feistel et al. 2018). Using NMR spectroscopy, **4** was identified as 3-*O*-benzoylquinic acid. For a detailed description of the elucidation, see the SI. Metabolite **19** has a molecular formula of C_21_H_19_O_8_, as determined by HRESIMS (calcd. for C_21_H_19_O_8_, *m/z* 399.1085, found *m*/*z* 399.1082 [M-H]^−^). NMR spectroscopy led to the identification of **19** as 3,4-*O*-bisbenzoylquinic acid (see SI). The HRESIMS spectra of compounds **20** and **24** showed pseudo-molecular ions at *m/z* 399.1071 [M-H]^−^ and 399.1073 [M-H]^−^, respectively, matching the molecular formula of C_21_H_19_O_8_ (calcd. for C_21_H_19_O_8_, *m/z* 399.1085). NMR spectroscopy revealed **20** to be 3,5-*O*-bisbenzoyl-quinic acid, and **24** was identified as 4,5-*O*-bisbenzoyl-quinic acid (see the SI). Benzoyl-substituted quinic acids contained only a ^13^C-label after feeding larvae leaves supplemented with [U-^13^C]-tremulacin (SI Fig. 277). There was no detectable ^13^C-label in benzoylated metabolites after larvae were fed [U-^13^C]salicortin or [U-^13^C]HCH-salicortin. After supplementing leaves with [U-^13^C]tremulacin, we found in larvae fed on them, the key intermediate tremulacinol (**19**)(Schnurrer and Paetz 2023); tremulacinol’s labelling pattern resembled that of the parent compound.

Among the very polar compounds in the water eluate from the initial SPE fractionation, we found four ^13^C-labelled metabolites for all tested [U-^13^C]salicortinoids. We identified saligenin sulfate and catechol sulfate based on their fragmentation pattern. Furthermore, we found a metabolite *m/z* of 275.1159 (calc. for C_12_H_19_O_7_, *m/z* 275.1136) with a [M-H+6] peak. We identified this compound as 1,2-dihydroxy-cyclohex-3-ene-glucoside (DHCH-glucoside), a decarboxylated DHCH, glycosylated on one of its remaining hydroxyl functions. Another metabolite with an *m/z* of 356.0992 (calcd. for C_15_H_18_NO_9_, *m/z* 356.0987) and a [M-H+7] peak was identified as *o*-hydroxyhippuric acid glucoside based on its fragmentation pattern. A detailed overview of the salicortinoid metabolism on *P. nigra* can be found in the SI.

### Analysis of [U-^13^C]Salicortinoid Metabolites in Larvae Feeding on *P. tremula x tremuloides*

When ^13^C-supplementing assays were carried out with larvae fed the leaves of *P. tremula x tremuloides*, we identified 25 metabolites (Table 3). Full ^13^C-labelling was found for salicin (**1**), the catechol moiety in (**2**), the salicyloyl units in **8**–**17** and salicylic acid (**5**). Interestingly, isosalicin and saligenin were absent. As in the assays with *P. nigra*, ^13^C-labelling in benzoyl units occurred only after larvae were fed leaves supplemented with [U-^13^C]tremulacin. An additional abundant metabolite with full ^13^C-labelling was determined as 2’,6’-*O-*bisbenzoylsalicin (**17**). Here, the salicin part was ^13^C-labelled after feeding larvae leaves supplemented with [U-^13^C]salicortin, whereas no ^13^C-label could be found after feeding them leaves supplemented with [U-^13^C]HCH-salicortin. The water eluate of the initial solid phase extraction (SPE) sample preparation contained almost the same metabolites as elucidated in the *P. nigra* assays. We also found traces of fully ^13^C-labelled 1,6-dihydroxycyclohex-2-ene-1-carboxylic acid (DHCH), as indicated by its molecular ion of [M-H+7] for all tested [U-^13^C]salicortinoids. DHCH is a relatively stable molecule compared to the HCH-moiety found in salicortinoids. A detailed overview of the salicortinoid metabolism on *P.tremula x tremuloides* can be found in the SI (SI Figs. 306 and 307).

**Table 3:** ^13^C-labelled metabolites from *C. vinula* frass after larvae were feeding on *P. tremula x tremuloides* leaves supplemented with [U-^13^C]salicortinoids. (++) indicates completely ^13^C-labelled metabolites, (+) partially ^13^C-labelled metabolites, (-) not detected. *) putatively annotated

	R_t_ in min	metabolite	^13^C labelling after supplementing with			SI-Fig.:
			[U-^13^C]salicortin	[U-^13^C]HCH-salicortin	[U-^13^C]tremulacin	
1	10.3	salicin (**1**)	**++**	**++**	**++**	246
2	11.0	catechol glucoside (**2**)	**+**	**+**	**+**	247
3	11.6	3-*O*-benzoylquinic acid (**19**)	**-**	**-**	**+**	248
4	12.6	hippuric acid (**18**)	**-**	**-**	**+**	263
5	12.7	3-*O*-salicyloylquinic acid (**8**)	**+**	**+**	**+**	251
6	13.2	4-*O*-benzoylquinic acid (**20**)	**-**	**-**	**+**	249
7	13.7	5-*O*-benzoylquinic acid (**21**)	**-**	**-**	**+**	250
8	14.1	4-*O*-salicyloylquinic acid (**9**)	**+**	**+**	**+**	252
9	14.1	*N*-benzoylalanine*	**-**	**-**	**+**	264
10	14.3	5-*O*-salicyloylquinic acid (**10**)	**+**	**+**	**+**	253
11	14.5	*o*-hydroxyhippuric acid (**6**)	**+**	**+**	**+**	265
12	16.2	*N*-salicyloylalanine*	**+**	**+**	**+**	266
13	16.5	tremuloidin (**25**)	**-**	**-**	**++**	268
14	17.5	salicylic acid (**5**)	**++**	**++**	**++**	267
15	17.6	populin (**3**)	**+**	**+**	**++**	269
16	19.6	3,4-*O*-bisbenzoylquinic acid (**22**)	**-**	**-**	**+**	254
17	19.7	3,5-*O*-bisbenzoylquinic acid (**23**)	**-**	**-**	**+**	255
18	20.2	3-*O*-salicyloyl-4-*O*-benzoylquinic acid (**11**)	**+**	**+**	**+**	257
19	20.4	4-*O*-salicyloyl-5-*O*-benzoylquinic acid (**12**)	**+**	**+**	**+**	258
20	20.7	3,4-*O*-bissalicyloylquinic acid (**14**)	**+**	**+**	**+**	260
21	21.0	4,5-*O*-bisbenzoylquinic acid (**24**)	**+**	**+**	**+**	256
22	21.0	3,5-*O*-bissalicyloylquinic acid (**15**)	**+**	**+**	**+**	261
23	21.6	3-*O*-salicyloyl-5-*O*-benzoylquinic acid (**13**)	**+**	**+**	**+**	259
24	22.0	4,5-*O*-bissalicyloylquinic acid (**16**)	**+**	**+**	**+**	262
25	22.6	2’,6’-*O-*bisbenzoylsalicin (**17**)	**+**	**-**	**++**	270
w1	6.3	DHCH	**++**	**++**	**++**	271
w2	9.1	saligenin sulfate*	**+**	**+**	**+**	272
w3	10.2	catechol sulfate*	**+**	**+**	**+**	273
w4	10.4	*o*-hydroxyhippuric acid glucoside*	**+**	**+**	**+**	274

### Analysis of [U-^13^C]Trichocarpin Metabolites in Larvae Feeding on *P. nigra* and *P. tremula x tremuloides*

The largest part of [U-^13^C]trichocarpin was excreted unchanged in both the *P. nigra* and *P. tremula x tremuloides* assays. Therefore, the amount of ^13^C incorporated into metabolites was low. The metabolites identified are listed in Table 4. An exception was the deglucosylated product benzyl-gentisate (**13**), which occurred only with *P. tremula x tremuloides*, showing a [M-H+14]^−^ peak with similar intensity in the parent compound. Other metabolites with higher ^13^C-incorporation included a putatively annotated trichocarpin isomer (**5**) and benzoyl-trichocarpin (**10**). Both metabolites were low in abundance. Because **5** had the same [M-H+14]^−^ labelling pattern as benzyl gentisate, we propose **5** to be isotrichocarpin. Compound **10** showed the same [M-H+20] pattern as trichocarpin and therefore must contain a benzoyl substituent. Based on observations from previous assays, we propose the 6’-OH position to be benzoylated. ^13^C-labelled gentisic acid, benzylalcohol and benzoic acid were absent; however, we observed ^13^C-labelling in quinic acid conjugates with at least one benzoyl substituent. We could also putatively annotate a compound with an [M-H+7]^−^-labeling pattern as gentisic acid glucoside, and we conclude the latter to be result of gentisic acid re-glucosylation. Unfortunately, we could not isolate a sufficient amount for NMR structure elucidation. Therefore, the position of glucosylation remains unspecified. A detailed overview of trichocarpin metabolism is given in the SI (SI Fig. 308).

**Table 4:** ^13^C-labelled metabolites from *C. vinula* frass after larvae were feeding on *P. nigra* and *P. tremula x tremuloides* leaves supplemented with [U-^13^C]trichocarpin. (++) indicates completely ^13^C-labelled metabolites, (+) partially ^13^C-labelled metabolites, (-) not detected

	R_t_ in min	metabolite	^13^C labelling after feeding with [U-^13^C]trichocarpin on		SI-Fig.:
			*P. nigra*	*P. tremula x tremuloides*	
1	11.6	3-*O*-benzoylquinic acid (**19**)	**+**	**+**	280/293
2	13.2	4-*O*-benzoylquinic acid (**20**)	**+**	**+**	281/294
3	13.7	5-*O*-benzoylquinic acid (**21**)	**+**	**+**	282/295
4	14.1	trichocarpin	**++**	**++**	291
5	17.8	isotrichocarpin	**+**	**+**	289/300
6	19.6	3,4-*O*-bisbenzoylquinic acid (**22**)	**+**	**+**	283/296
7	19.7	3,5-*O*-bisbenzoylquinic acid (**23**)	**+**	**+**	284/297
8	20.2	3-*O*-salicyloyl-4-*O*-benzoylquinic acid (**11**)	**+**	**+**	286/299
9	20.4	4-*O*-salicyloyl-5-*O*-benzoylquinic acid (**12**)	**+**	**+**	287/300
10	20.6	6’-OH-benzoyltrichocarpin (**14**)	**+**	**+**	290/303
11	21.0	4,5-*O*-bisbenzoylquinic acid (**24**)	**+**	**+**	285/296
12	21.0	3,5-*O*-salicyloylquinic acid (**15**)	**+**	**+**	288/301
13	21.6	benzyl gentisate (**13**)	**-**	**++**	304
w1	9.1	gentisic acid glucoside*	**+**	**-**	292

## Discussion

*Cerura vinula* larvae, specialist herbivores adapted to Salicaceae, have developed a unique metabolism to counteract the chemical defenses of their host plants. The caterpillars have the ability to convert salicortinoids — complex salicinoids with a HCH moiety — into mono- and bis-salicyloyl quinic acids, mono-benzoyl quinic acids and mixed salicyloyl-benzoyl quinic acids (Feistel et al. 2018). Feeding larvae leaves supplemented with the stable isotopes showed that salicortin is reductively converted to saligenin and DHCH, which are the source of salicyloyl substituents in the metabolites recovered from frass (Schnurrer and Paetz 2023). However, the origin of benzoic acid, which forms benzoyl quinate adducts, and the origin of quinic acid were still unknown (Fig. 2). Because previous studies identified CQAs as abundant feeding deterrents in Salicaceae, we hypothesized that CQAs such as chlorogenic acid were the source of the quinic acid (Matsuda and Senbo 1986) (Molgaard and Ravn 1988). In addition, we assumed that the benzoyl substituents in salicortinoids and other complex salicinoids were the source of the benzoates. We therefore investigated the metabolism of different [U-^13^C]CQAs to prove our hypothesis. In addition, we fed caterpillars with leaves supplemented with the benzoylated salicortinoid [U-^13^C]tremulacin and the salicylate [U-^13^C]trichocarpin (the latter is an indirect source of benzoate) to determine whether such compounds are responsible for the formation of benzoylated quinates in the frass of the caterpillars. Finally, we wanted to clarify to what extent enzymatic transformations originating from leaf chemistry influence our picture of the metabolism in the model organism *C. vinula*. We investigated the transformation of ^13^C-labelled compounds applied to leaves of two poplar species, *P. nigra* and *P. tremula x tremuloides*, and performed in-depth analyses of the resulting metabolites.

### CQAs are the Source of Quinates in the Metabolic End-Products of *C. vinula*

**Fig. 3:**
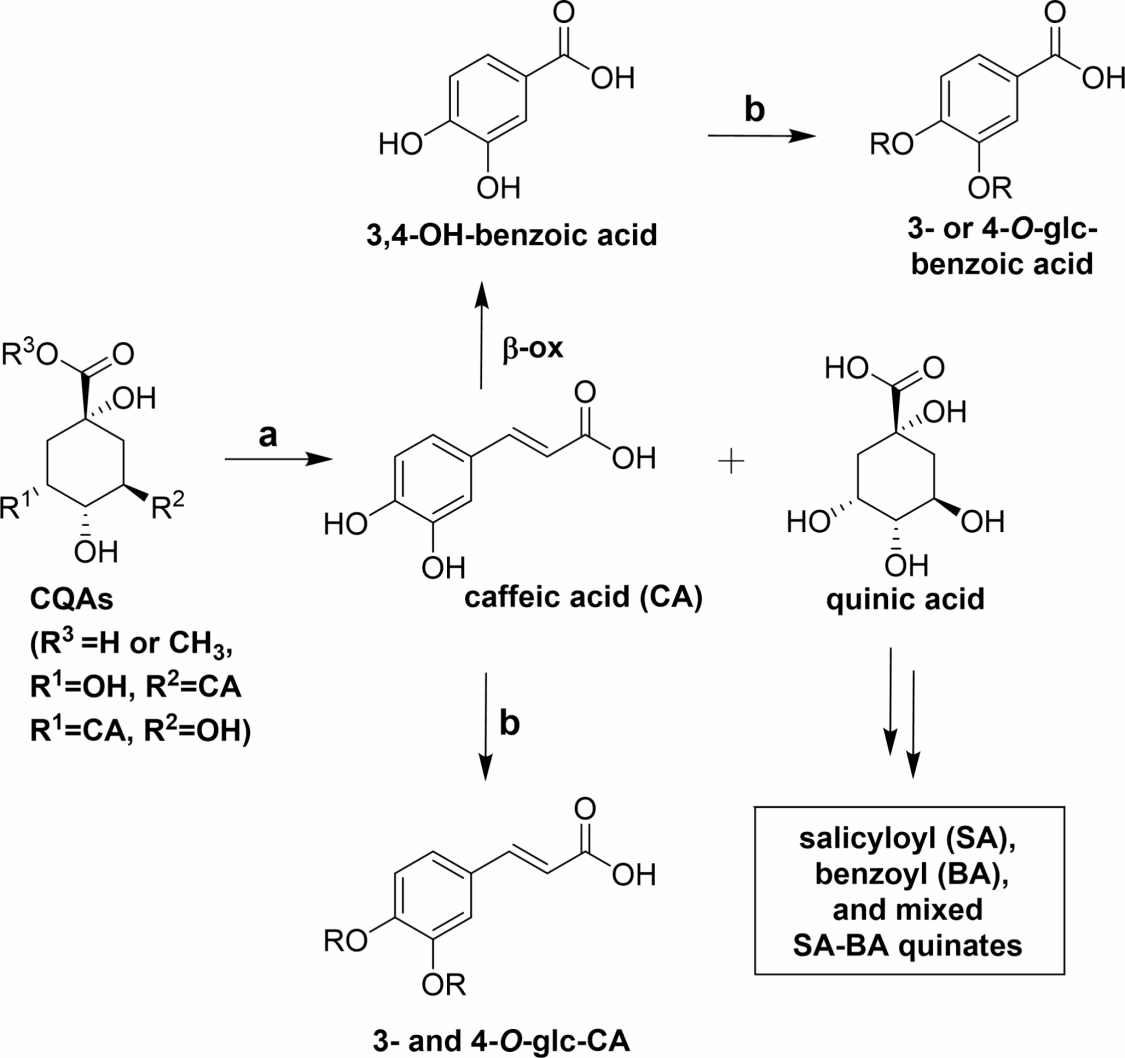
CQA transformation observed in *C. vinula*. **a**, carboxylesterase; **b**, UDPG, glucosyltransferase; **β-ox**, β-oxidative pathway; Glc, glucosyl

After feeding [U-^13^C]CQAs supplemented leaves to *C. vinula* larvae, we observed ^13^C-labelling in 19 metabolites (Table 1) of their frass. Fully ^13^C-labelled were quinic acid, caffeic acid and protocatechuic acid, PCA. Quinic acid and caffeic acid resulted from the enzymatic hydrolysis of the CQA’s ester bonds. Hydrolysis is unlikely to occur spontaneously, as it has been reported that alkaline intestinal conditions mainly lead to the isomerization of CQAs (Vihakas et al. 2015; Xie et al. 2011). PCA was likely formed via the *β*-oxidative pathway, a transformation previously described only in fungi (Lubbers et al. 2021). We also identified mono-glucosylated derivatives of PCA and caffeic acid. Using reference standard, we identified caffeic acid-4-*O*-glucoside. All mono-/bissalicyloylquinic acids, mono-/bisbenzoylquinic acids and mixed salicyloyl-benzoylquinic acids were exclusively ^13^C-labelled in the quinic acid part. We assume that both quinic acids generated from CQAs and also free quinic acid can be incorporated into the metabolic end-products. The observed reactions are summarized in Fig. 3.

### Tremulacin and Trichocarpin are Precursors for Benzoylated Quinic Acids in *C. vinula*

Benzoylated quinic acids and mixed salicyloyl-benzoyl quinic acids are among the metabolic end-products of *C. vinula* larvae (Feistel et al. 2018). We hypothesized that the benzoyl substituents originate from plant metabolites; therefore we used [U-^13^C]tremulacin and [U-^13^C]trichocarpin as molecular probes to test our hypothesis. Analysis of frass metabolites after feeding the larvae poplar leaves supplemented with ^13^C-labelled compounds provided evidence that our hypothesis was correct (Tables 3 and 4). The hydrolyzable aromatic moiety in both probes, benzoic acid for tremulacin and benzyl alcohol for trichocarpin, was later present as benzoyl substituent in quinic acid adducts. Therefore, benzyl alcohol must have been oxidatively converted to benzoic acid. We also assume that the cleavage of benzoyl or benzyl groups from the precursors is the result of an enzymatic reaction. According to this interpretation, most of the trichocarpin was excreted unmetabolized because this molecule is a structurally unsuitable substrate for the enzymatic arsenal of *C. vinula*. Consequently, ester hydrolysis in *C. vinula* must be selective and structure-dependent. Because benzoic acid and benzyl alcohol only appear in conjugates among the metabolic end-products, we consider them to have a negative effect on the metabolism of *C. vinula*. The detailed metabolism of trichocarpin is summarized in the SI (SI Fig. 308).

### A General View on the Metabolism of Salicortinoids by *C. vinula*

**Fig. 4:**
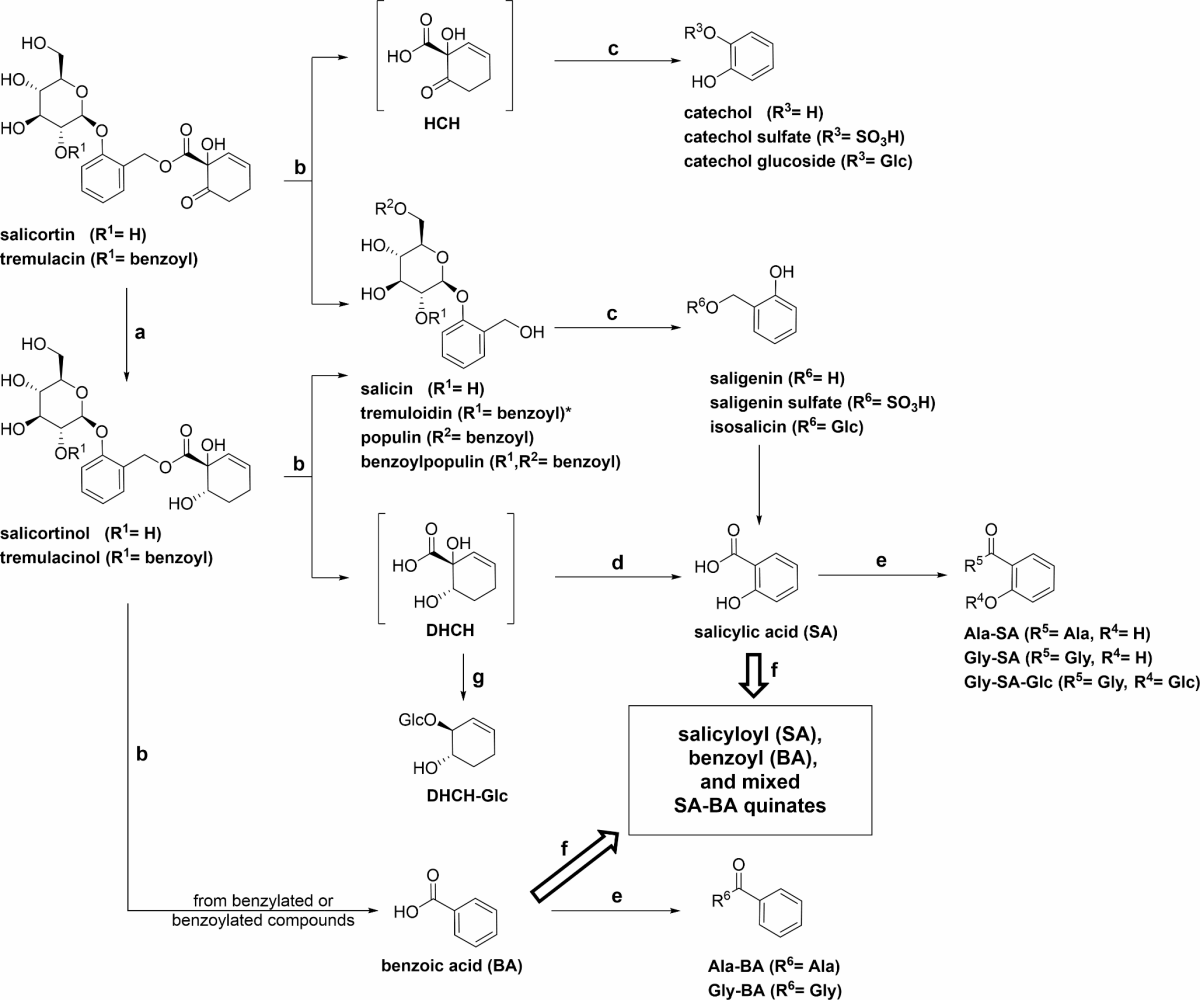
Overview of ^13^C-labelled salicortinoid metabolites from *C. vinula* larvae fed *P. nigra* and *P. tremula x tremuloides*. Product-precursor relationships are represented by arrows. Metabolic transformations were assumed to proceed as follows: **a**, aldehyde reductase, NADPH; **b**, carboxylesterase, or carboxylesterase, CYP450; **c**, CYP450, PAPS, sulfotransferase, or CYP450, UDPG, glucosyltransferase; **d**, CYP450; **e**, CoA, CoA transferase, amino acid; **f**, CoA, CoA transferase, quinate; **g**, oxidoreductase, UDPG, glucosyltransferase. Tremuloidin (*) was observed only after tremulacin was fed to the larvae. Glc: glycosyl

Our results extend the knowledge of salicinoid/salicortinoid metabolism in herbivores, especially for Salicaceae-adapted Notodontidae (Boeckler et al. 2011, 2016; Feistel et al. 2018; Schnurrer and Paetz 2023). Feeding assays with *C. vinula* larvae using ^13^C-labelled salicortin and tremulacin to supplement leaves of two different poplar species, *P. nigra* and *P. tremula x tremuloides*, shed light on metabolism in our model species. The constituents of the two poplar species differ mainly in their content of benzoylated compounds: *P. tremula x tremuloides* is rich in tremulacin, a benzoylated derivative of salicortin (Abreu et al. 2011). The results of the assays are summarized in Fig. 4, and a detailed overview of the product-precursor relationships of the metabolism can be found in the SI (SI Fig. 306 and SI Fig. 307). The metabolism of salicortinoids is highly similar for both poplar species. The two salicortinoids were reductively degraded as described previously (Schnurrer and Paetz 2023). Only a few differences were found in the salicortinoid metabolites, and these were mainly due to the different abundances of benzoylated leaf constituents. We identified catechol glucoside and, for the first time, catechol sulfate as metabolites in *C. vinula*. These results are similar to those found for the generalist herbivore *L. dispar* (Boeckler et al. 2016). We also identified other HCH-metabolites from the degradation of reduced salicortinoids, namely DHCH, a precursor of salicylic acid, and a decarboxylated and glucosylated derivative of DHCH, DHCH-Glc. Also, several amino acid conjugates of salicylic acid and benzoic acid were identified as end-products of the metabolism. Taking into account all the identified structures, it is possible to generalize the metabolism of Salicaceae adapted Notodontidae on their host plants (Schnurrer and Paetz 2023). Through adaptation, the larvae are able to play their host’s chemical defenses against each other. CQAs and salicortinoids are enzymatically deconstructed and rearranged to salicyloyl-, benzoyl- and mixed salicyloyl-benzoylquinic acids. Potentially toxic phase I intermediates end up glucosylated, sulfated or conjugated to glycine or alanine. We suggest that conjugation of plant defense metabolites with other plant defense metabolites may be a specific adaption of specialist lepidopterans to counteract the plant’s bouquet of defense compounds (Li et al. 2022). A similar adaption has been described for *M. sexta* (Heiling et al. 2022). Future research can show whether this strategy is present in other herbivore-host systems.

### Electronic Supplementary Material

Below is the link to the electronic supplementary material.

Supplementary Material 1
