# Supplementary material for: A Mechanism to Transform Complex Salicinoids with Caffeoylquinic Acids in Lepidopteran Specialist Herbivores (Notodontidae)
Source: J Chem Ecol. 2023 Nov 30;50(1-2):71–83. doi: 10.1007/s10886-023-01464-9 (PMC10991022; doi:10.1007/s10886-023-01464-9)
Supplement: Supplementary file 1 — Supplementary Material 1 [file 10886_2023_1464_MOESM1_ESM.docx]

**Supporting Information**

Contents

[1. Characterization of isolated ^13^C-labelled compounds 3](#_Toc149732983)

[1.1 Salicortin 3](#_Toc149732984)

[1.2 HCH-salicortin 5](#_Toc149732985)

[1.3 Tremulacin 7](#_Toc149732986)

[1.4 Trichocarpin 9](#_Toc149732987)

[1.1 Chlorogenic acid 15](#_Toc149732988)

[1.2 Neochlorogenic acid 20](#_Toc149732989)

[1.3 Chlorogenic acid methylester 26](#_Toc149732990)

[2. Identification of metabolites 31](#_Toc149732991)

[2.1 3-*O*-Benzoylquinic acid 32](#_Toc149732992)

[2.2 3,4-*O*-Bisbenzoylquinic acid 37](#_Toc149732993)

[2.3 3,5-*O*-Bisbenzoylquinic acid 42](#_Toc149732994)

[2.4 4,5-*O*-Bisbenzoylquinic acid 46](#_Toc149732995)

[2.5 2’,6’-*O*-Bisbenzoylsalicin 49](#_Toc149732996)

[2.6 Catechol glucoside 55](#_Toc149732997)

[2.7 Isosalicin 58](#_Toc149732998)

[2.8 Populin 61](#_Toc149732999)

[2.9 Nigracin 64](#_Toc149733000)

[2.10 3-*O*-glycosyl-2’-*O*-xylosyl-6’-*O*-rhamnosyl-quercetin 67](#_Toc149733001)

[2.11 Quercetin-3-*O*-sambubioside 72](#_Toc149733002)

[2.12 Rutin 77](#_Toc149733003)

[2.13 Quercetin-3-*O*-glucoside 80](#_Toc149733004)

[2.14 Quercetin-3-*O*-glucuronide 82](#_Toc149733005)

[2.15 Kaempferol-3-*O*-glucuronide 85](#_Toc149733006)

[3. Synthesis of reference compounds 89](#_Toc149733007)

[3.1 Caffeic acid-4-*O*-glucoside 89](#_Toc149733008)

[4. Reference compounds 92](#_Toc149733009)

[4.1 3-*O*-Salicyloyl quinic acid 92](#_Toc149733010)

[4.2 4-*O*-Salicyloyl quinic acid 92](#_Toc149733011)

[4.3 5-O-Salicyloyl-quinic acid 93](#_Toc149733012)

[4.4 4-*O*-Benzoyl quinic acid 93](#_Toc149733013)

[4.5 5-*O*-Benzoyl quinic acid 94](#_Toc149733014)

[4.6 3,4-*O*-bissalicyloyl quinic acid 94](#_Toc149733015)

[4.7 3,5-*O*-bissalicyloyl quinic acid 95](#_Toc149733016)

[4.8 4,5-*O*-bissalicyloyl quinic acid 95](#_Toc149733017)

[4.9 3-*O*-salicyloyl-4-*O*-benzoyl quinic acid 96](#_Toc149733018)

[4.10 3-*O*-salicyloyl-5-*O*-benzoyl quinic acid 96](#_Toc149733019)

[4.11 4-*O*-salicyloyl-5-*O*-benzoyl quinic acid 97](#_Toc149733020)

[4.12 Tremuloidin 97](#_Toc149733021)

[4.13 *ortho*-Hydroxyhippuric acid 98](#_Toc149733022)

[4.14 Salicin 98](#_Toc149733023)

[4.15 Salicylic acid 99](#_Toc149733024)

[5. ^13^C-Labelling of frass metabolites from feeding experiments 100](#_Toc149733025)

[5.1 [U-^13^C]Caffeoylquinic acid experiments 100](#_Toc149733026)

[5.2 [U-^13^C]Salicortinoid experiments 109](#_Toc149733027)

[5.3 [U-^13^C]Salicortinoid labelling pattern vs. [U-^13^C]caffeoylquinic acid labelling pattern 141](#_Toc149733028)

[5.4 [U-^13^C]Trichocarpin experiment 143](#_Toc149733029)

[6. Metabolism of [U-^13^C]salicortinoids and [U-^13^C]trichocarpin 151](#_Toc149733030)

[7. Stability of salicortinoids on *P. nigra* leaf surfaces 154](#_Toc149733031)

[8. References 155](#_Toc149733032)

# Characterization of isolated ^13^C-labelled compounds

## 1.1 Salicortin

**
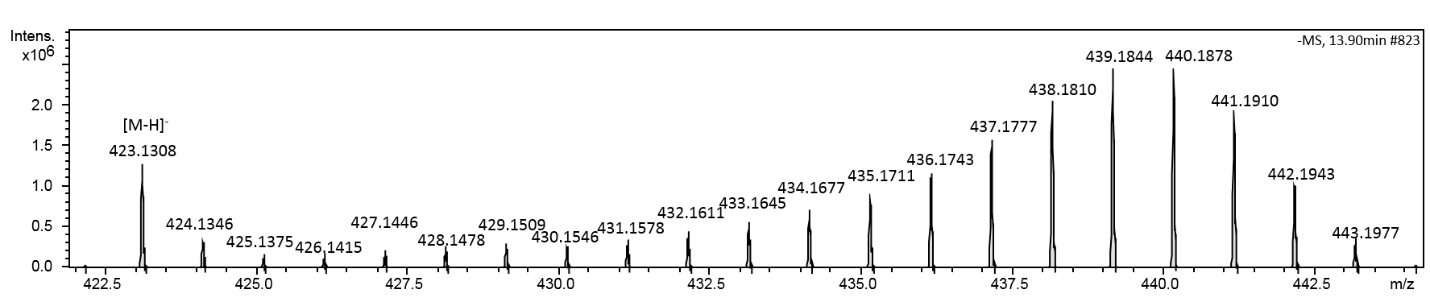
**

Figure 1: [U-^13^C]salicortin, HRESIMS spectrum, m/z 423.1308 [M-H]^-^

Table 1: Extracted MS data used calculation of the ^13^C-enrichment of salicortin isotopologues (m/z) together with their signal intensity

| C-isotope | | salicortin | |
| --- | --- | --- | --- |
| ^12^C | ^13^C | *m/z* | intensity |
| 20 | 0 | 423.1308 | 1184950 |
| 19 | 1 | 424.1346 | 298384 |
| 18 | 2 | 425.1375 | 112160 |
| 17 | 3 | 426.1415 | 107298 |
| 16 | 4 | 427.1446 | 146726 |
| 15 | 5 | 428.1478 | 192920 |
| 14 | 6 | 429.1509 | 219615 |
| 13 | 7 | 430.1546 | 236412 |
| 12 | 8 | 431.1578 | 278842 |
| 11 | 9 | 432.1611 | 368175 |
| 10 | 10 | 433.1645 | 486659 |
| 9 | 11 | 434.1677 | 637158 |
| 8 | 12 | 435.1711 | 824672 |
| 7 | 13 | 436.1743 | 1099542 |
| 6 | 14 | 437.1777 | 1485717 |
| 5 | 15 | 438.181 | 1966451 |
| 4 | 16 | 439.1844 | 2356812 |
| 3 | 17 | 440.1878 | 2364725 |
| 2 | 18 | 441.191 | 1855908 |
| 1 | 19 | 442.1943 | 989747 |
| 0 | 20 | 443.1977 | 282746 |
| ^13^C-incorporation | 82% | R | 0.89 |

Table 2: Extracted MS data used for calculation of the ^13^C-enrichment of salicortin isotopologues (m/z) together with their signal intensities

|  | HCH | | | saligenin | | | salicin | | |
| --- | --- | --- | --- | --- | --- | --- | --- | --- | --- |
|  |  | | |  | | |  | | |
|  | 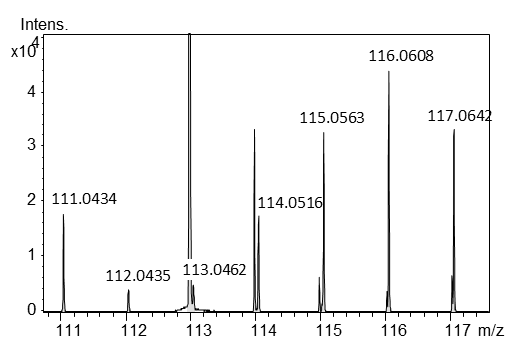 | | | 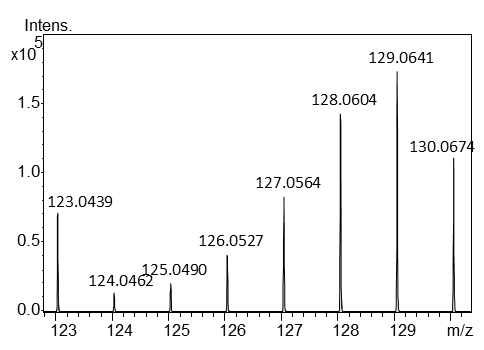 | | | 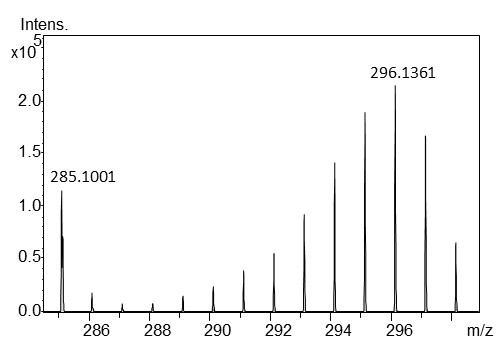 | | |
| ^13^C | *m/z* | intensity |  | *m/z* | intensity |  | *m/z* | intensity |  |
| 0 | 111.0434 | 17670 |  | 123.0439 | 71949 |  | 285.1001 | 113555 |  |
| 1 | 112.0435 | 4160 |  | 124.0462 | 14520 |  | 286.1028 | 19038 |  |
| 2 | 113.0462 | 5096 |  | 125.049 | 20812 |  | 287.1059 | 8021 |  |
| 3 | 114.0516 | 17488 |  | 126.0527 | 41381 |  | 288.1094 | 8712 |  |
| 4 | 115.0563 | 32485 |  | 127.0564 | 82152 |  | 289.1128 | 16022 |  |
| 5 | 116.0608 | 43532 |  | 128.0604 | 141665 |  | 290.1159 | 24415 |  |
| 6 | 117.0642 | 33088 |  | 129.0641 | 172276 |  | 291.1198 | 38404 |  |
| 7 |  |  |  | 130.0674 | 111147 |  | 292.123 | 55766 |  |
| 8 |  |  |  |  |  |  | 293.1263 | 91964 |  |
| 9 |  |  |  |  |  |  | 294.1296 | 140405 |  |
| 10 |  |  |  |  |  |  | 295.1329 | 187819 |  |
| 11 |  |  |  |  |  |  | 296.1361 | 212391 |  |
| 12 |  |  |  |  |  |  | 297.1397 | 165669 |  |
| 13 |  |  |  |  |  |  | 298.1428 | 66245 |  |

**
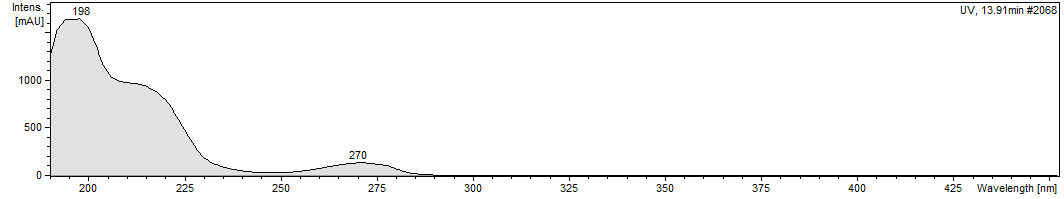
**

Figure 2: [U-^13^C]salicortin, UV spectrum from HPLC-DAD

## 1.2 HCH-salicortin

**
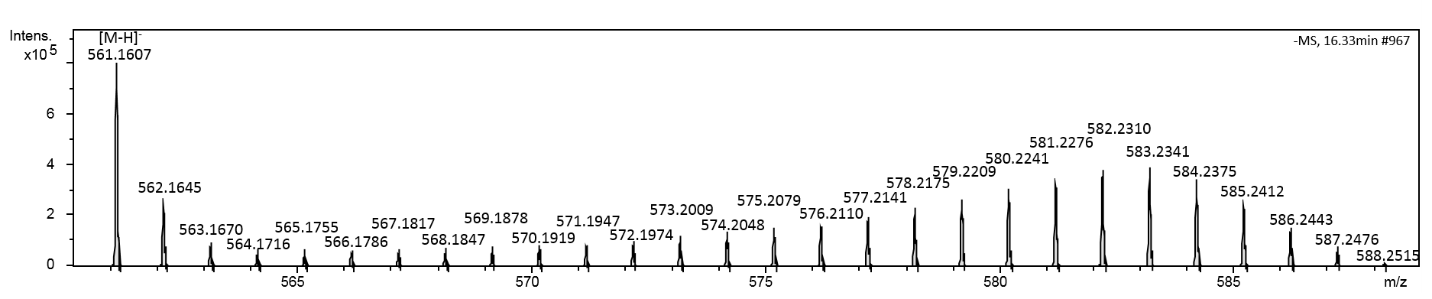
**

Figure 3: [U-^13^C]HCH-salicortin, HRESIMS spectrum, m/z 561.1607 [M-H]^-^

Table 3: Extracted MS data used for calculation of the ^13^C-enrichment of HCH-salicortin isotopologues (m/z) together with their signal intensities

| C-isotope | | HCH-Salicortin | |
| --- | --- | --- | --- |
| ^12^C | ^13^C | *m/z* | intensity |
| 27 | 0 | 561.1607 | 774453 |
| 26 | 1 | 562.1645 | 243323 |
| 25 | 2 | 563.167 | 77277 |
| 24 | 3 | 564.1716 | 43272 |
| 23 | 4 | 565.1755 | 43993 |
| 22 | 5 | 566.1786 | 47917 |
| 21 | 6 | 567.1817 | 49430 |
| 20 | 7 | 568.1847 | 50641 |
| 19 | 8 | 569.1878 | 54230 |
| 18 | 9 | 570.1919 | 61433 |
| 17 | 10 | 571.1947 | 73716 |
| 16 | 11 | 572.1974 | 83366 |
| 15 | 12 | 573.2009 | 95826 |
| 14 | 13 | 574.2048 | 113035 |
| 13 | 14 | 575.2079 | 128495 |
| 12 | 15 | 576.211 | 151818 |
| 11 | 16 | 577.2141 | 175666 |
| 10 | 17 | 578.2175 | 206148 |
| 9 | 18 | 579.2209 | 240225 |
| 8 | 19 | 580.2241 | 281612 |
| 7 | 20 | 581.2276 | 322801 |
| 6 | 21 | 582.231 | 355486 |
| 5 | 22 | 583.2341 | 364493 |
| 4 | 23 | 584.2375 | 320482 |
| 3 | 24 | 585.2412 | 237219 |
| 2 | 25 | 586.2443 | 135629 |
| 1 | 26 | 587.2476 | 53732 |
| 0 | 27 | 588.2515 | 11588 |
| ^13^C-incorporation | 77% | R | 0.80 |

Table 4: Extracted MS data used for calculation of the ^13^C-enrichment of HCH-salicortin isotopologues (m/z) together with their signal intensities

|  | HCH | | | saligenin | | | salicin | | |
| --- | --- | --- | --- | --- | --- | --- | --- | --- | --- |
|  |  | | |  | | |  | | |
|  | 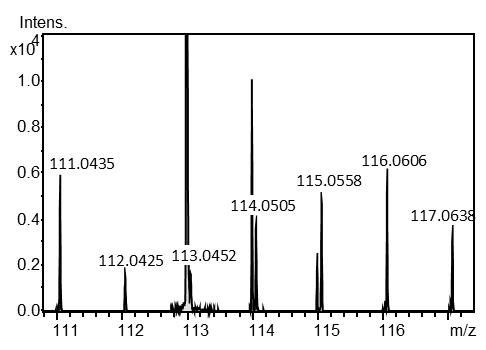 | | | 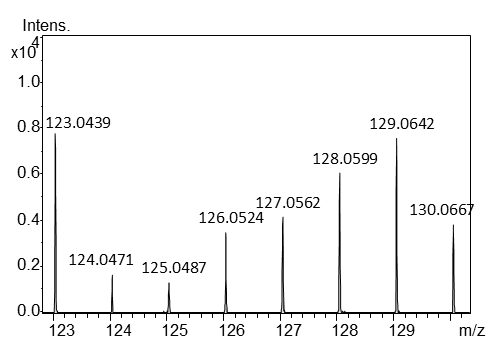 | | | 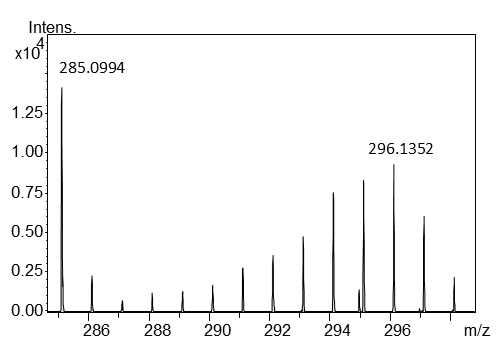 | | |
| ^13^C | *m/z* | intensity |  | *m/z* | intensity |  | *m/z* | intensity |  |
| 0 | 111.0435 | 5580 |  | 123.0439 | 7745 |  | 285.0994 | 14128 |  |
| 1 | 112.0425 | 1587 |  | 124.0471 | 1653 |  | 286.1035 | 2344 |  |
| 2 | 113.0452 | 1656 |  | 125.0487 | 1340 |  | 287.105 | 758 |  |
| 3 | 114.0505 | 3847 |  | 126.0524 | 3490 |  | 288.1058 | 1208 |  |
| 4 | 115.0558 | 4854 |  | 127.0562 | 4156 |  | 289.1137 | 1372 |  |
| 5 | 116.0606 | 5862 |  | 128.0599 | 6043 |  | 290.1144 | 1686 |  |
| 6 | 117.0638 | 3407 |  | 129.0642 | 7579 |  | 291.12 | 2844 |  |
| 7 |  |  |  | 130.0667 | 3857 |  | 292.1205 | 3630 |  |
| 8 |  |  |  |  |  |  | 293.1261 | 4791 |  |
| 9 |  |  |  |  |  |  | 294.1283 | 7473 |  |
| 10 |  |  |  |  |  |  | 295.1322 | 8276 |  |
| 11 |  |  |  |  |  |  | 296.1352 | 9278 |  |
| 12 |  |  |  |  |  |  | 297.1397 | 6052 |  |
| 13 |  |  |  |  |  |  | 298.1418 | 2177 |  |

**
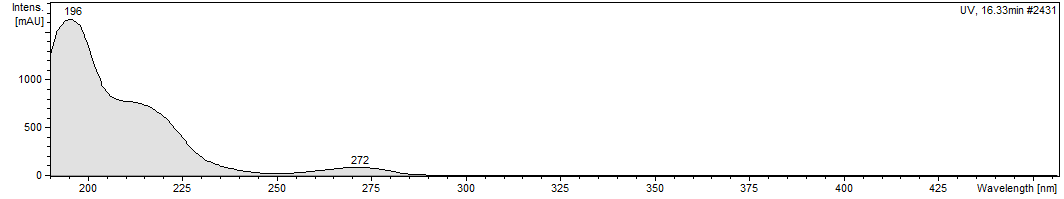
**

Figure 4: [U-^13^C]HCH-salicortin, UV spectrum from HPLC-DAD

## 1.3 Tremulacin

**
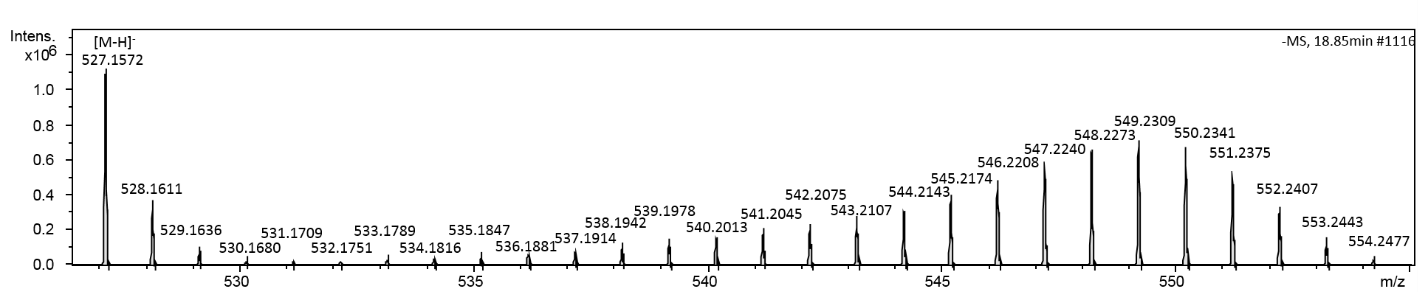
**

Figure 5: [U-^13^C]tremulacin, HRESIMS spectrum, m/z 527.1572 [M-H]^-^

Table 5: Extracted MS data used for calculation of the ^13^C-enrichment of tremulacin isotopologues (m/z) together with their signal intensities

| C-isotope | | tremulacin | |
| --- | --- | --- | --- |
| ^12^C | ^13^C | *m/z* | intensity |
| 27 | 0 | 527.1572 | 1084012 |
| 26 | 1 | 528.1611 | 334271 |
| 25 | 2 | 529.1636 | 74387 |
| 24 | 3 | 530.168 | 20544 |
| 23 | 4 | 531.1709 | 16615 |
| 22 | 5 | 532.1751 | 20992 |
| 21 | 6 | 533.1789 | 27081 |
| 20 | 7 | 534.1816 | 36024 |
| 19 | 8 | 535.1847 | 43478 |
| 18 | 9 | 536.1881 | 60086 |
| 17 | 10 | 537.1914 | 75651 |
| 16 | 11 | 538.1942 | 93536 |
| 15 | 12 | 539.1978 | 117852 |
| 14 | 13 | 540.2013 | 144384 |
| 13 | 14 | 541.2045 | 174515 |
| 12 | 15 | 542.2075 | 204382 |
| 11 | 16 | 543.2107 | 249434 |
| 10 | 17 | 544.2143 | 302076 |
| 9 | 18 | 545.2174 | 368267 |
| 8 | 19 | 546.2208 | 450671 |
| 7 | 20 | 547.224 | 553231 |
| 6 | 21 | 548.2273 | 636423 |
| 5 | 22 | 549.2309 | 680613 |
| 4 | 23 | 550.2341 | 642119 |
| 3 | 24 | 551.2375 | 504593 |
| 2 | 25 | 552.2407 | 296275 |
| 1 | 26 | 553.2443 | 121343 |
| 0 | 27 | 554.2477 | 28925 |
| ^13^C-incorporation | 79% | R | 0.87 |

Table 6: Extracted MS data used for calculation of the ^13^C-enrichment of salicortin isotopologues (m/z) together with their signal intensities

|  | HCH | | | benzoic acid | | | salicin | | |
| --- | --- | --- | --- | --- | --- | --- | --- | --- | --- |
|  |  | | |  | | |  | | |
|  | 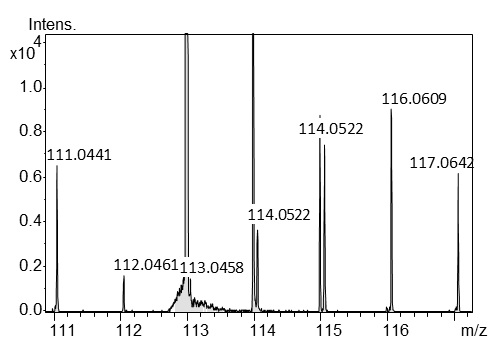 | | | 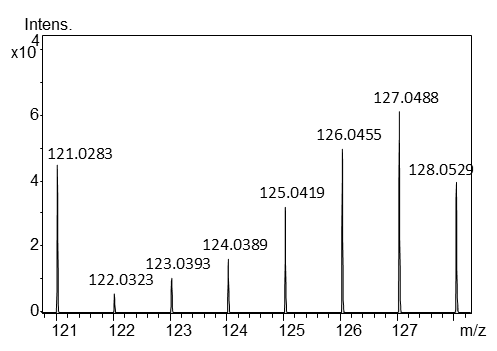 | | | 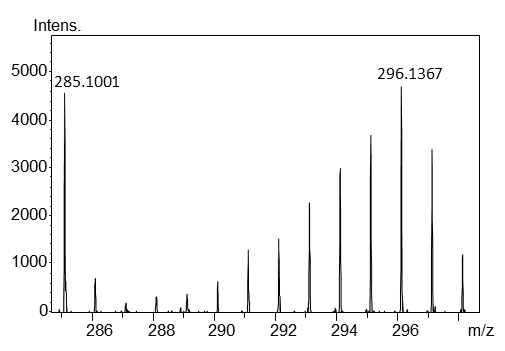 | | |
| ^13^C | *m/z* | intensity |  | *m/z* | intensity |  | *m/z* | intensity |  |
| 0 | 111.0441 | 6508 |  | 121.0276 | 49042 |  | 285.1001 | 4538 |  |
| 1 | 112.0461 | 1625 |  | 122.0309 | 7345 |  | 286.1006 | 740 |  |
| 2 | 113.0458 | 1464 |  | 123.0369 | 10352 |  | 287.1026 | 205 |  |
| 3 | 114.0522 | 3663 |  | 124.038 | 17250 |  | 288.1075 | 342 |  |
| 4 | 115.0577 | 7368 |  | 125.0411 | 33326 |  | 289.1098 | 409 |  |
| 5 | 116.0609 | 8990 |  | 126.0446 | 53262 |  | 290.1147 | 643 |  |
| 6 | 117.0642 | 6139 |  | 127.048 | 62892 |  | 291.1189 | 1296 |  |
| 7 |  |  |  | 128.0518 | 39934 |  | 292.1213 | 1531 |  |
| 8 |  |  |  |  |  |  | 293.1258 | 2295 |  |
| 9 |  |  |  |  |  |  | 294.1289 | 2988 |  |
| 10 |  |  |  |  |  |  | 295.1345 | 3683 |  |
| 11 |  |  |  |  |  |  | 296.1367 | 4672 |  |
| 12 |  |  |  |  |  |  | 297.1411 | 3390 |  |
| 13 |  |  |  |  |  |  | 298.1437 | 1228 |  |

**
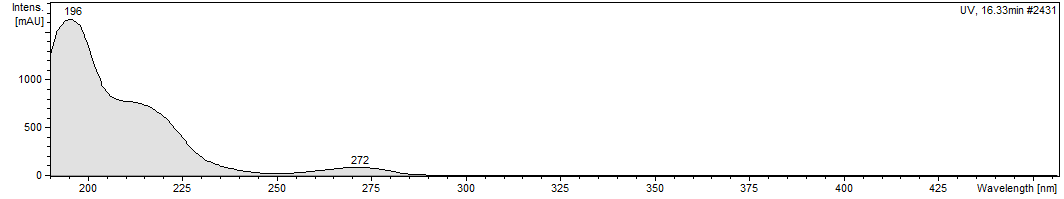
**

Figure 6: [U-^13^C]tremulacin, UV spectrum from HPLC-DAD

## 1.4 Trichocarpin

**
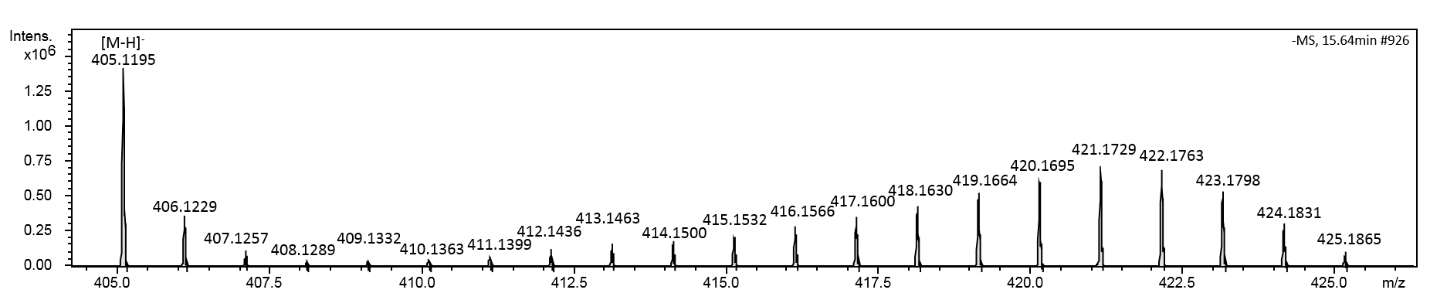
**

Figure 7: [U-^13^C]trichocarpin, HRESIMS spectrum, m/z 405.1195 [M-H]^-^

Table 7: Extracted MS data used for calculation of the ^13^C-enrichment of trichocarpin isotopologues (m/z) together with their signal intensities

| C-isotope | | trichocarpin | |
| --- | --- | --- | --- |
| ^12^C | ^13^C | *m/z* | intensity |
| 20 | 0 | 405.1195 | 1362989 |
| 19 | 1 | 406.1229 | 316324 |
| 18 | 2 | 407.1257 | 68543 |
| 17 | 3 | 408.1289 | 28719 |
| 16 | 4 | 409.1332 | 32262 |
| 15 | 5 | 410.1363 | 46041 |
| 14 | 6 | 411.1399 | 62307 |
| 13 | 7 | 412.1436 | 85040 |
| 12 | 8 | 413.1463 | 117643 |
| 11 | 9 | 414.15 | 145270 |
| 10 | 10 | 415.1532 | 191706 |
| 9 | 11 | 416.1566 | 242061 |
| 8 | 12 | 417.16 | 307696 |
| 7 | 13 | 418.163 | 384752 |
| 6 | 14 | 419.1664 | 476353 |
| 5 | 15 | 420.1695 | 591812 |
| 4 | 16 | 421.1729 | 668498 |
| 3 | 17 | 422.1763 | 639984 |
| 2 | 18 | 423.1798 | 487280 |
| 1 | 19 | 424.1831 | 257159 |
| 0 | 20 | 425.1865 | 68943 |
| ^13^C-incorporation | 79% | R | 0.90 |

Table 8: Extracted MS data used for calculation of the ^13^C-enrichment of trichocarpin isotopologues (m/z) together with their signal intensities

|  | gentisic acid glucoside lactone | | | Gentisic acid | | | Benzyl gentisate | | |
| --- | --- | --- | --- | --- | --- | --- | --- | --- | --- |
|  |  | | |  | | |  | | |
|  | 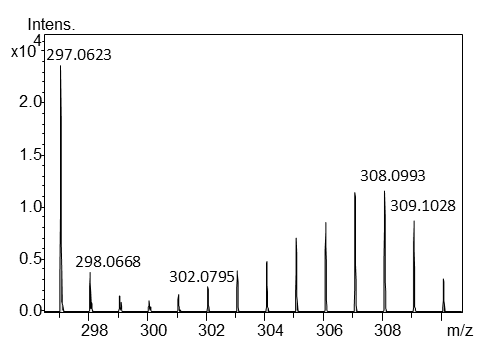 | | | 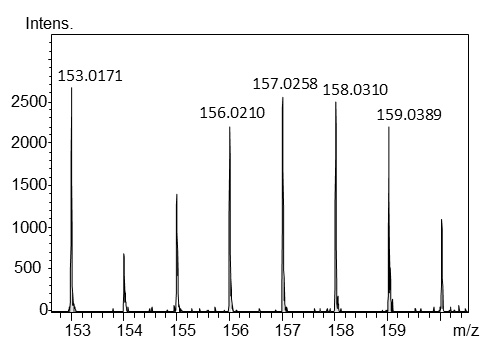 | | | 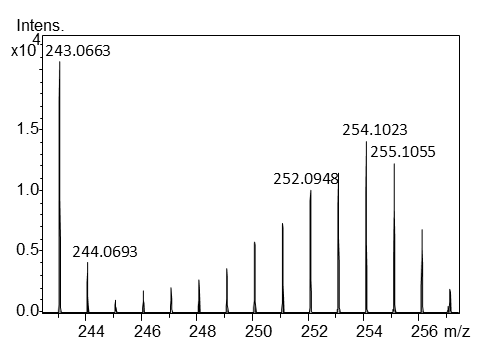 | | |
| ^13^C | *m/z* | intensity |  | *m/z* | intensity |  | *m/z* | intensity |  |
| 0 | 297.0623 | 23544 |  | 153.0171 | 2668 |  | 243.0663 | 20638 |  |
| 1 | 298.0668 | 3791 |  | 154.0157 | 695 |  | 244.0693 | 4156 |  |
| 2 | 299.0694 | 1623 |  | 155.018 | 1402 |  | 245.071 | 1130 |  |
| 3 | 300.0721 | 1134 |  | 156.021 | 2204 |  | 246.076 | 1831 |  |
| 4 | 301.0746 | 1677 |  | 157.0258 | 2552 |  | 247.0789 | 2054 |  |
| 5 | 302.0795 | 2469 |  | 158.031 | 2497 |  | 248.0817 | 2734 |  |
| 6 | 303.0834 | 4011 |  | 159.0389 | 2192 |  | 249.0852 | 3645 |  |
| 7 | 304.0868 | 4906 |  | 160.0439 | 1094 |  | 250.0883 | 5775 |  |
| 8 | 305.0881 | 7144 |  |  |  |  | 251.0918 | 7354 |  |
| 9 | 306.0928 | 8549 |  |  |  |  | 252.0948 | 10021 |  |
| 10 | 307.0952 | 11462 |  |  |  |  | 253.0993 | 11540 |  |
| 11 | 308.0993 | 11552 |  |  |  |  | 254.1023 | 14106 |  |
| 12 | 309.1028 | 8730 |  |  |  |  | 255.1055 | 12303 |  |
| 13 | 310.1058 | 3198 |  |  |  |  | 256.1097 | 6850 |  |
| 14 |  |  |  |  |  |  | 257.1145 | 2015 |  |

Figure 8: Trichocarpin, structure with chemical shifts (MeOH-d_3_), the acquired analytical data is in accordance with previous literature (Fedorova et al. 2020)

**
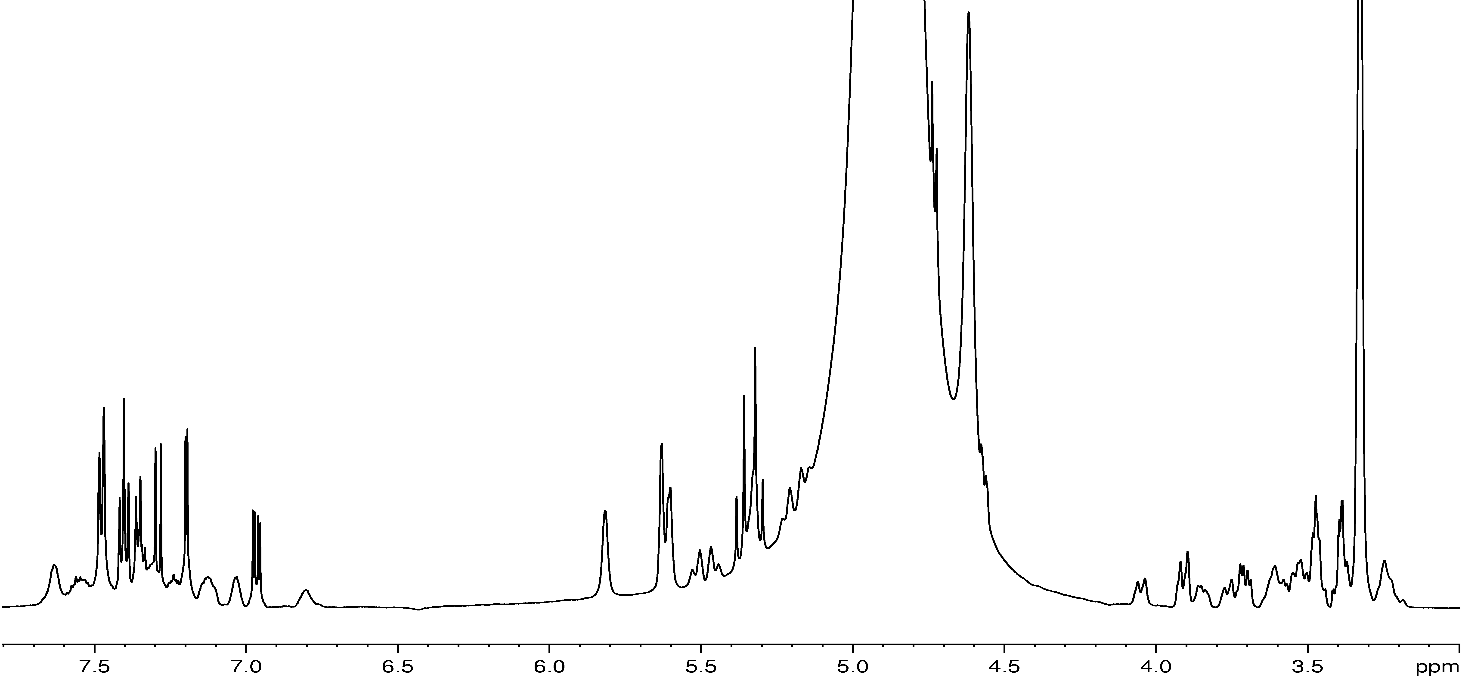
**

Figure 9: [U-^13^C]trichocarpin, ­^1^H-NMR spectrum (MeOH-d_3_)


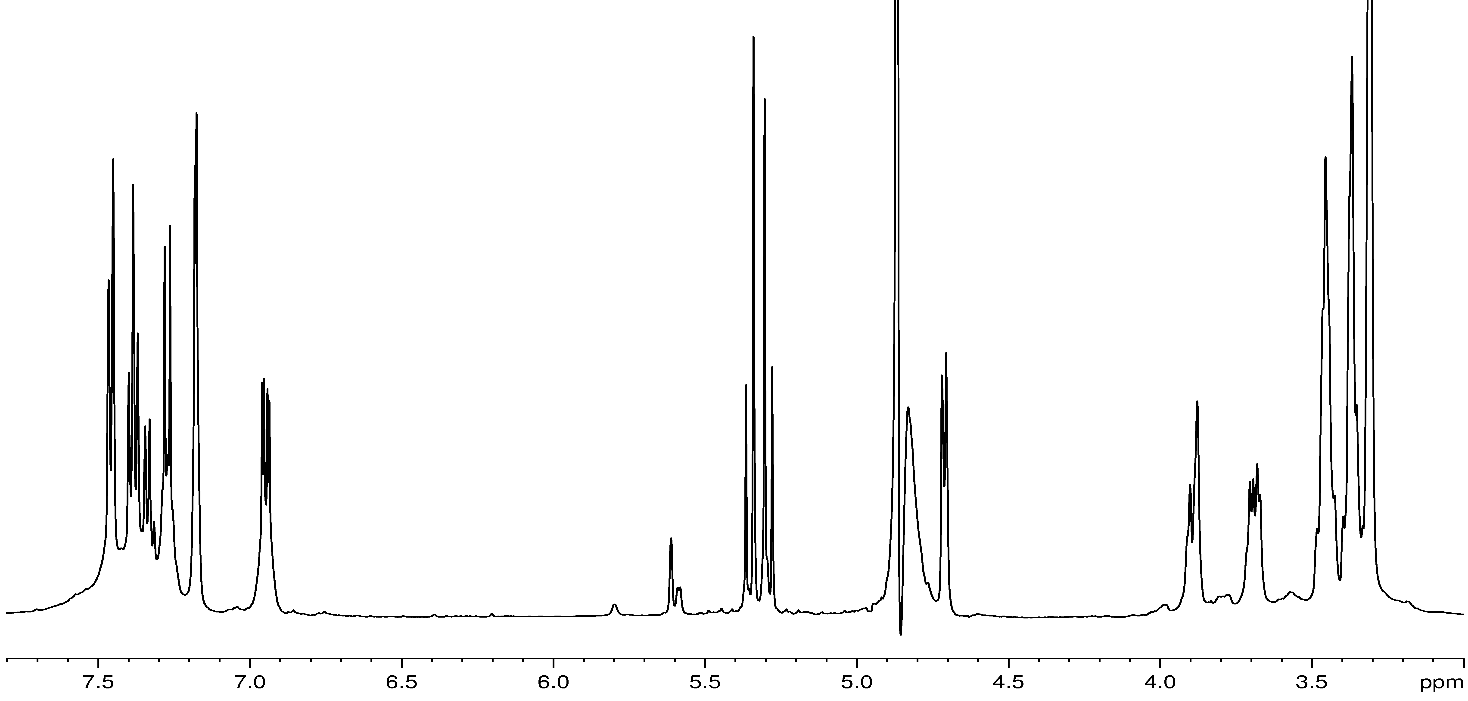


Figure 10: [U-^13^C]trichocarpin, ­^1^H-NMR spectrum, ^13^C decoupled (MeOH-d_3_)

**
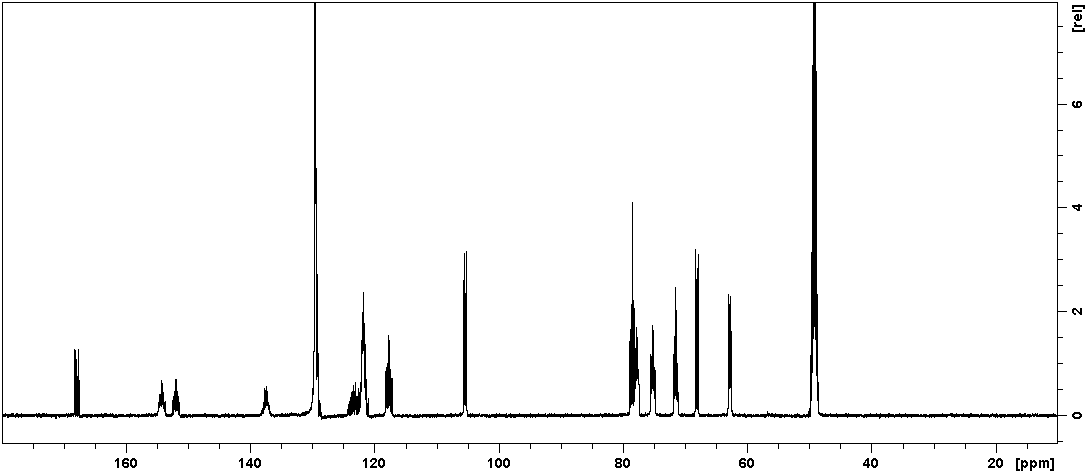
**

Figure 11: [U-^13^C]trichocarpin, ^13^C-NMR spectrum (MeOH-d_3_)

**
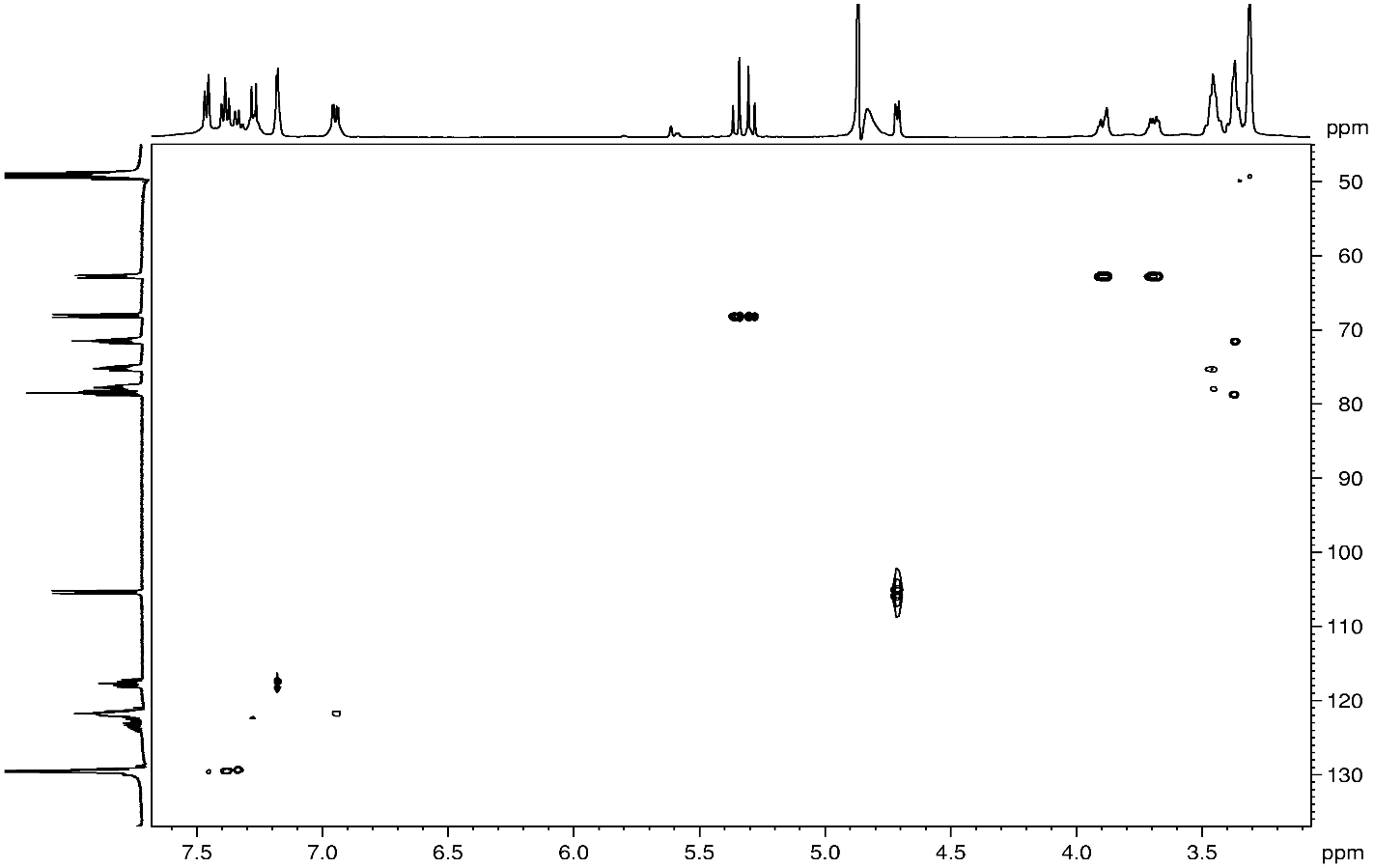
**

Figure 12: [U-^13^C]trichocarpin, HSQC spectrum (MeOH-d_3_)


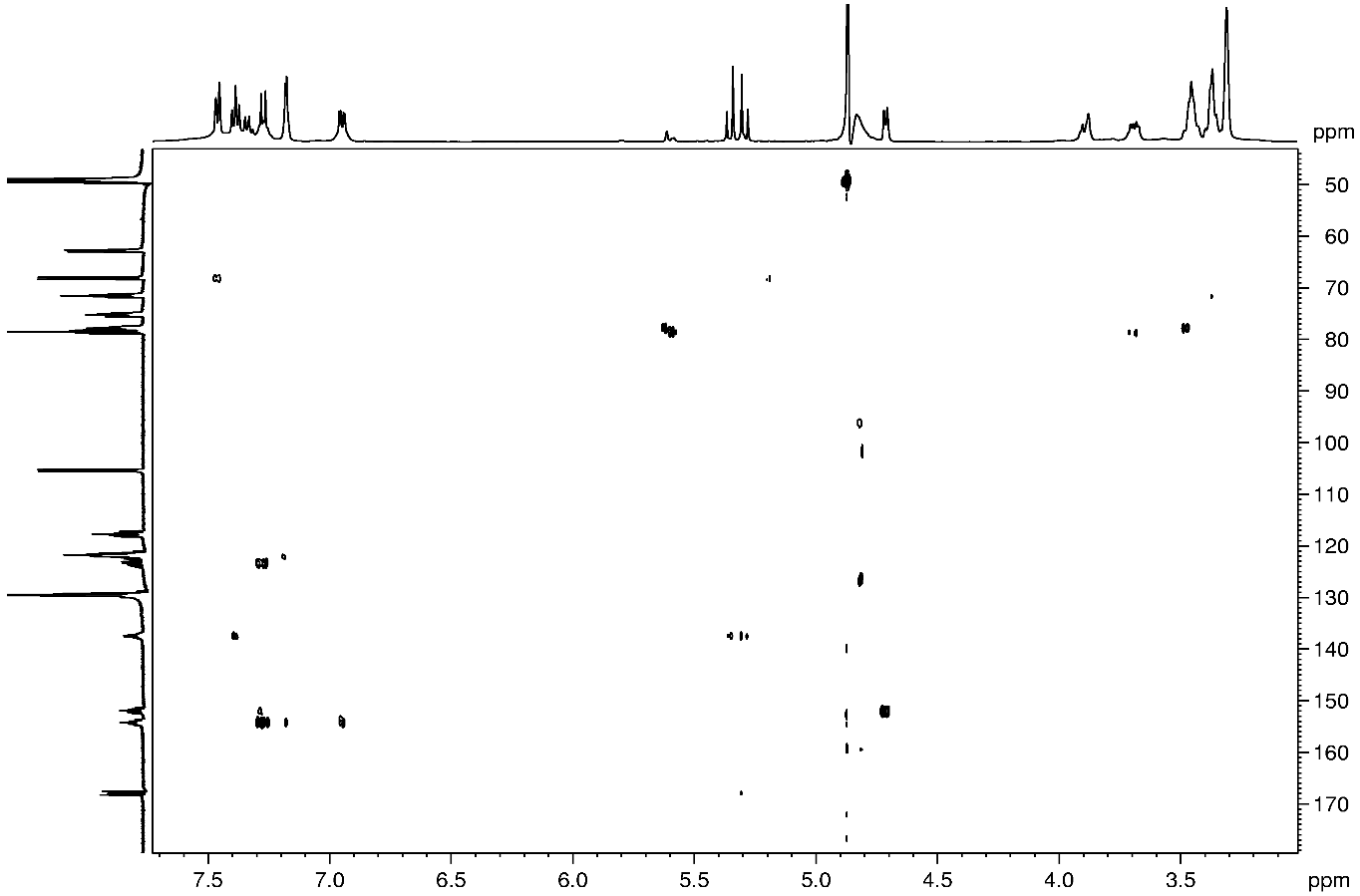


Figure 13: [U-^13^C]trichocarpin, HMBC spectrum (MeOH-d_3_)


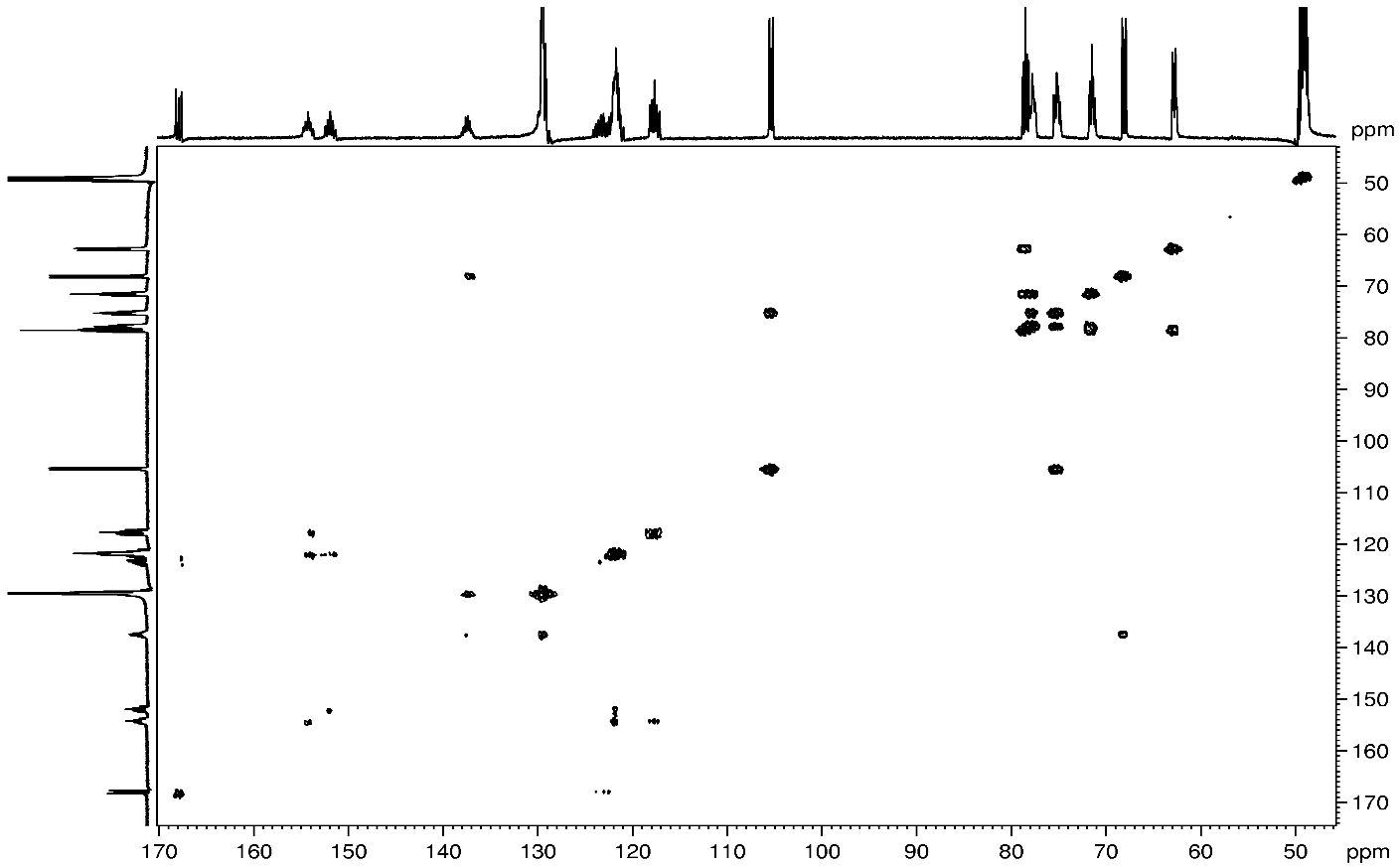


Figure 14: [U-^13^C]trichocarpin, ^13^C-^13^C COSY spectrum (MeOH-d_3_)


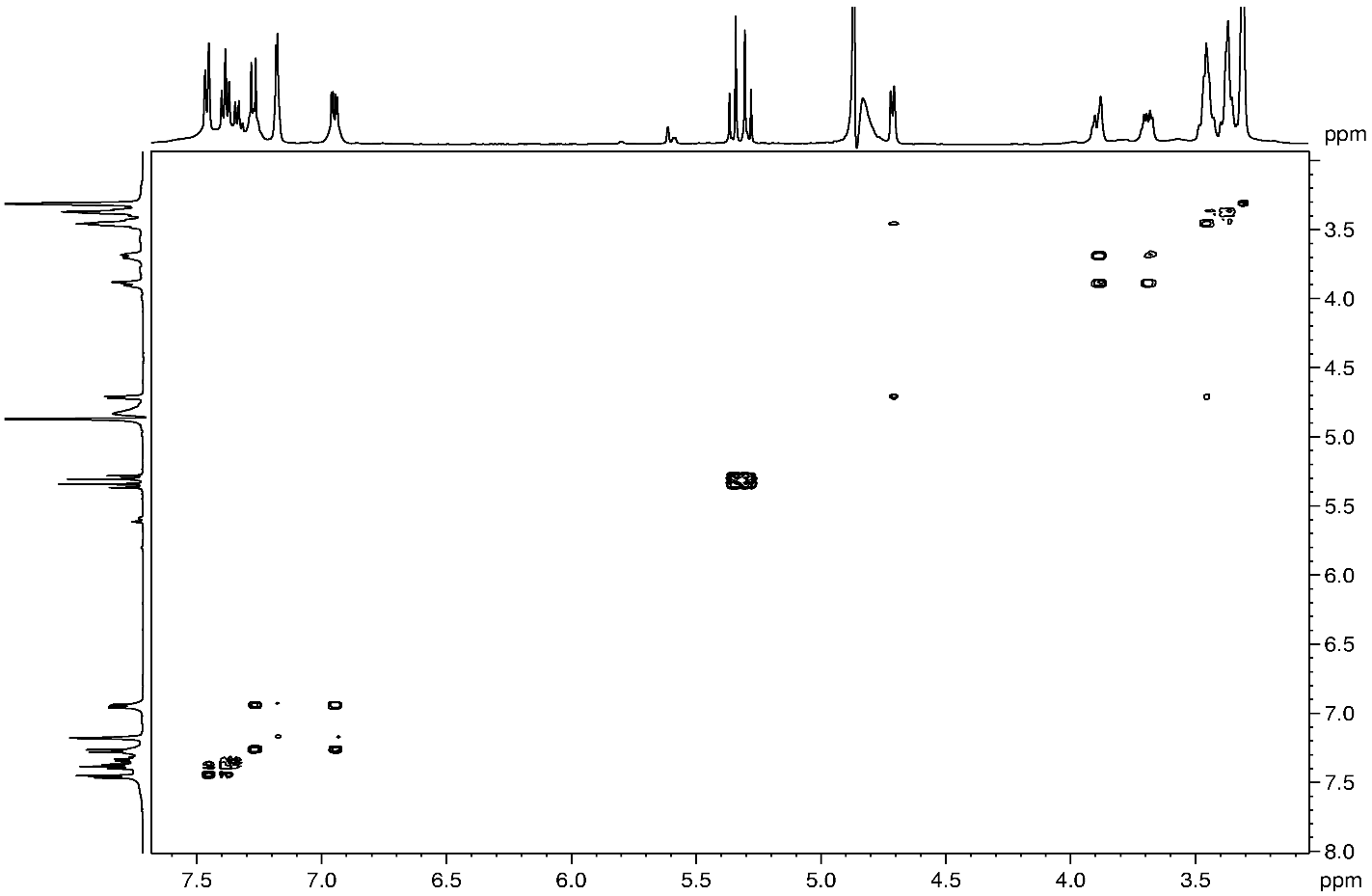


Figure 15: [U-^13^C]trichocarpin, ^1^H-^1^H COSY spectrum (MeOH-d_3_)

**
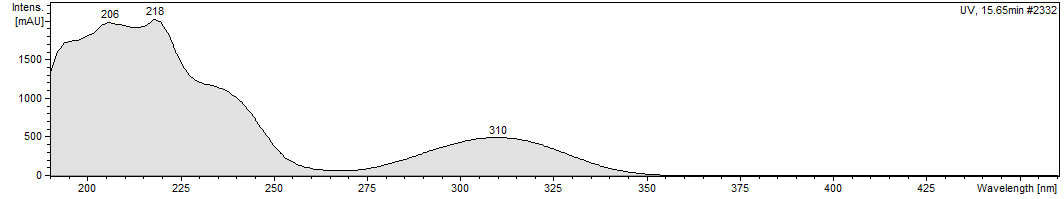
**

Figure 16: [U-^13^C]trichocarpin, UV spectrum from HPLC-DAD

## 1.1 Chlorogenic acid


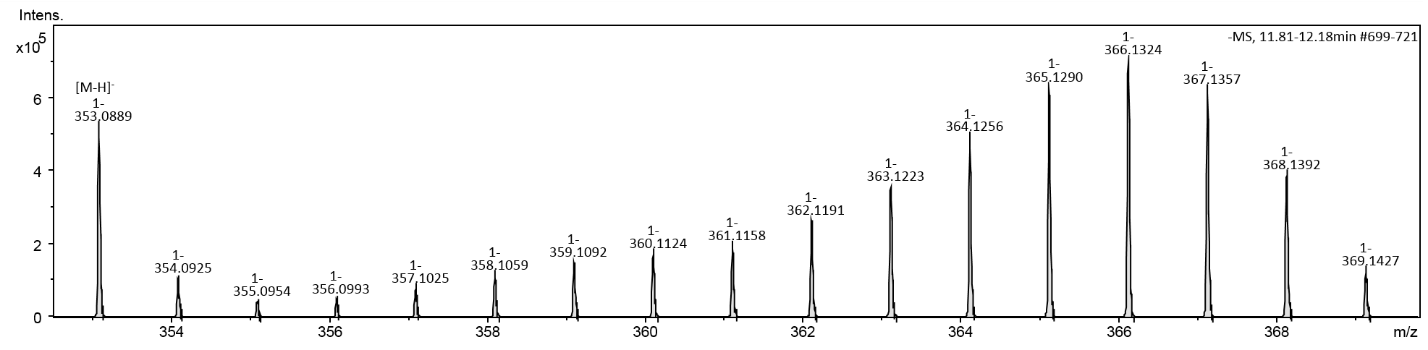


Figure 17: chlorogenic acid, HRESIMS spectrum, m/z 353.0889 [M-H]^-^

Table 9: Extracted MS data used for the calculation of the ^13^C-enrichment of chlorogenic acid isotopologues (m/z) together with their signal intensity

| C-isotope | | chlorogenic acid | |
| --- | --- | --- | --- |
| ^12^C | ^13^C | m/z | intensity |
| 16 | 0 | 353.0889 | 520919 |
| 15 | 1 | 354.0925 | 105743 |
| 14 | 2 | 355.0954 | 42970 |
| 13 | 3 | 356.0993 | 48757 |
| 12 | 4 | 357.1025 | 76898 |
| 11 | 5 | 358.1059 | 113999 |
| 10 | 6 | 359.1092 | 150087 |
| 9 | 7 | 360.1124 | 172920 |
| 8 | 8 | 361.1158 | 194064 |
| 7 | 9 | 362.1191 | 262715 |
| 6 | 10 | 363.1223 | 354189 |
| 5 | 11 | 364.1256 | 490132 |
| 4 | 12 | 365.129 | 626407 |
| 3 | 13 | 366.1324 | 698211 |
| 2 | 14 | 367.1357 | 620646 |
| 1 | 15 | 368.1392 | 387317 |
| 0 | 16 | 369.1427 | 125224 |
| ^13^C-incorporation | 77% | R | 0.92 |

Table 10: Extracted MS data which were used for the calculation of the ^13^C-enrichment of the chlorogenic acid isotopologues (m/z) together with their signal intensity

|  | quinic acid | | | Caffeic acid | | |
| --- | --- | --- | --- | --- | --- | --- |
|  |  | | |  | | |
|  | 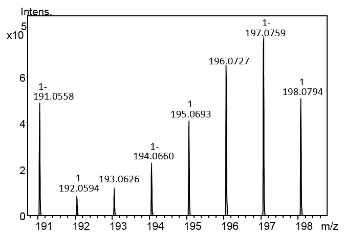 | | | 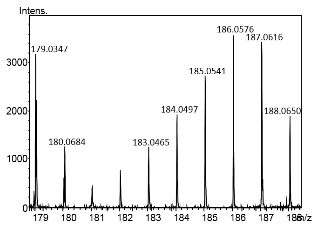 | | |
| ^13^C | *m/z* | intensity |  | *m/z* | intensity |  |
| 0 | 191.0558 | 469631 |  | 179.0347 | 3089 |  |
| 1 | 192.0594 | 75500 |  | 180.035 | 536 |  |
| 2 | 193.0626 | 103149 |  | 181.0408 | 419 |  |
| 3 | 194.066 | 211343 |  | 182.0424 | 692 |  |
| 4 | 195.0693 | 390907 |  | 183.0465 | 1165 |  |
| 5 | 196.0727 | 631164 |  | 184.0497 | 1839 |  |
| 6 | 197.0759 | 754348 |  | 185.0541 | 2641 |  |
| 7 |  |  |  | 186.0576 | 3480 |  |
| 8 |  |  |  | 187.0616 | 3344 |  |
| 9 |  |  |  | 188.065 | 1809 |  |

Figure 18: Chlorogenic acid, structure with chemical shifts (MeOH-d_3_), the acquired analytical data is in accordance with previous literature (Choi et al. 2006; Iwai et al. 2004)


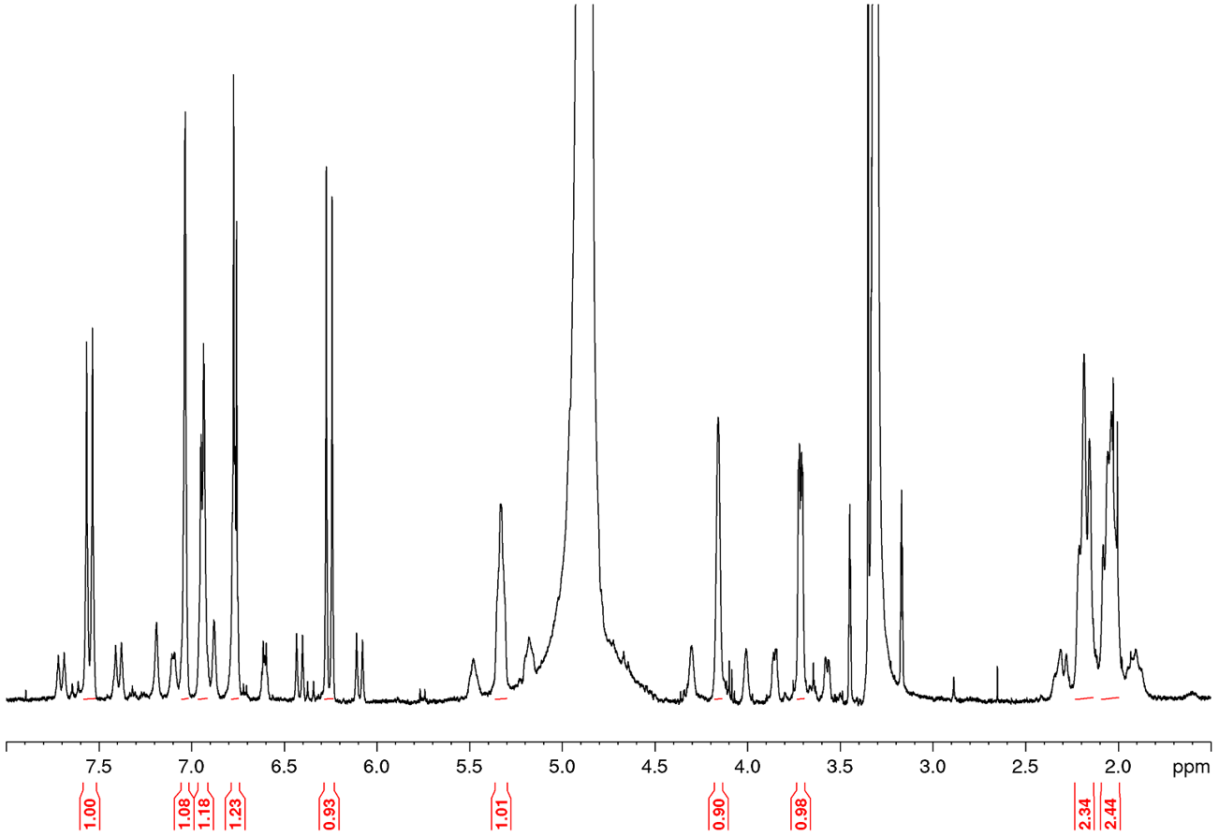


Figure 19: [U-^13^C]chlorogenic acid, ­^1^H-NMR spectrum (MeOH-d_3_)


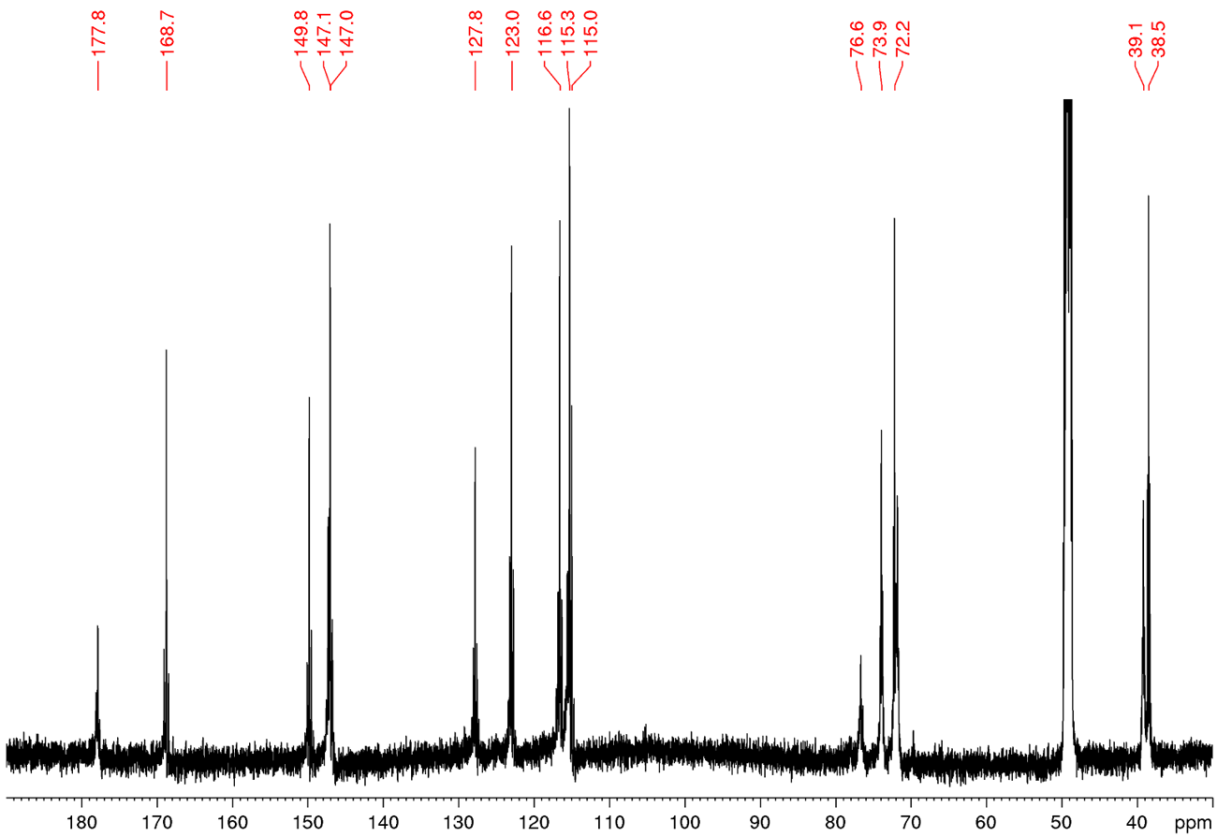


Figure 20: [U-^13^C]chlorogenic acid, ^13^C-NMR spectrum (MeOH-d_3_)


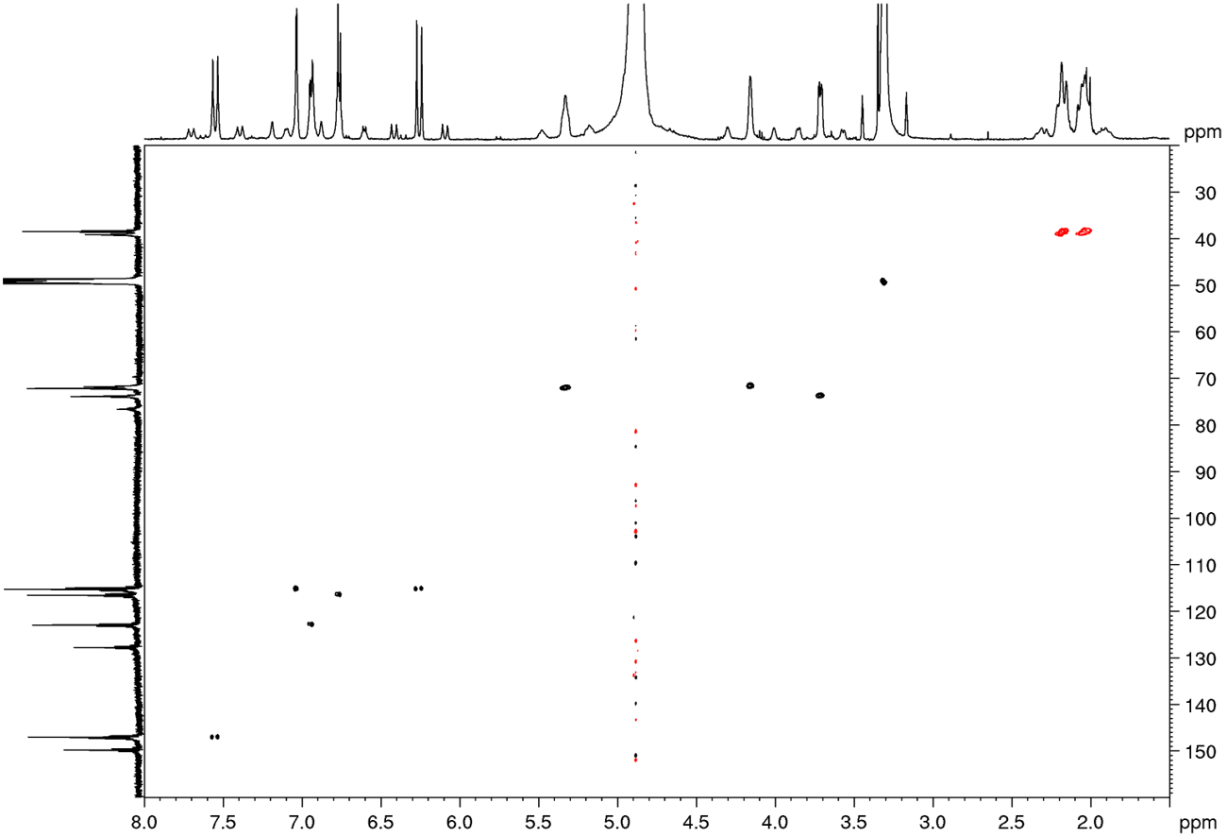


Figure 21: [U-^13^C]chlorogenic acid, HSQC spectrum (MeOH-d_3_)


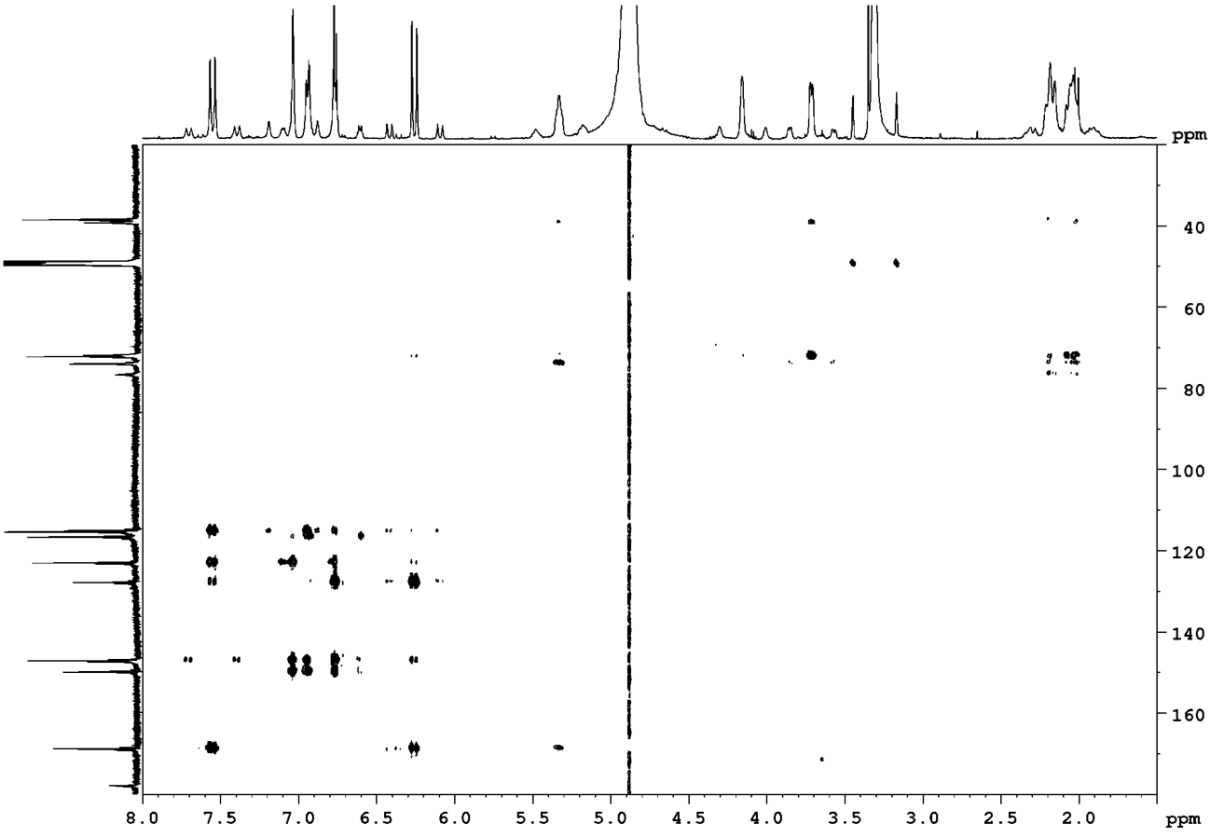


Figure 22: [U-^13^C]chlorogenic acid, HMBC spectrum (MeOH-d_3_)


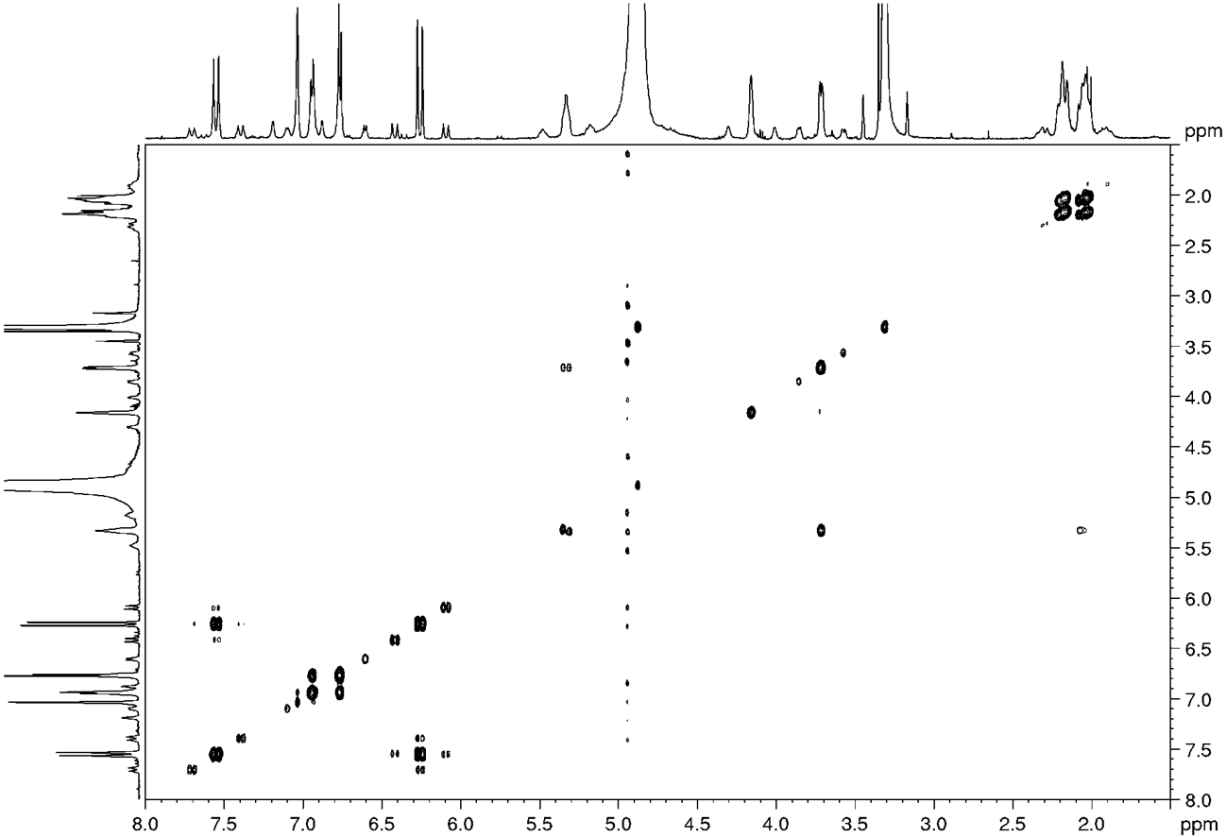


Figure 23: [U-^13^C]chlorogenic acid, ^1^H-^1^H COSY spectrum (MeOH-d_3_)

## 1.2 Neochlorogenic acid


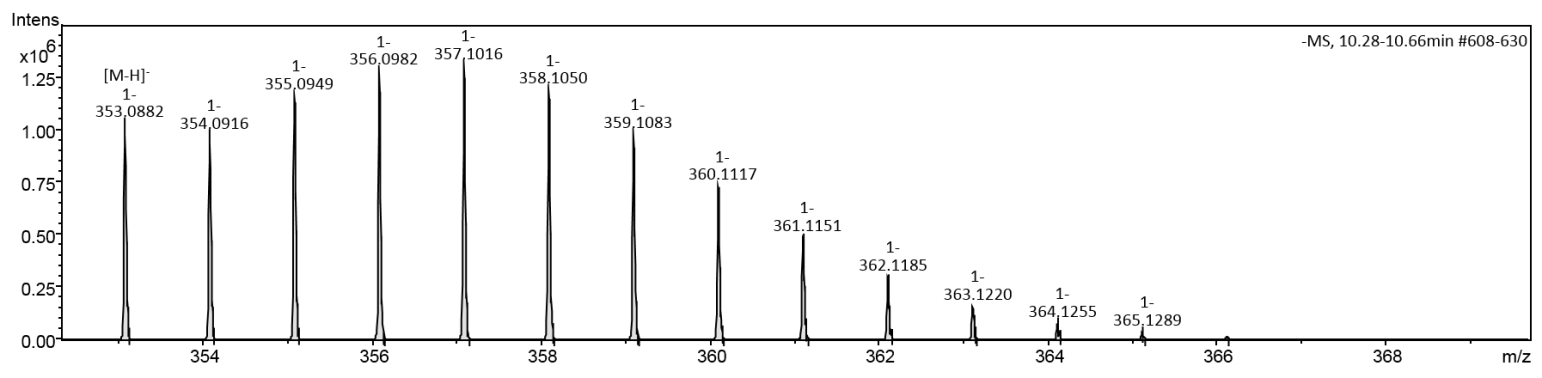


Figure 24: Neochlorogenic acid, HRESIMS spectrum, m/z 353.0889 [M-H]^-^

Table 11: Extracted MS data used for the calculation of the ^13^C-enrichment of neochlorogenic acid isotopologues (m/z) together with their signal intensity

| C-isotope | | neochlorogenic acid | |
| --- | --- | --- | --- |
| ^12^C | ^13^C | *m/z* | intensity |
| 16 | 0 | 353.0882 | 1026362 |
| 15 | 1 | 354.0916 | 966168 |
| 14 | 2 | 355.0949 | 1153580 |
| 13 | 3 | 356.0982 | 1269541 |
| 12 | 4 | 357.1016 | 1298168 |
| 11 | 5 | 358.105 | 1185957 |
| 10 | 6 | 359.1083 | 975929 |
| 9 | 7 | 360.1117 | 725245 |
| 8 | 8 | 361.1151 | 482980 |
| 7 | 9 | 362.1185 | 293508 |
| 6 | 10 | 363.122 | 158446 |
| 5 | 11 | 364.1255 | 77026 |
| 4 | 12 | 365.1289 | 34640 |
| 3 | 13 | 366.1322 | 13571 |
| 2 | 14 | 367.1358 | 4922 |
| 1 | 15 | 368.139 | 1398 |
| 0 | 16 | 369.1418 | 262 |
| ^13^C-incorporation | 26% | R | 0.92 |

Table 12: Extracted MS data which were used for the calculation of the ^13^C-enrichment of the neochlorogenic acid isotopologues (m/z) together with their signal intensity

|  | quinic acid | | | Caffeic acid | | |
| --- | --- | --- | --- | --- | --- | --- |
|  |  | | |  | | |
|  | 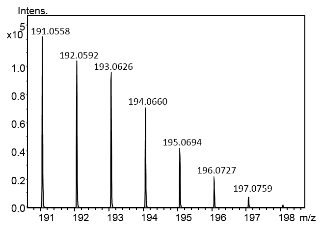 | | | 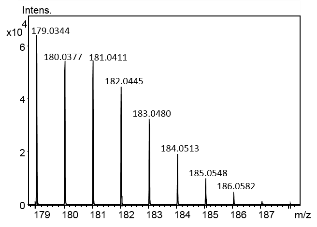 | | |
| ^13^C | *m/z* | intensity |  | *m/z* | intensity |  |
| 0 | 191.0558 | 118521 |  | 179.0344 | 62492 |  |
| 1 | 192.0592 | 101521 |  | 180.0377 | 52821 |  |
| 2 | 193.0626 | 93332 |  | 181.0411 | 52903 |  |
| 3 | 194.066 | 68270 |  | 182.0445 | 43137 |  |
| 4 | 195.0694 | 39765 |  | 183.048 | 30827 |  |
| 5 | 196.0727 | 19549 |  | 184.0513 | 17889 |  |
| 6 | 197.0759 | 7438 |  | 185.0548 | 8521 |  |
| 7 | 198.079 | 1894 |  | 186.0582 | 3560 |  |
| 8 |  |  |  | 187.0588 | 1220 |  |
| 9 |  |  |  | 188.0624 | 305 |  |

Figure 25: Neochlorogenic acid, structure with chemical shifts (MeOH-d_3_), the acquired analytical data is in accordance with previous literature (Choi et al. 2006; Iwai et al. 2004)


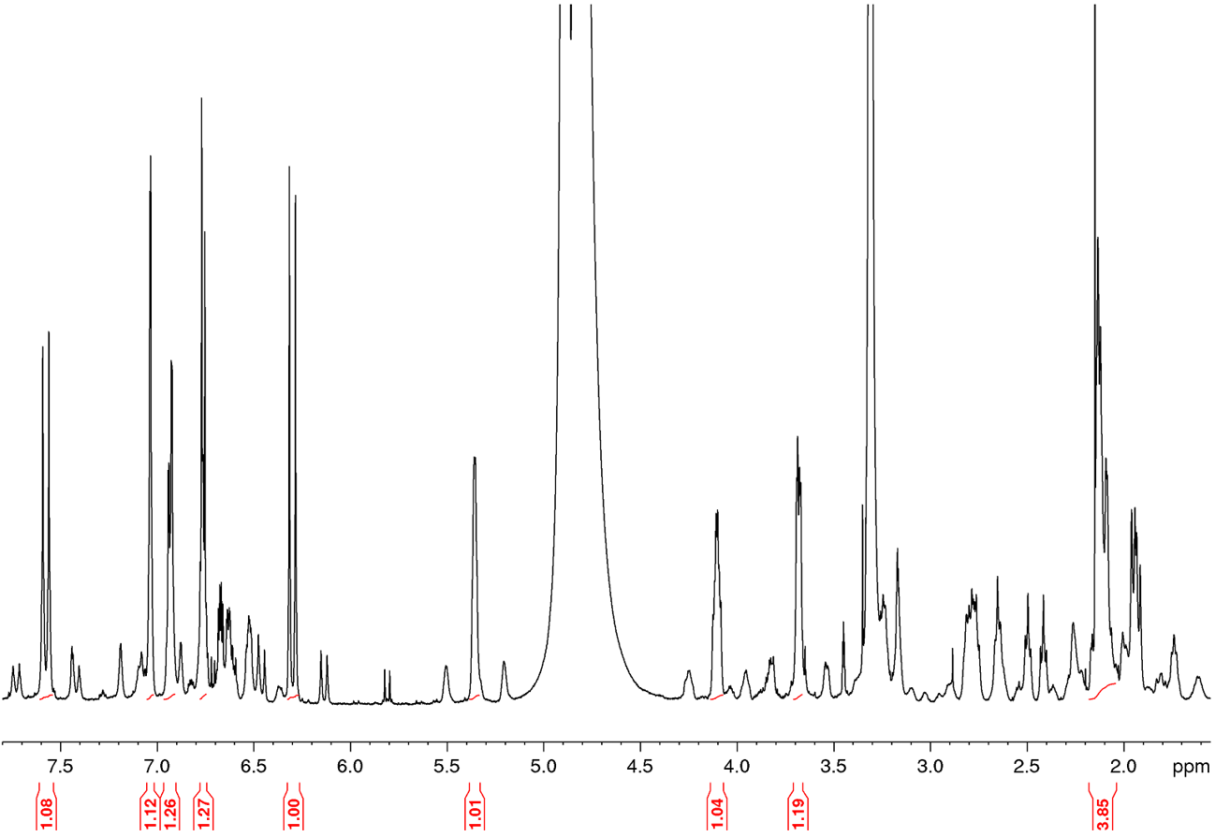


Figure 26: [U-^13^C]neochlorogenic acid, ­^1^H-NMR spectrum (MeOH-d_3_)


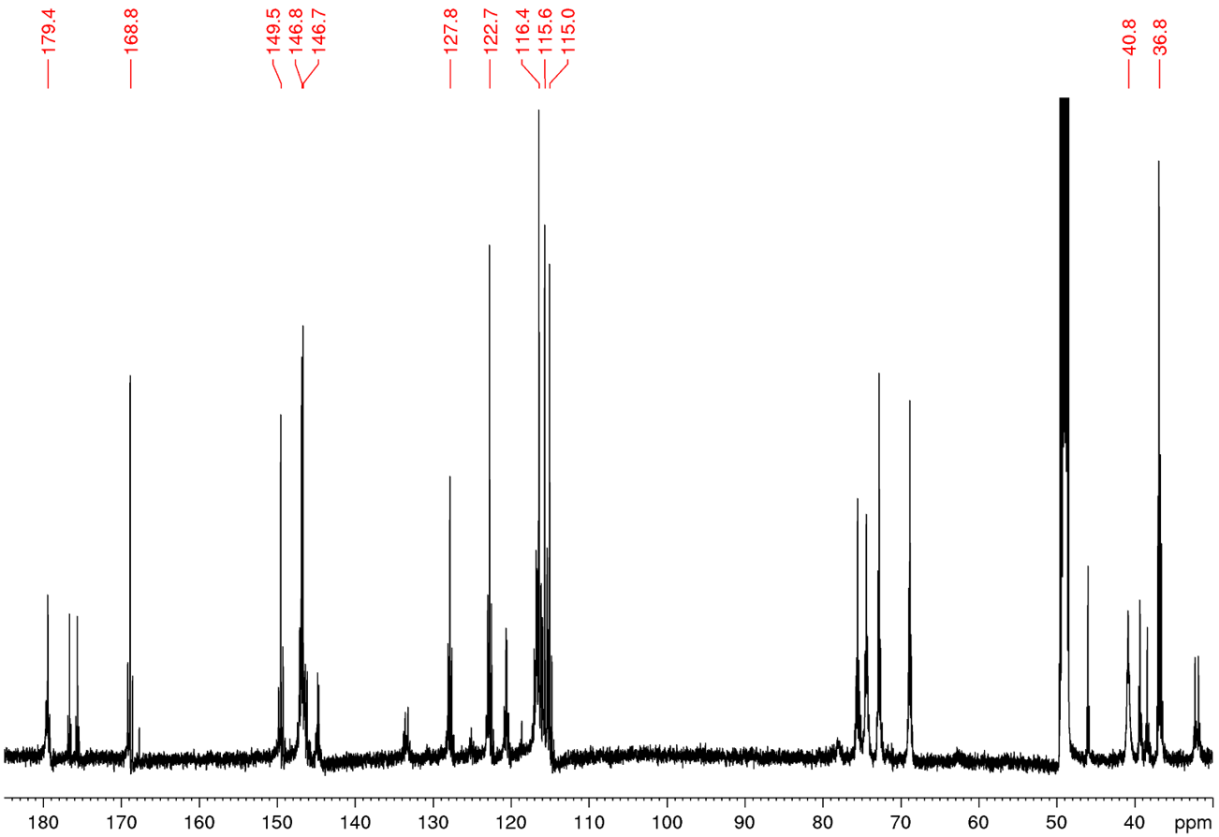


Figure 27: [U-^13^C]neochlorogenic acid, ^13^C-NMR spectrum (MeOH-d_3_)


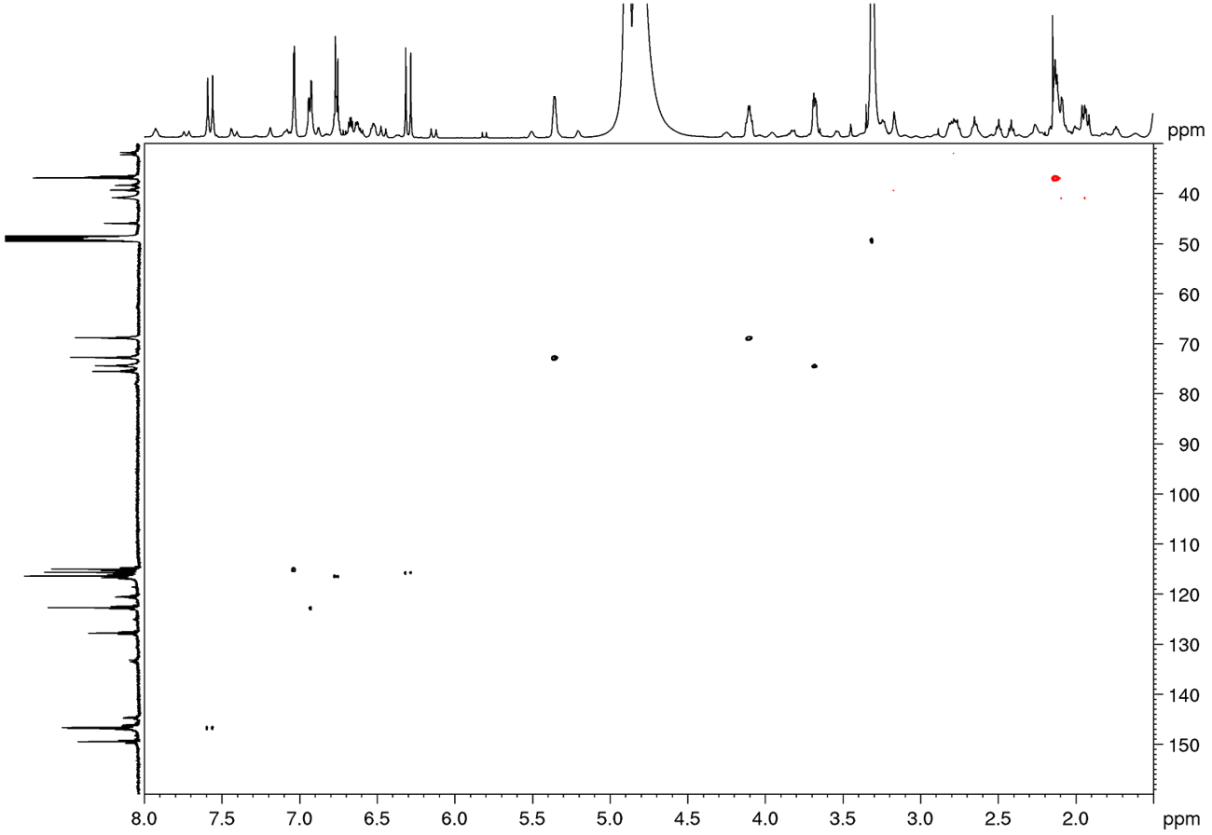


Figure 28: [U-^13^C]neochlorogenic acid, HSQC spectrum (MeOH-d_3_)


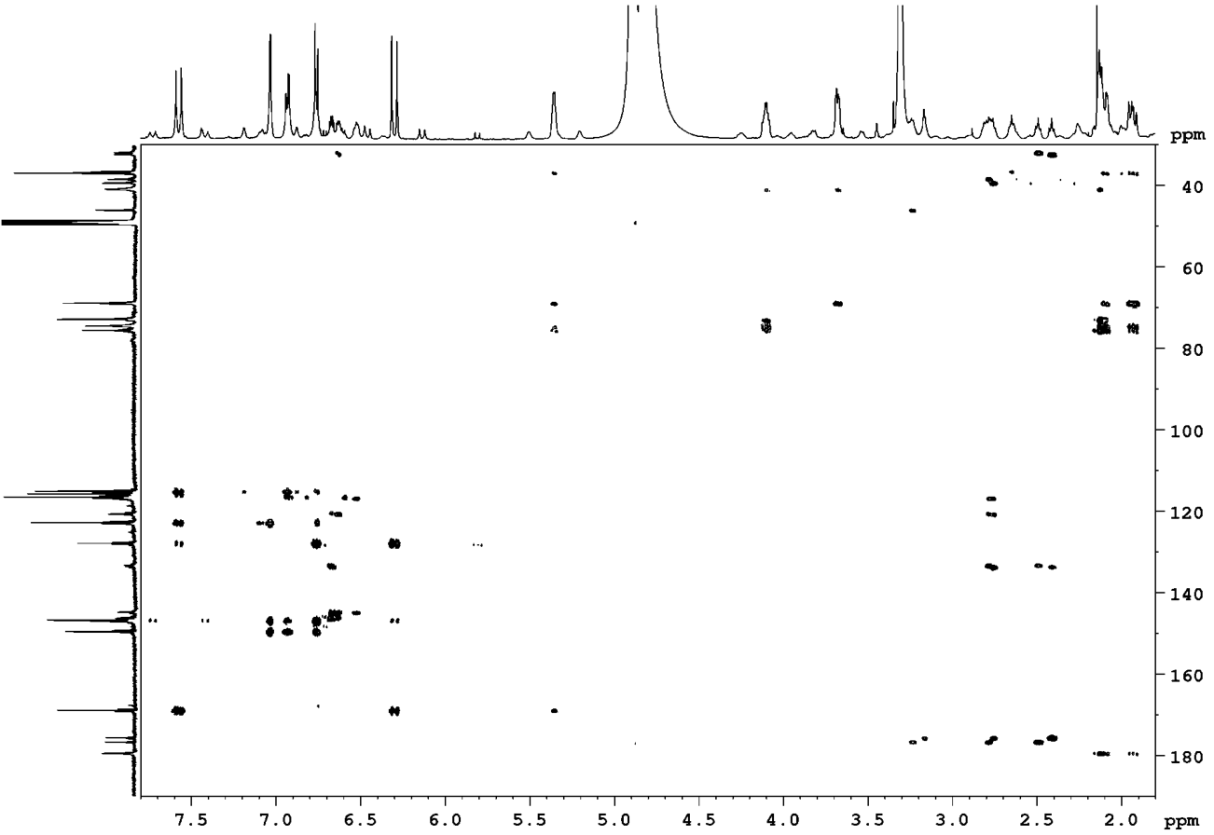


Figure 29: [U-^13^C]neochlorogenic acid, HMBC spectrum (MeOH-d_3_)


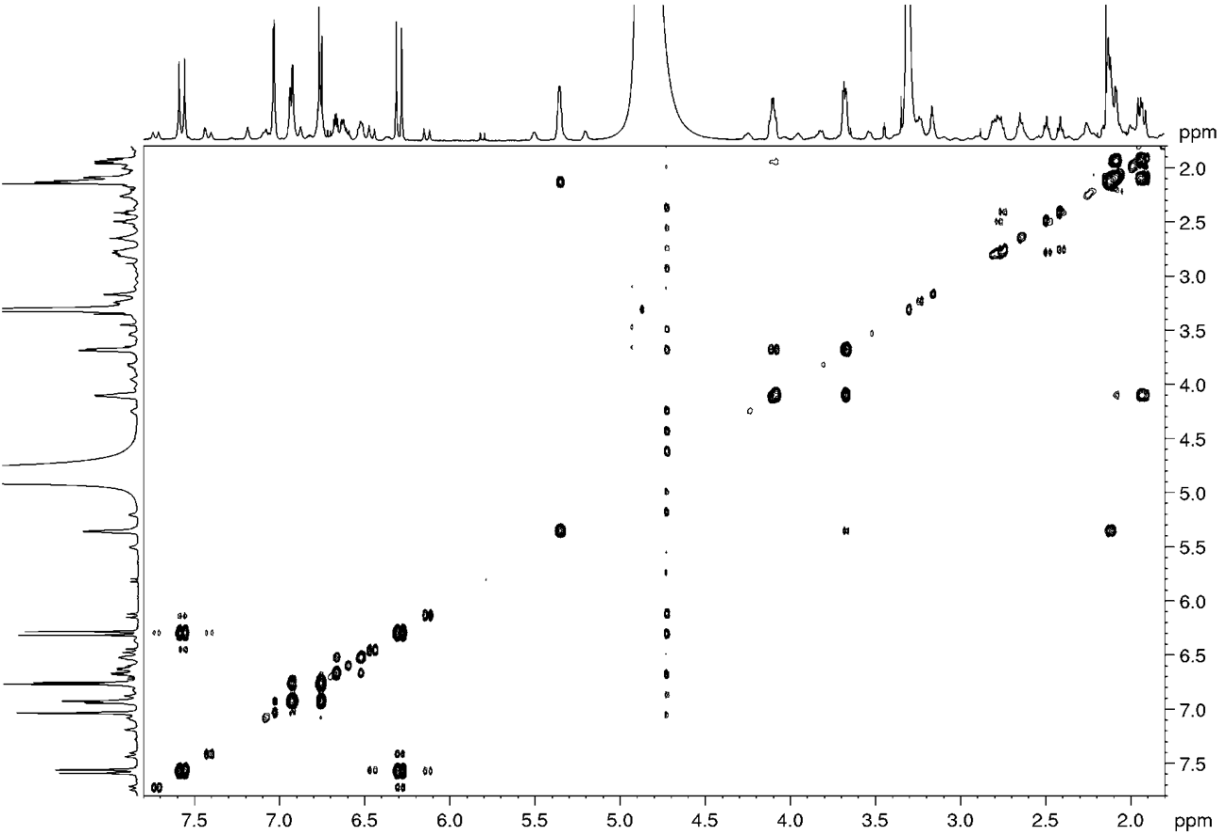


Figure 30: [U-^13^C]neochlorogenic acid, ^1^H-^1^H COSY spectrum (MeOH-d_3_)

## 1.3 Chlorogenic acid methylester


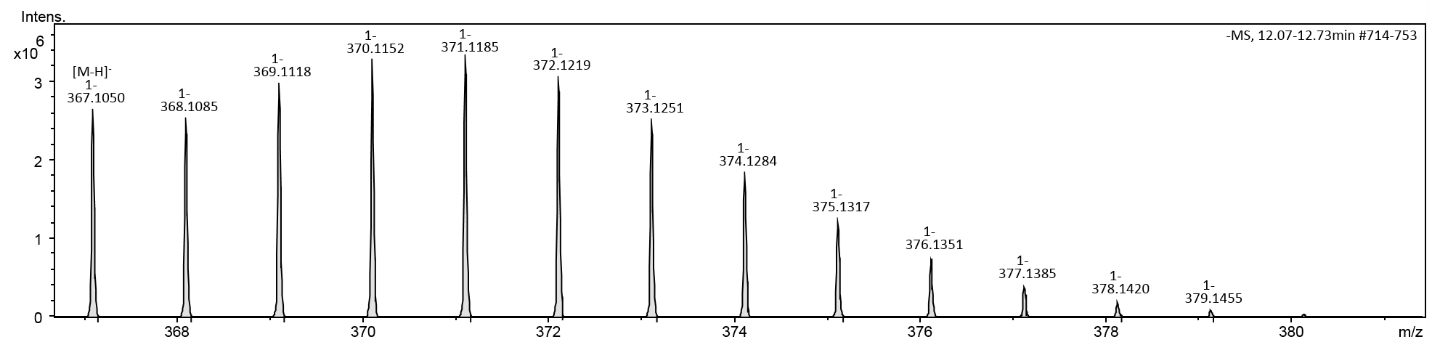


Figure 31: Chlorogenic acid methylester, HRESIMS spectrum, m/z 367.1050 [M-H]^-^

Table 13: Extracted MS data used for the calculation of the ^13^C-enrichment of chlorogenic acidmethylester isotopologues (m/z) together with their signal intensity

| C-isotope | | chlorogenic acid methylester | |
| --- | --- | --- | --- |
| ^12^C | ^13^C | *m/z* | intensity |
| 12 | 0 | 371.1185 | 3259186 |
| 11 | 1 | 372.1219 | 2979535 |
| 10 | 2 | 373.1251 | 2439296 |
| 9 | 3 | 374.1284 | 1765358 |
| 8 | 4 | 375.1317 | 1186270 |
| 7 | 5 | 376.1351 | 712042 |
| 6 | 6 | 377.1385 | 384638 |
| 5 | 7 | 378.142 | 187161 |
| 4 | 8 | 379.1455 | 84515 |
| 3 | 9 | 380.1488 | 33717 |
| 2 | 10 | 381.1523 | 11767 |
| 1 | 11 | 382.1554 | 3424 |
| 0 | 12 | 383.1552 | 662 |
| ^13^C-incoporation | 24% | R | 0.93 |

Table 14: Extracted MS data used for the calculation of the ^13^C-enrichment of chlorogenic acid methylester isotopologues (m/z) together with their signal intensity

|  | Caffeic acid quinone | | |
| --- | --- | --- | --- |
|  |  | | |
|  | 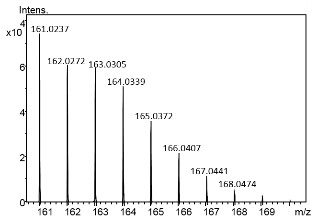 | | |
| ^13^C | *m/z* | intensity |  |
| 0 | 179.0344 | 62492 |  |
| 1 | 180.0377 | 52821 |  |
| 2 | 181.0411 | 52903 |  |
| 3 | 182.0445 | 43137 |  |
| 4 | 183.048 | 30827 |  |
| 5 | 184.0513 | 17889 |  |
| 6 | 185.0548 | 8521 |  |
| 7 | 186.0582 | 3560 |  |
| 8 | 187.0588 | 1220 |  |
| 9 | 188.0624 | 305 |  |

Figure 32: Chlorogenic acid methyl ester, structure with chemical shifts (MeOH-d_3_), the acquired analytical data is in accordance with previous literature (Zhu et al. 2005)


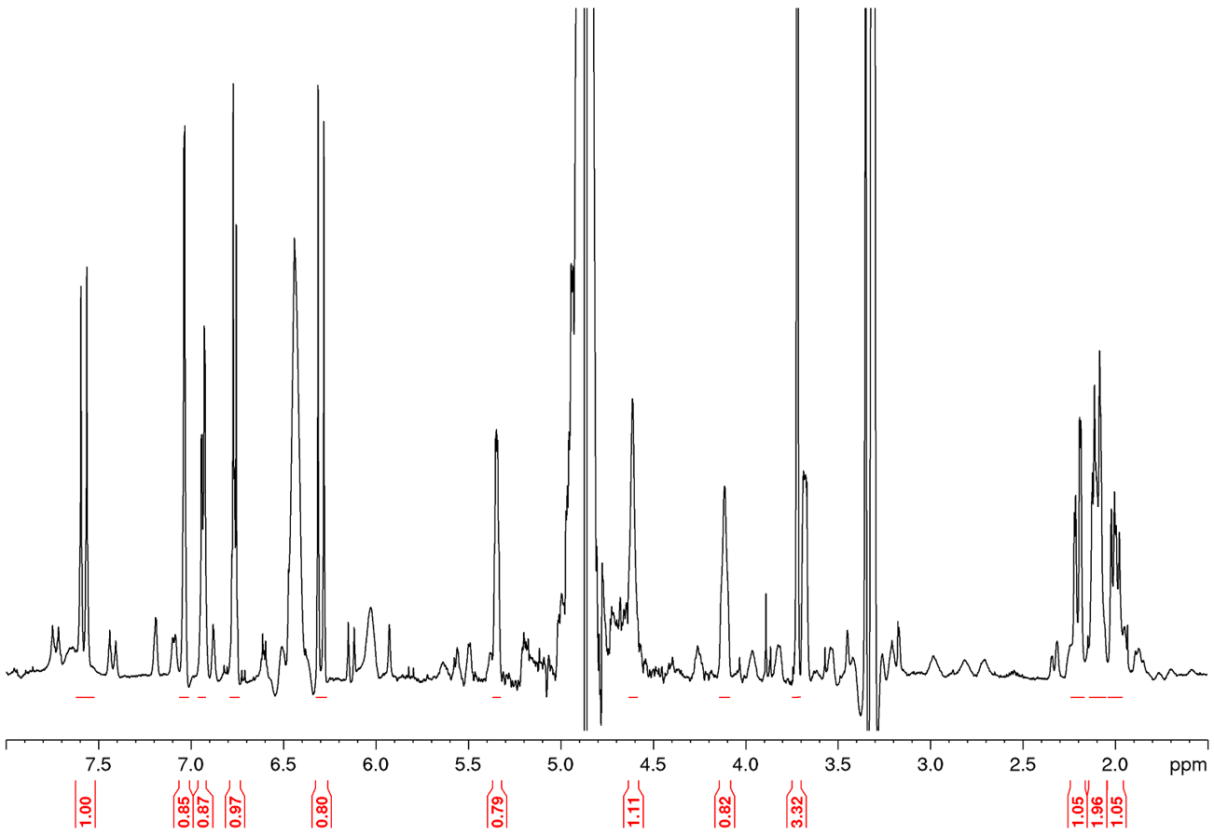


Figure 33: [U-^13^C]chlorogenic acid methylester, ­^1^H-NMR spectrum (MeOH-d_3_)


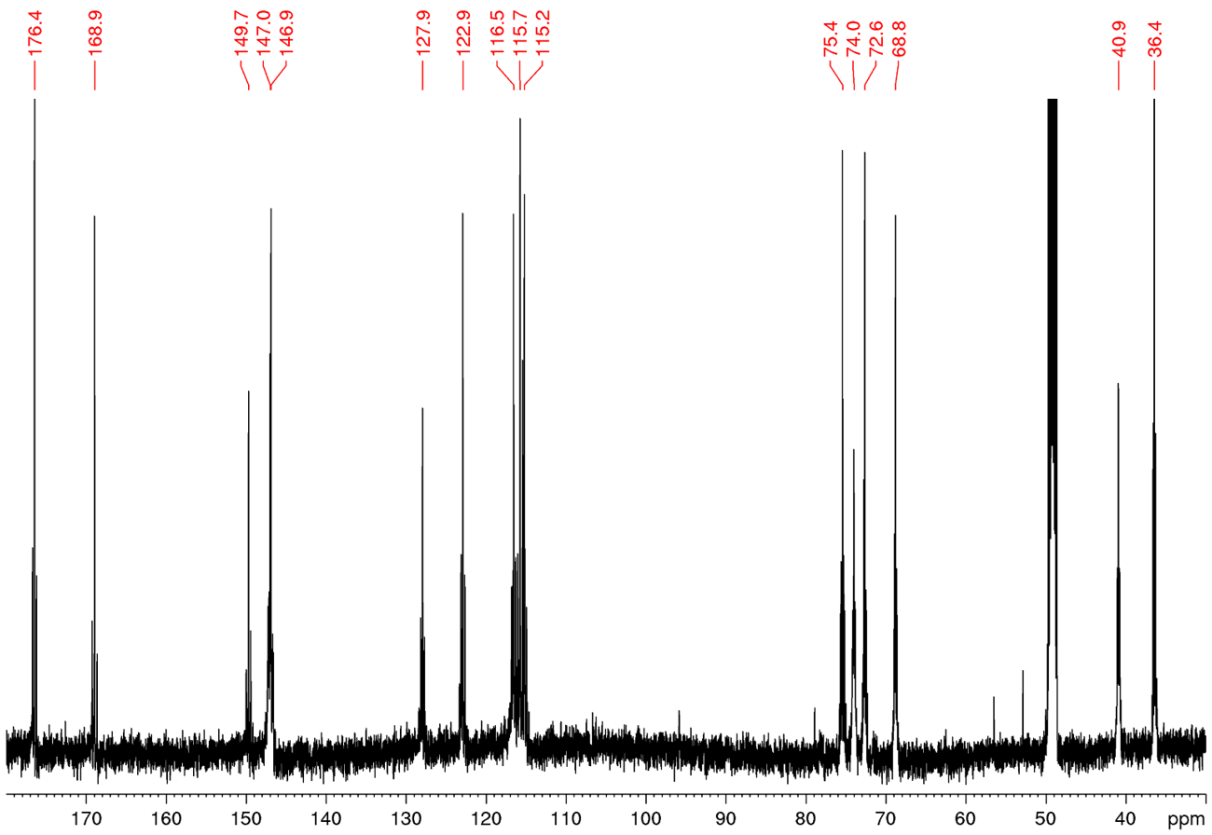


Figure 34: [U-^13^C]chlorogenic acid methylester, ^13^C-NMR spectrum (MeOH-d_3_)


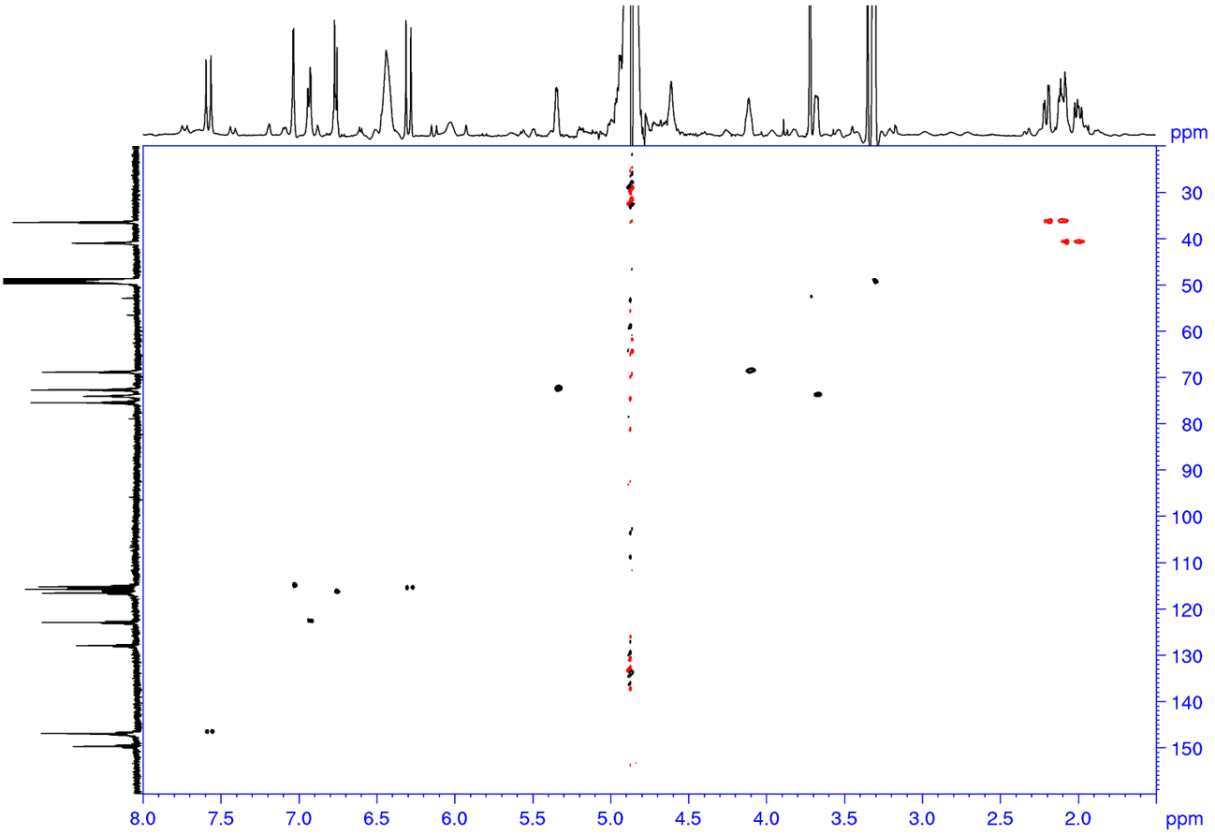


Figure 35: [U-^13^C]chlorogenic acid methylester, HSQC spectrum (MeOH-d_3_)


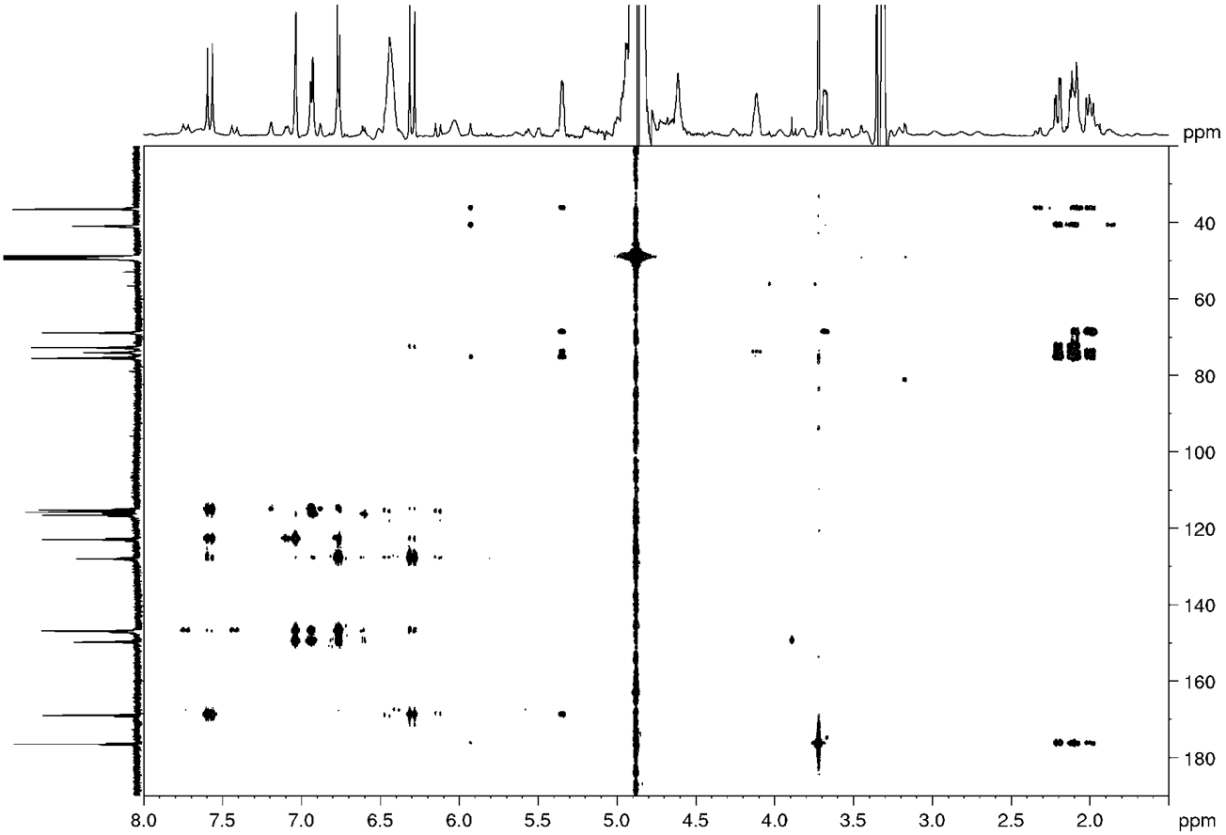


Figure 36: [U-^13^C]chlorogenic acid methylester, HMBC spectrum (MeOH-d_3_)


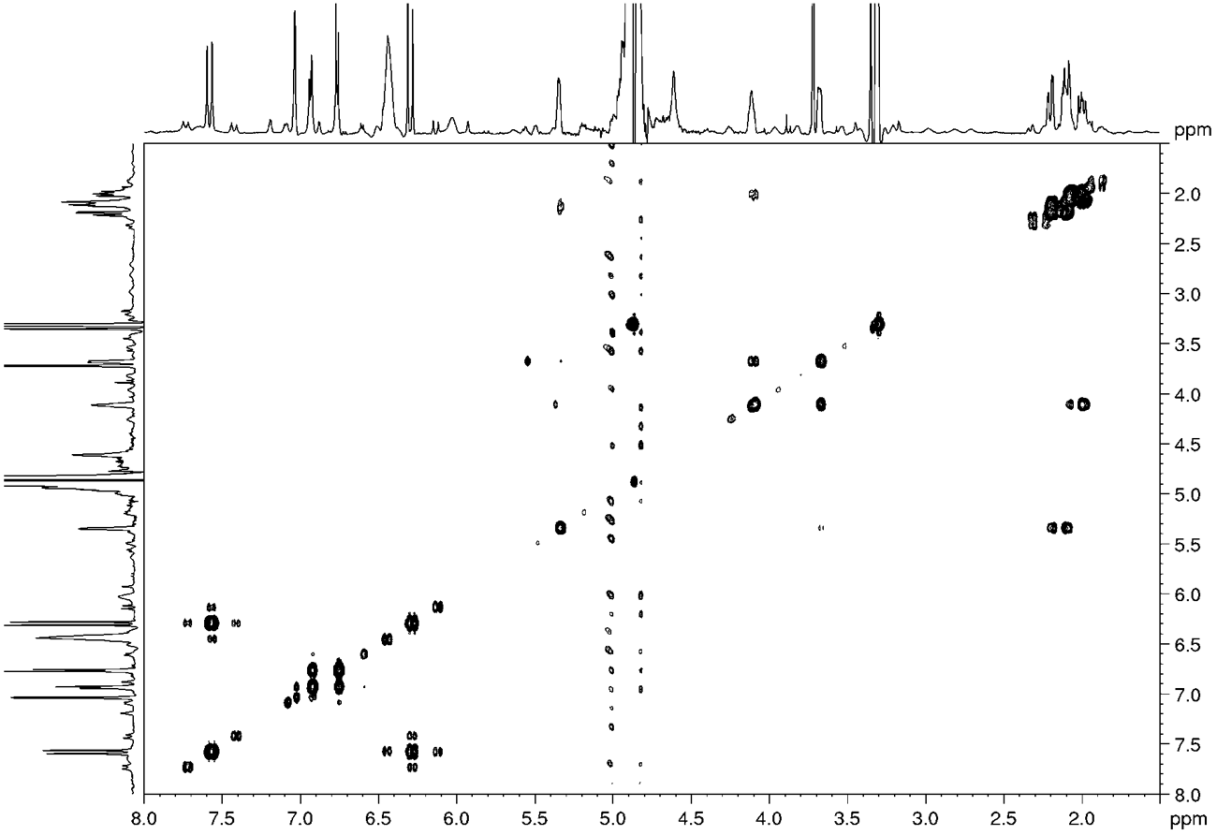


Figure 37: [U-^13^C]chlorogenic acid methylester, ^1^H-^1^H COSY spectrum (MeOH-d_3_)

# Identification of metabolites

Metabolite **22** has a molecular formula of C_21_H_19_O_8_, as determined by the HRESIMS ion at *m*/*z* 399.1082 [M-H] ^-^ (calcd for C_21_H_19_O_8_, *m/z* 399.1085). The ^1^H NMR, ^1^H-^1^H COSY, ^1^H-^13^C HSQC, and the ^1^H-^13^C HMBC spectra of compound **19** showed signals for a quinate and two benzoyl moieties. The ^1^H NMR spectrum displays characteristic deshielding for H-3 (δ_H_ 5.81) and H-4 (δ_H_ 5.40), suggesting a bis-substituted quinate. The position of the substituents was determined by ^1^H-^13^C long-range correlations of H-3 to δ_C_ 167.2 (C-7′) and of H-4 to δ_C_ 167.3 (C-7″). The latter ^13^C shifts were identified to belong to the carboxyl groups of the respective benzoyl moieties. The chemical shifts for both benzoyl units were extracted from ^1^H-^1^H COSY, ^1^H-^13^C HMBC, and ^1^H-^13^C HSQC data. Consequently, compound **19** was elucidated as 3,4-*O*-bisbenzoyl quinic acid.

Interpretation of the HRESIMS data of compounds **20** (*m/z* 399.1071 [M-H]^−^) and **24** (399.1073 [M-H]^−^) resulted in determination of a molecular formula of C_21_H_19_O_8_ for both compounds (calcd for C_21_H_19_O_8_, *m/z* 399.1085), similar to compound **22.** Further evidence for the presence of bisbenzoyl-substituted quinates was found in the ^1^H NMR, ^1^H-^1^H COSY, ^1^H-^13^C HSQC, and the ^1^H-^13^C HMBC data, which showed very similar signals as found for **22**. The chemical shifts of H-3 (δ_H_ 5.56) and H-5 (δ_H_ 5.75) of compound **20** were found to be deshielded, indicating substitution in this position. This substitution was supported by ^3^*J*_CH_ correlations of H-3 to δ_C_ 168.0 (C-7′) and of H-5 to δ_C_ 167.8 (C-7″), revealing C-7′ and C-7″ as carboxyl signals of two different benzoyl substituents. Compound **20** was therefore elucidated as 3,5-*O*-bisbenzoyl quinic acid. In **24** H-4 (δ_H_ 5.33) and H-5 (δ_H_ 5.94) were deshielded. The ^3^*J*_CH_ correlations of H-4 to δ_C_ 167.6 (C-7′) and H-5 to δ_C_ 167.4 (C-7″) indicated again carboxy groups attached to the quinate. Accordingly, compound **24** was elucidated as 4,5-*O*-bisbenzoyl quinic acid. All bis-substituted quinates isolated from frass had ^13^C-labeled benzoyl substituents after larvae were fed with ^13^C-tremulacin, whereas no ^13^C-benzoyl label has been found after feeding [U-^13^C]salicortin or [U-^13^C]HCH-salicortin.

## 2.1 3-*O*-Benzoylquinic acid


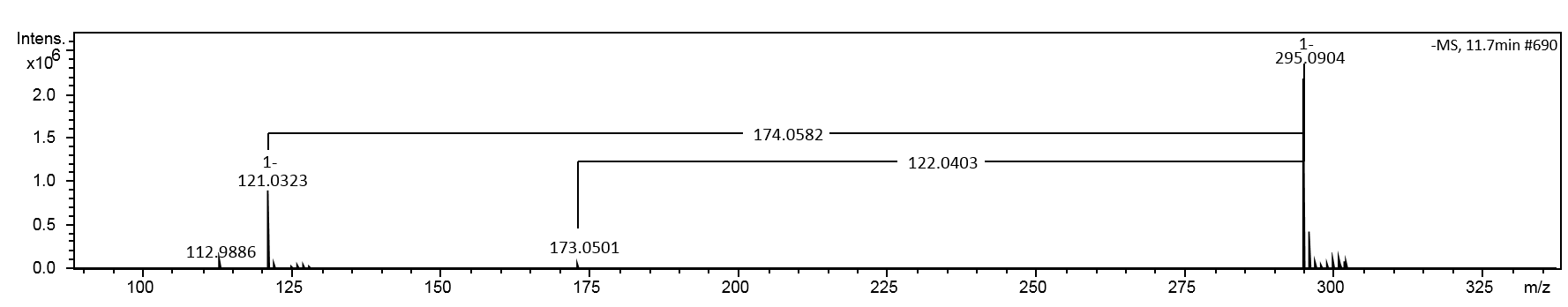


Figure 38: 3-O­-benzoylquinic acid (**19**), HRESIMS spectrum, m/z 295.0904 [M-H]^-^


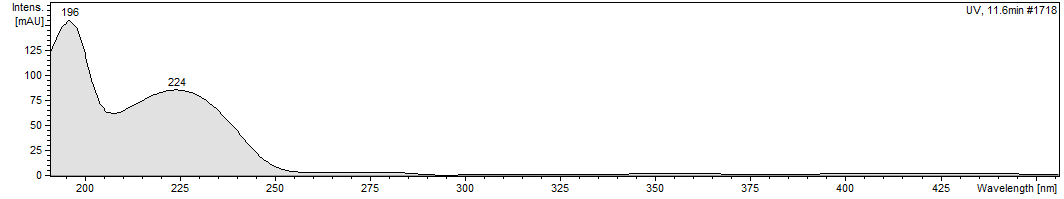


Figure 39: 3-O­-benzoylquinic acid, UV spectrum from HPLC-DAD

Figure 40: 3-O-benzoylquinic acid, structure with chemical shifts (MeOH-d_3_) ), the acquired analytical data is in accordance with previous literature (Toyama-Kato et al. 2007)


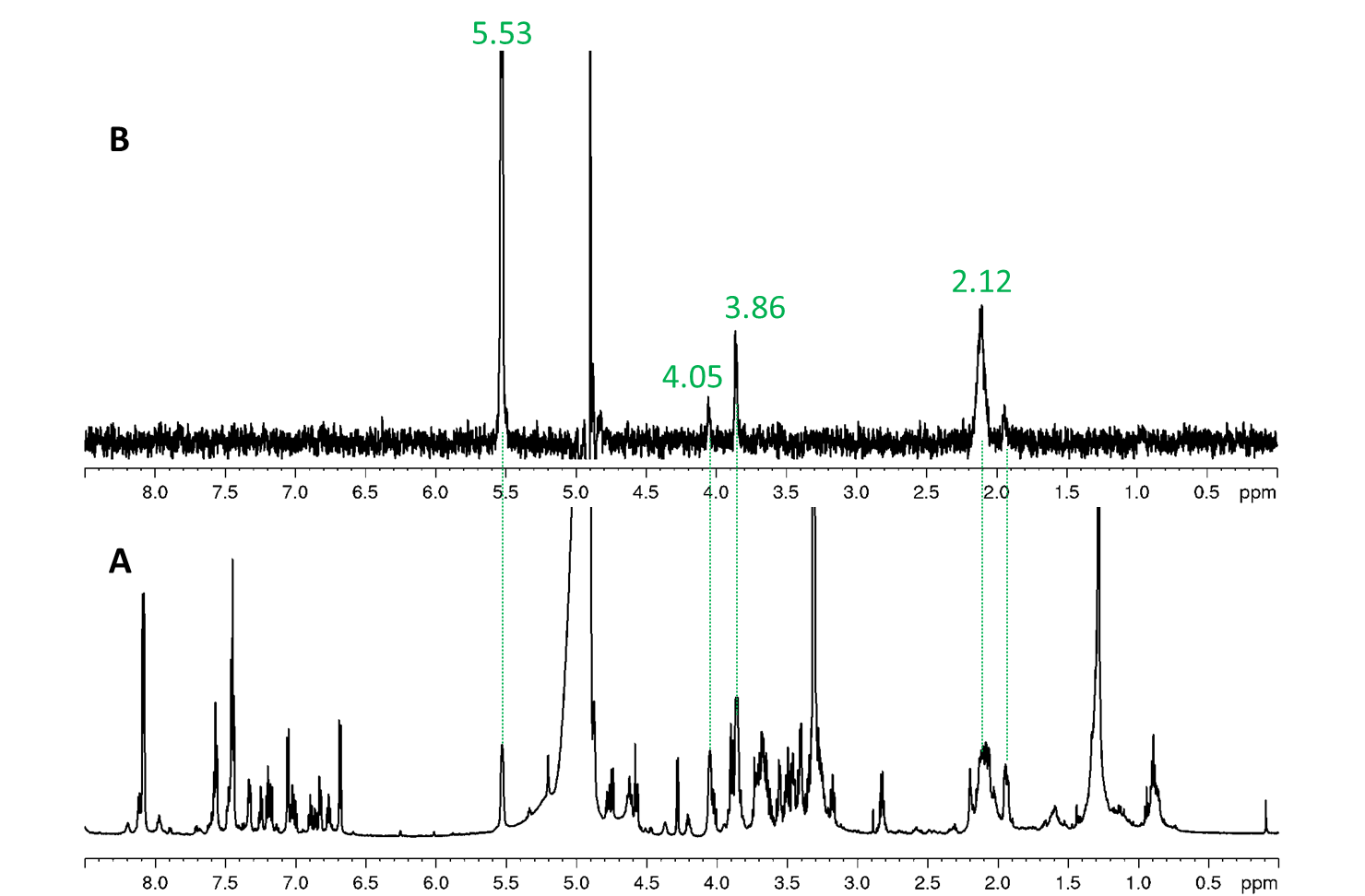


Figure 41: 3-O­-benzoylquinic acid, A, ^1^H-NMR spectrum (700 MHz, MeOH-d_3_); B, SELTOCSY, transmitter set to 5.53 ppm


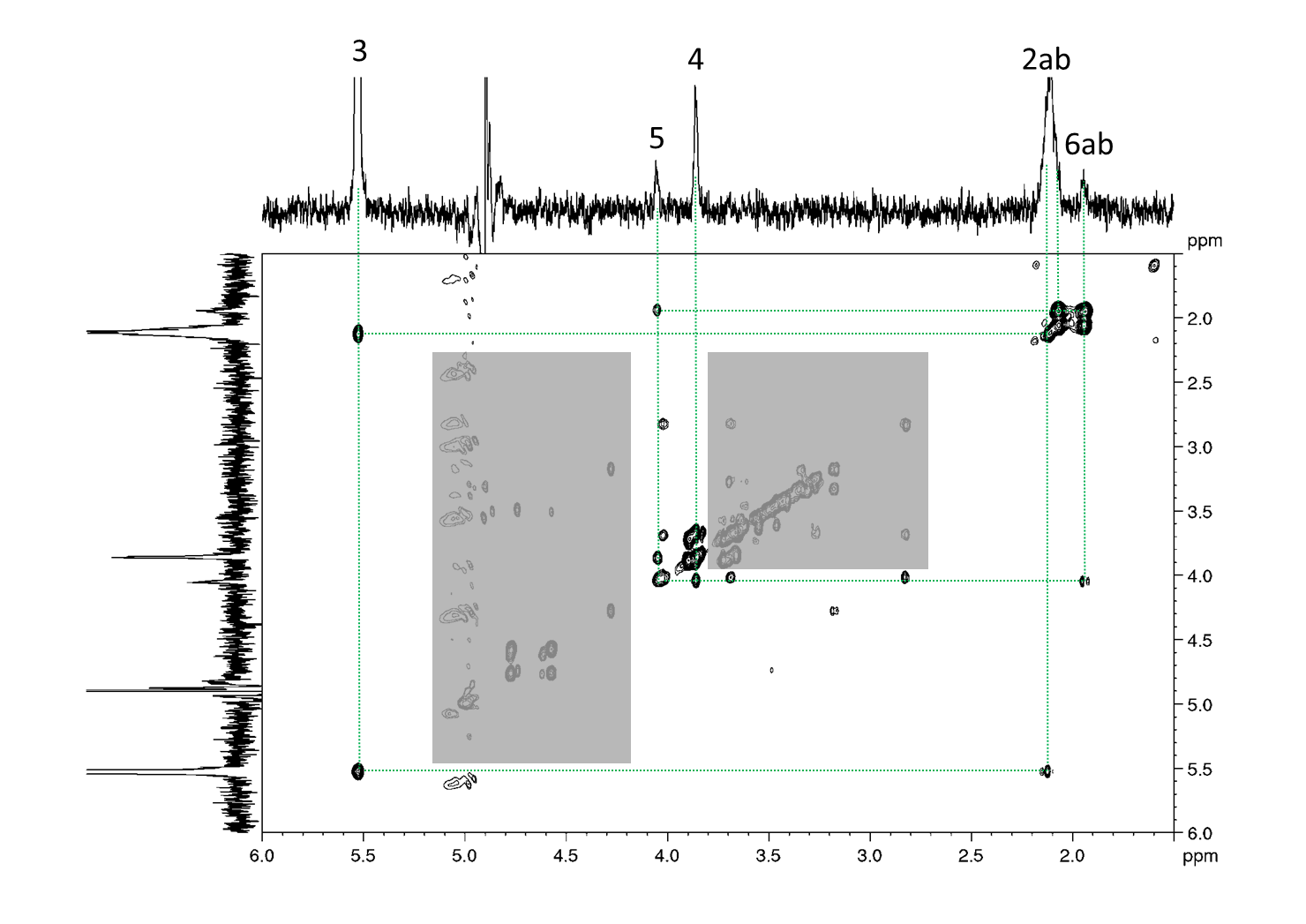


Figure 42: 3-O­-benzoylquinic acid, aliphatic part, COSY spectrum (MeOH-d_3_). The SELTOCSY from Fig.42 was used as projection spectrum in F1 and F2. Some signals of impurities are covered.


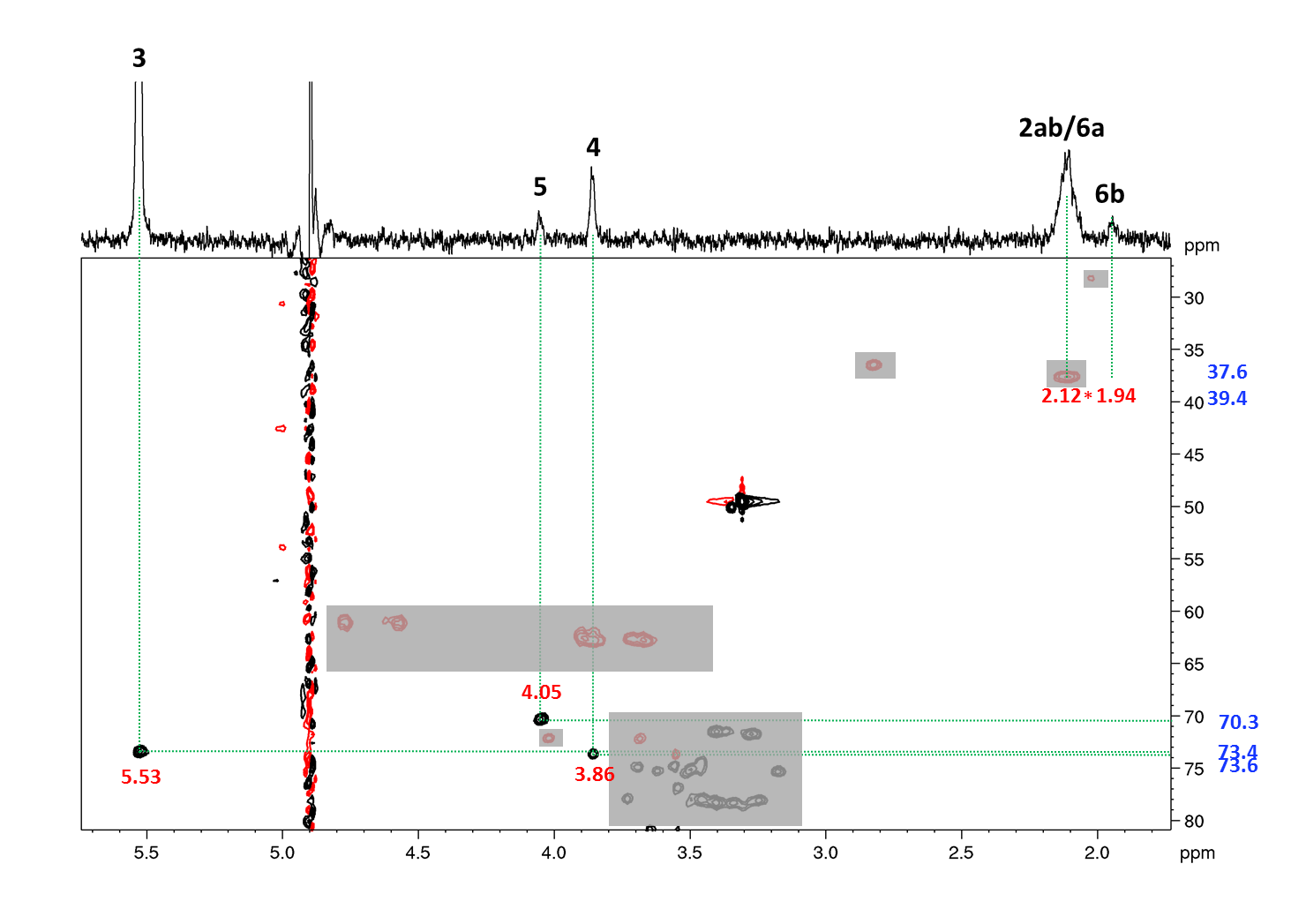


Figure 43: 3-O­-benzoylquinic acid, HSQC, aliphatic part (MeOH-d_3_). *)^13^C signals of positions 2/6 are very weak. Signals of impurities are covered.


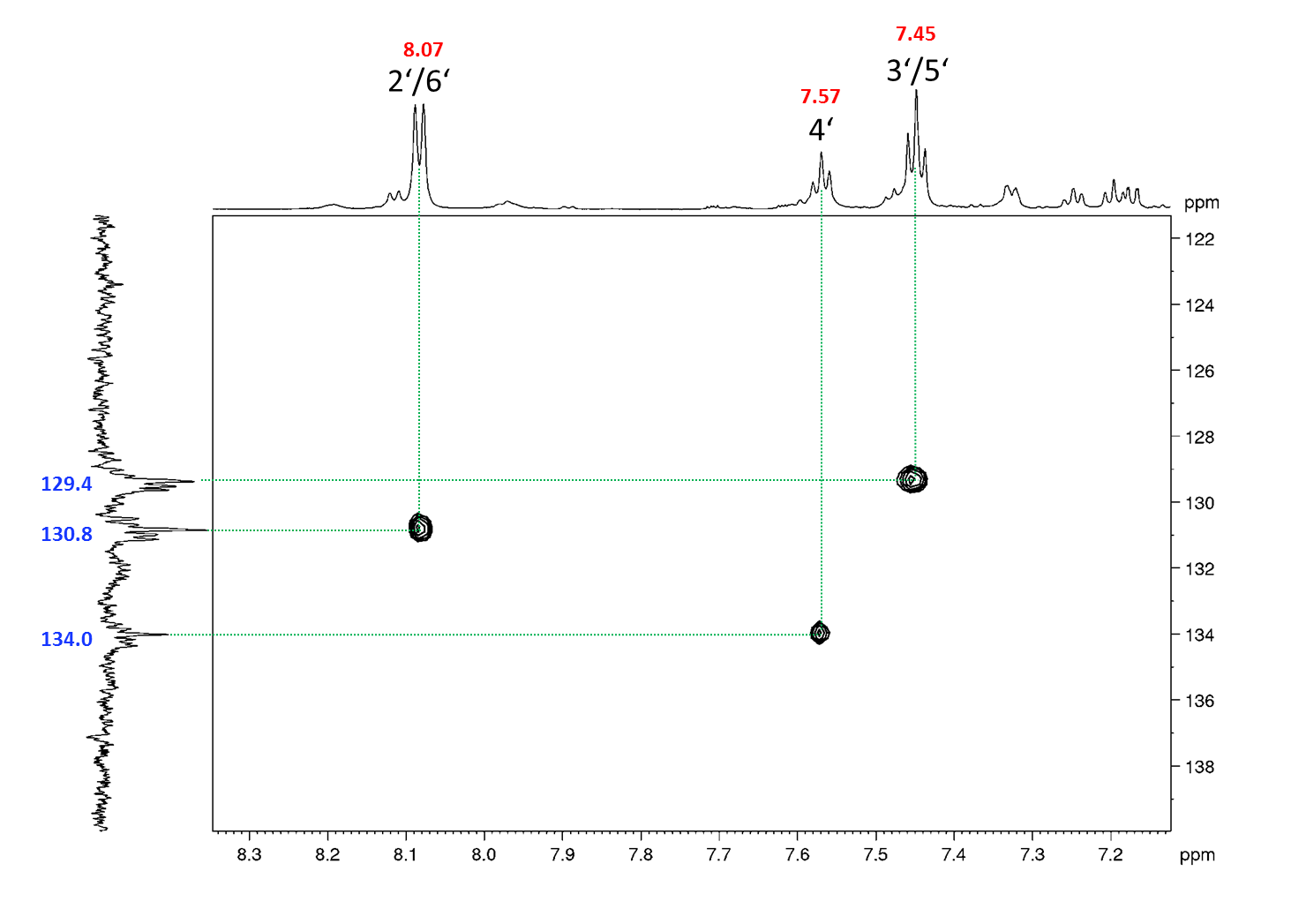


Figure 44: 3-O­-benzoylquinic acid, HSQC, aromatic part (MeOH-d_3_)


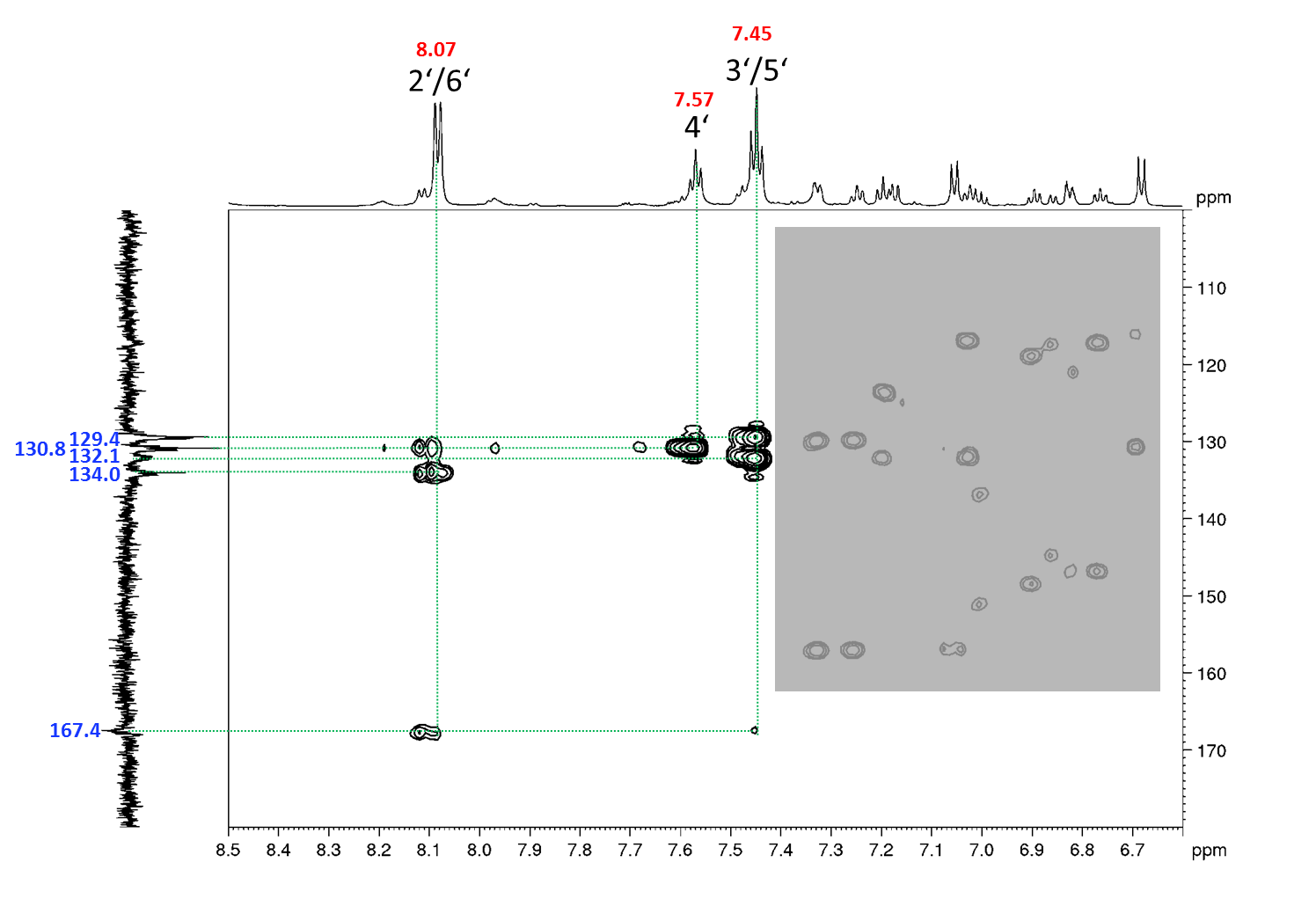


Figure 45: 3-O­-benzoylquinic acid, HMBC spectrum, aromatic part (MeOH-d_3_). Signals of impurities are covered.


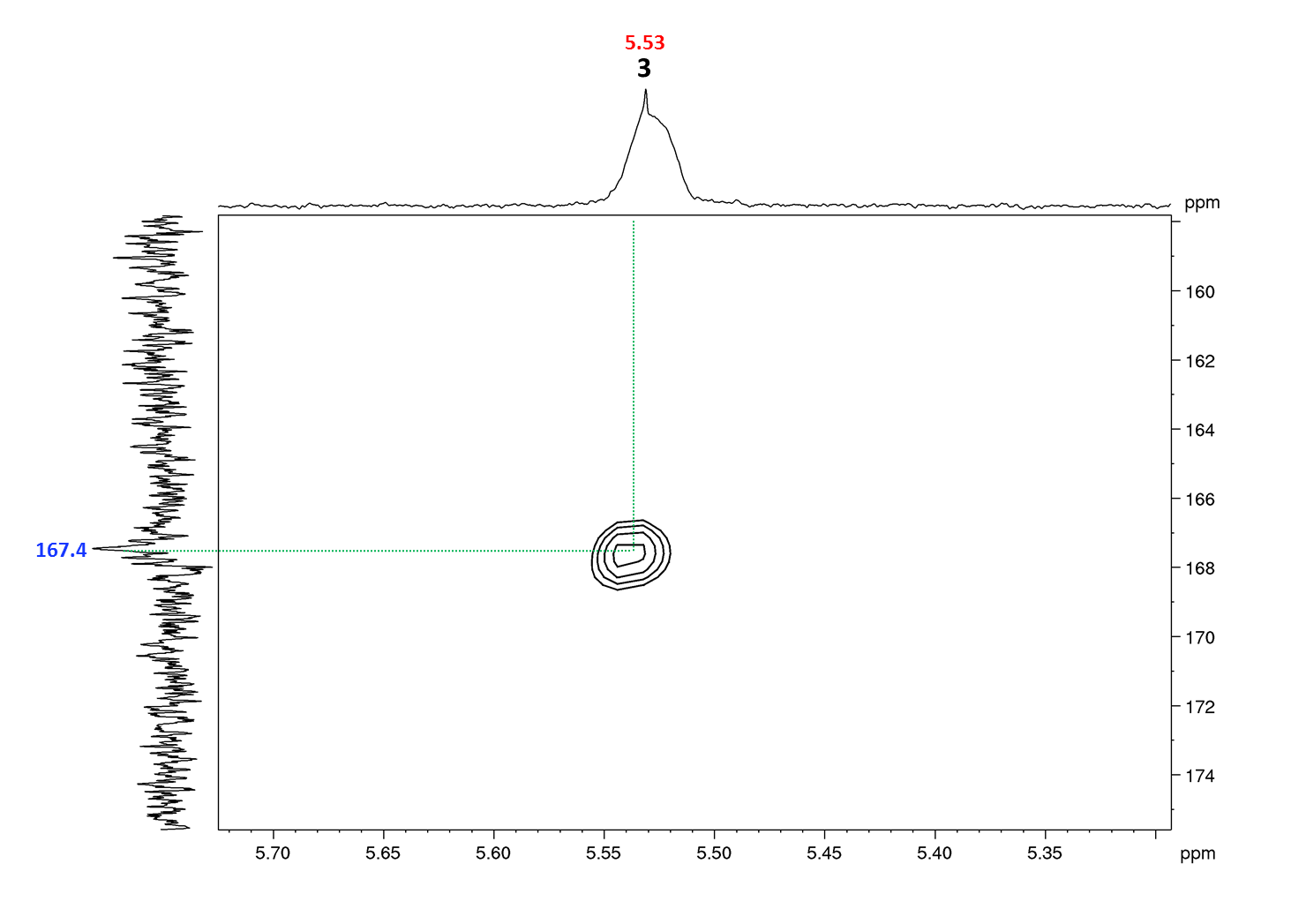


Figure 46: 3-O­-benzoylquinic acid, detail of the HMBC spectrum (MeOH-d_3_). Correlation of H-3 of quinic acid with C-7’ of the benzoyl substituent.

2.2 3,4-*O*-Bisbenzoylquinic acid
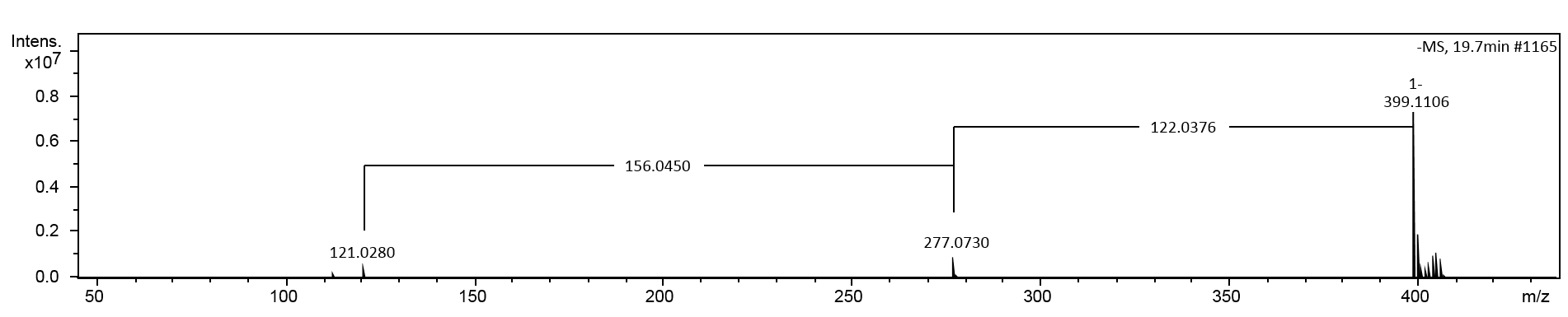


Figure 47: 3,4-O-bisbenzoyl quinic acid, HRESIMS spectrum, m/z 399.1106 [M-H]^-^


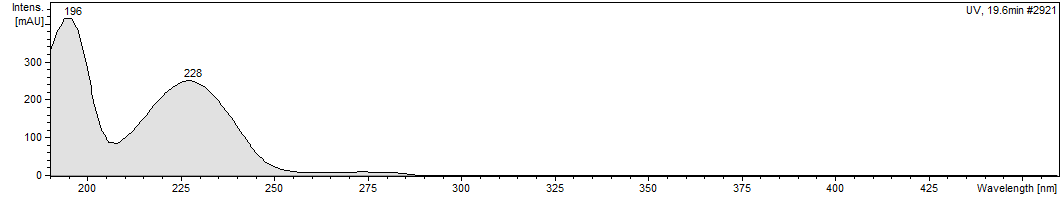


Figure 48: 3­,4-O-bisbenzoyl quinic acid, UV spectrum from HPLC-DAD

Figure 49: 3­,4-O-bisbenzoyl quinic acid, structure with chemical shifts (MeOH-d_3_)


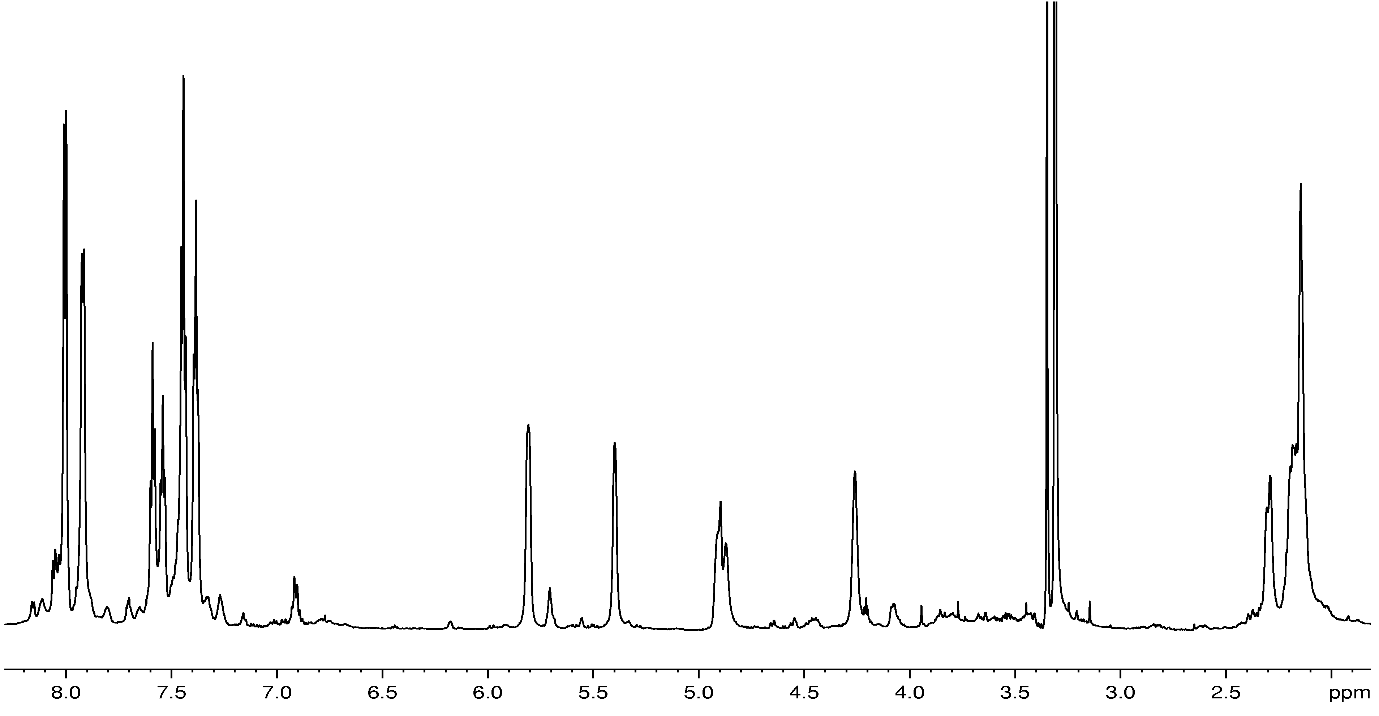


Figure 50: 3,4-O-bisbenzoyl quinic acid, ­^1^H-NMR spectrum (MeOH-d_3_)


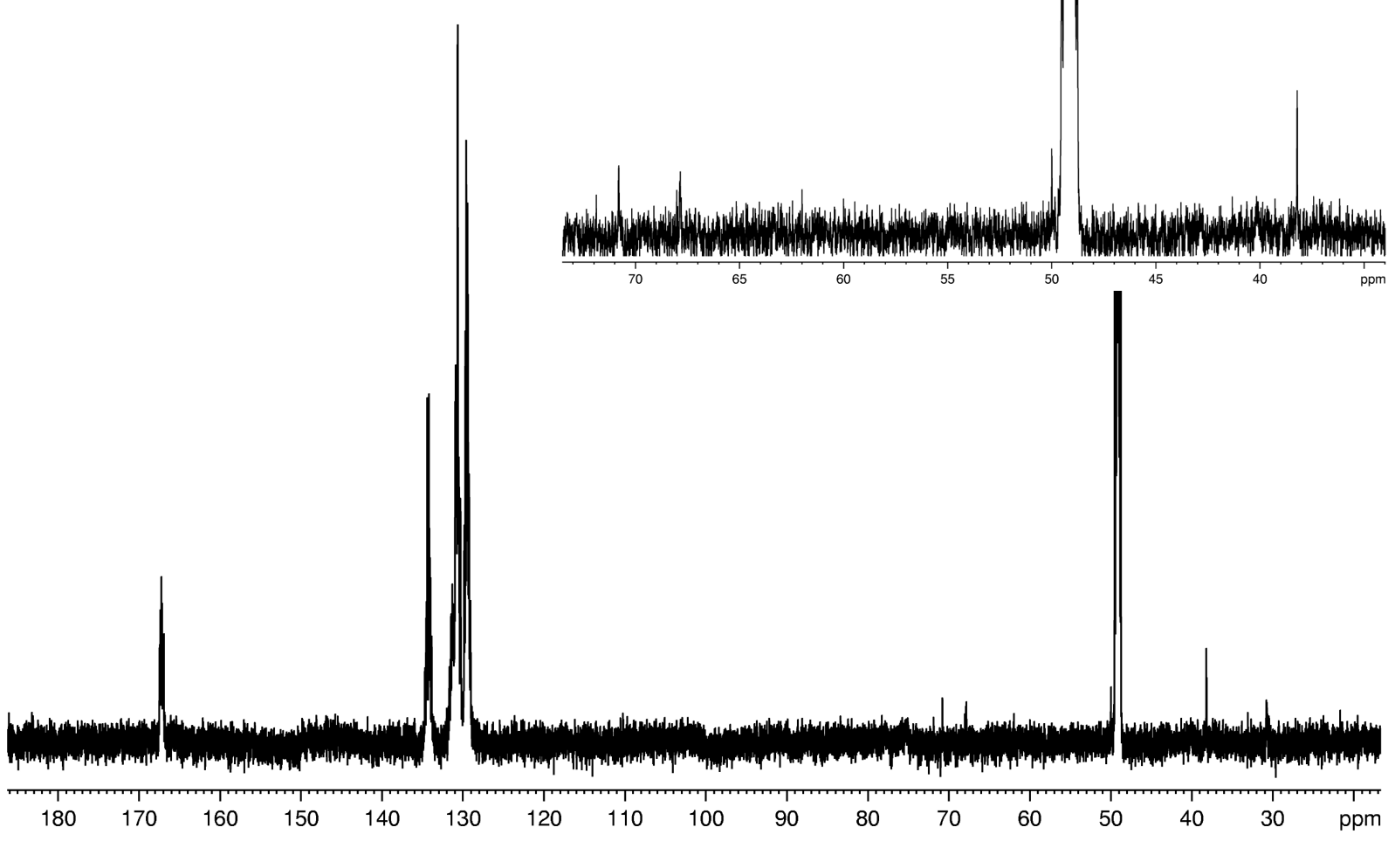


Figure 51: 3,4-O-bisbenzoyl quinic acid, ^13^C-NMR spectrum (MeOH-d_3_)


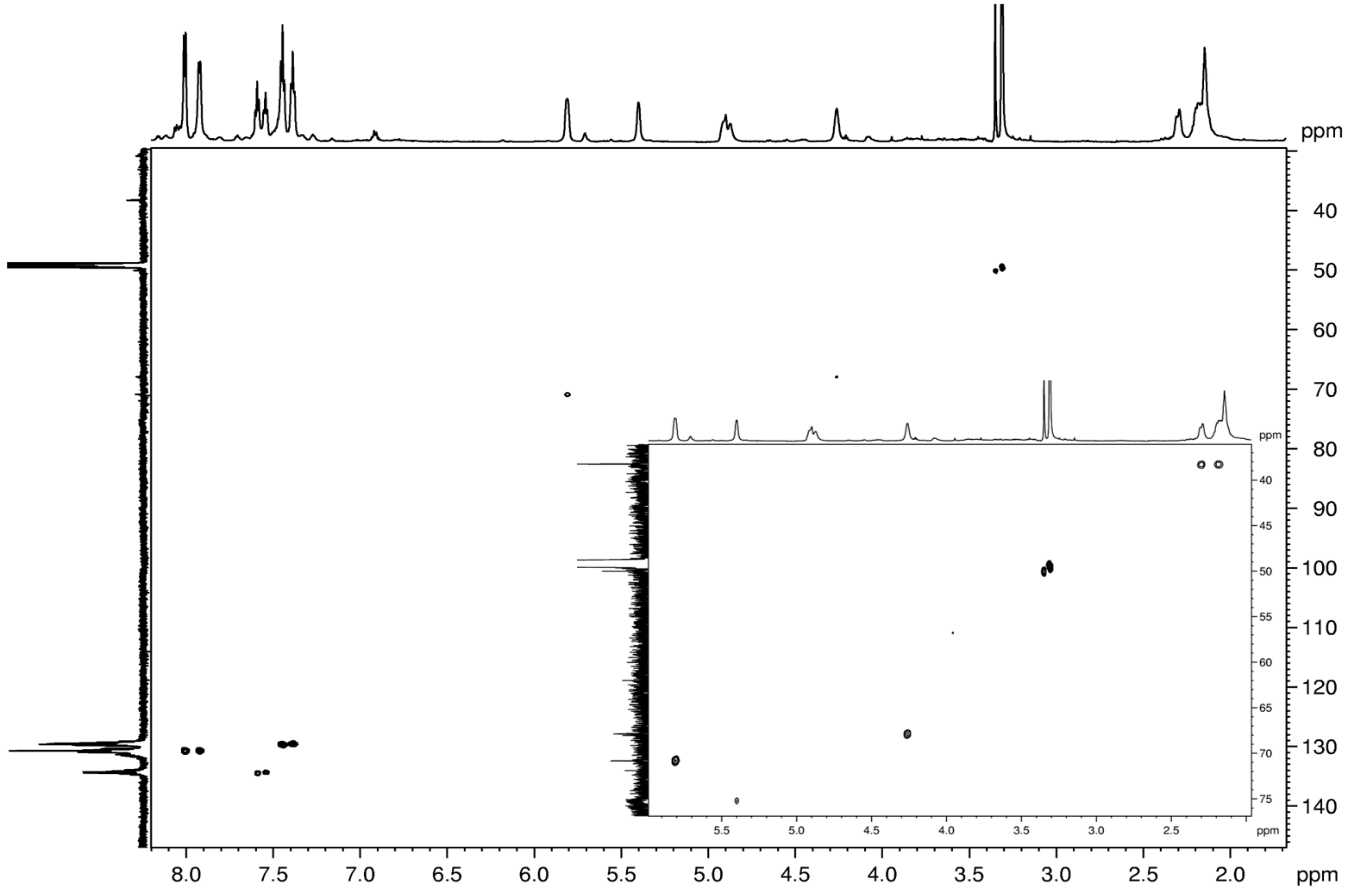


Figure 52: 3­,4-O-bisbenzoyl quinic acid, HSQC spectrum (MeOH-d_3_)


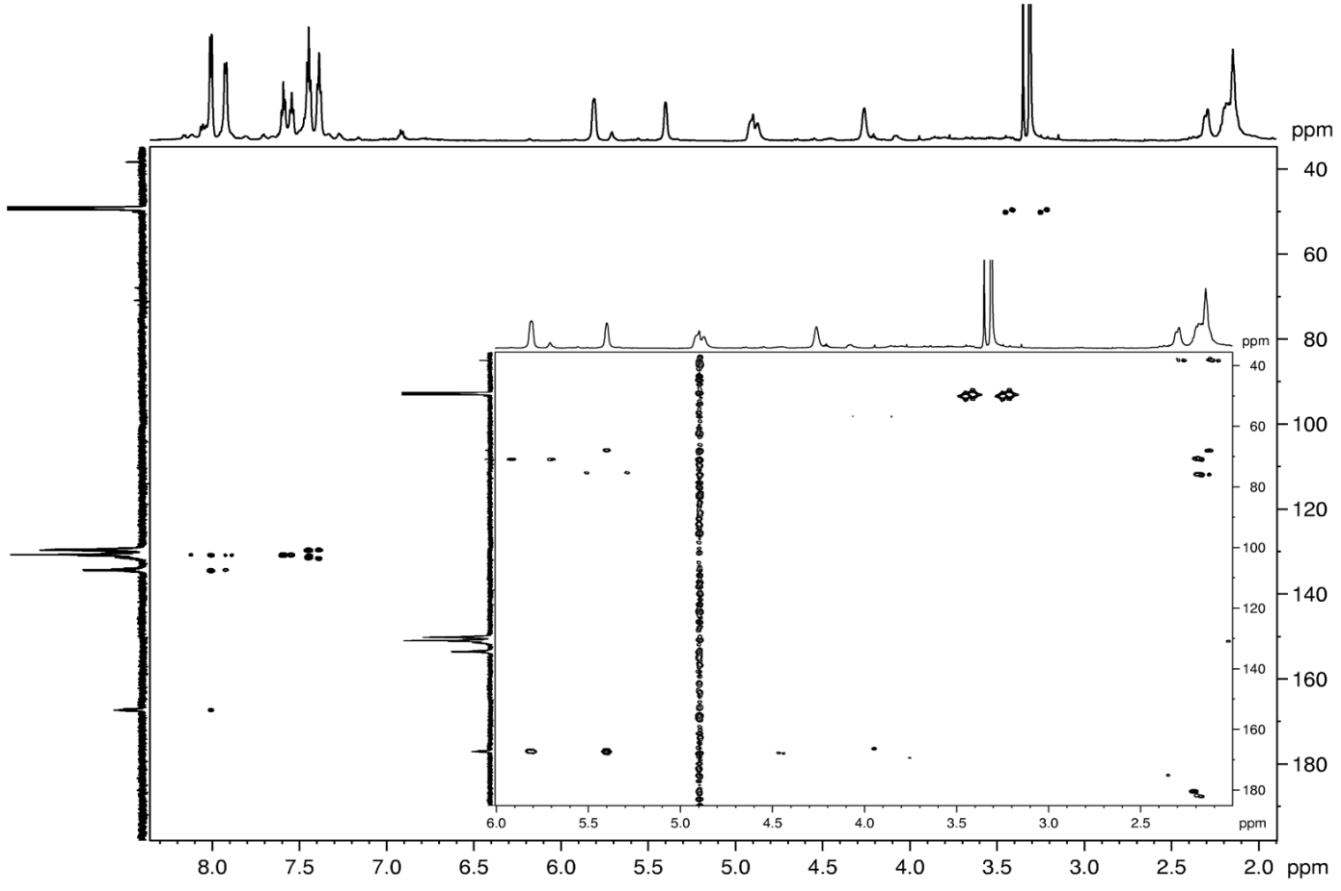


Figure 53: 3,4-O-bisbenzoyl quinic acid, HMBC spectrum (MeOH-d_3_)


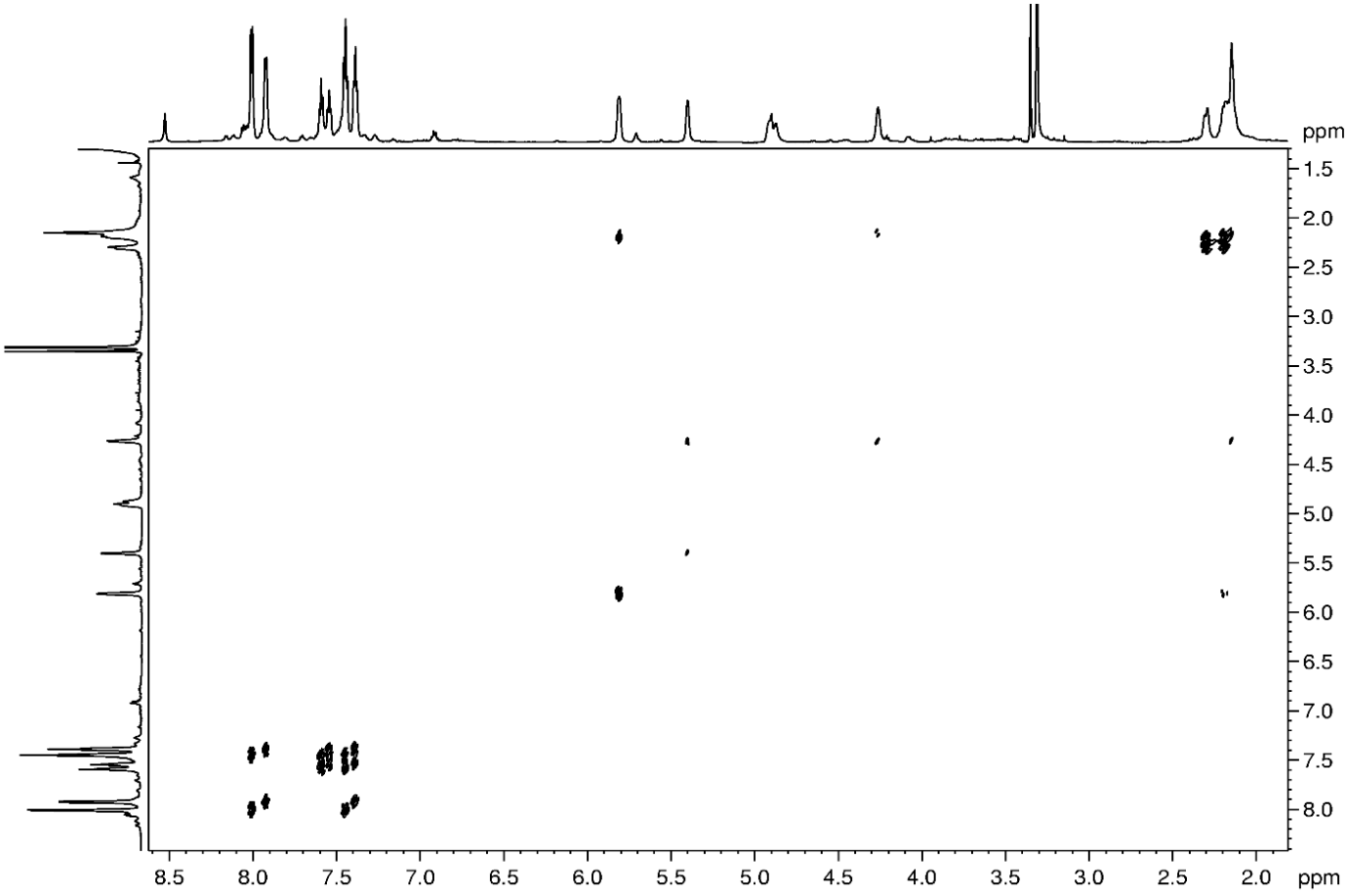


Figure 54: 3,4-O-bisbenzoyl quinic acid, ^1^H-^1^H COSY spectrum (MeOH-d_3_)


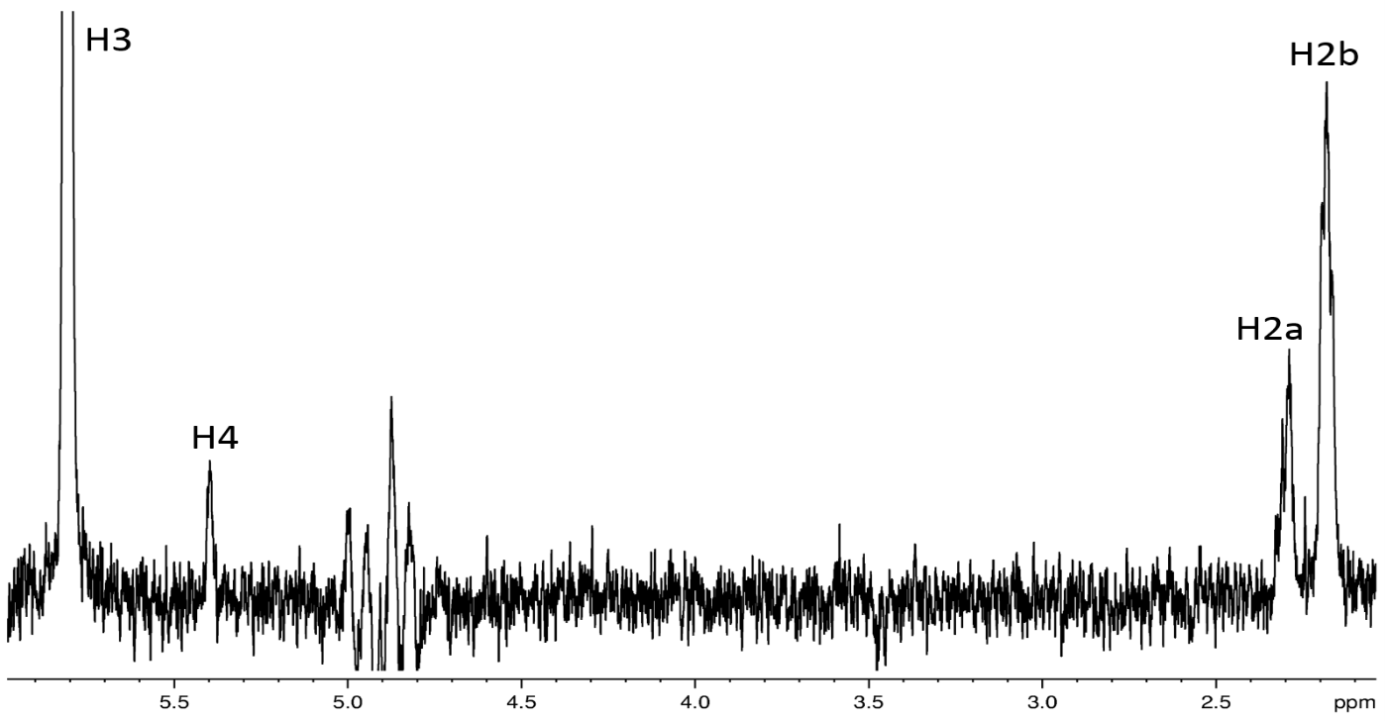


Figure 55: 3,4-O-bisbenzoylquinic acid - selective TOCSY spectrum (MeOH-d_3_, o1p = 5.84 ppm)


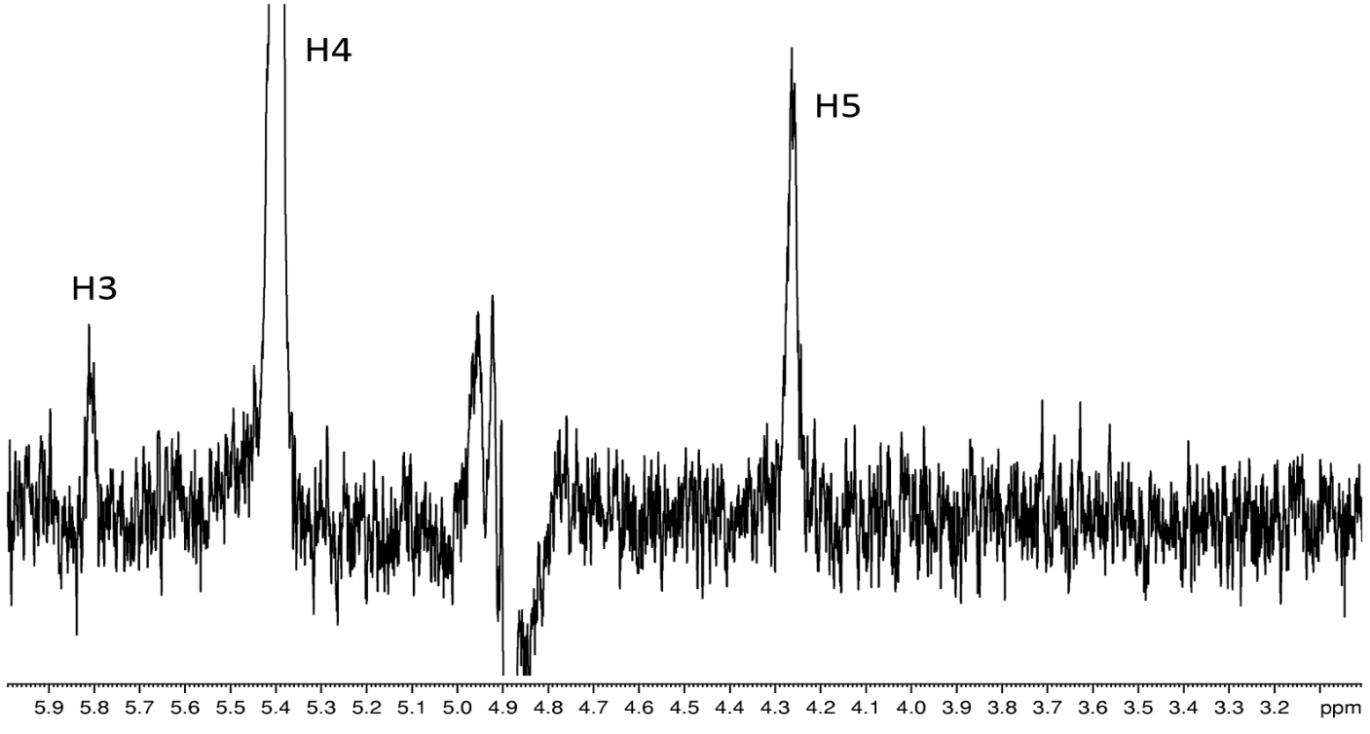


Figure 56: 3,4-O-bisbenzoylquinic acid - selective TOCSY spectrum (MeOH-d_3_, o1p = 5.43 ppm)


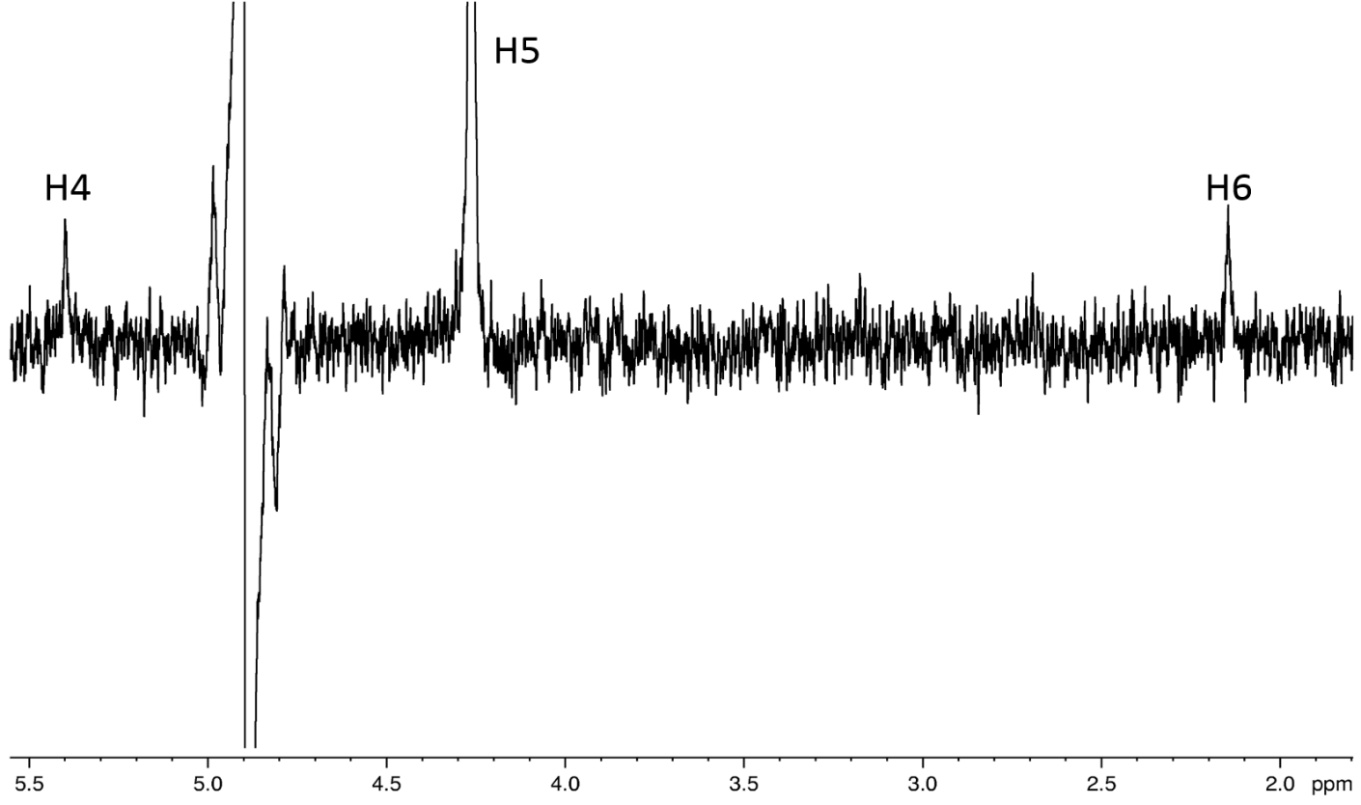


Figure 57: 3,4-O-bisbenzoyl quinic acid - selective 1D TOCSY spectrum (MeOH-d_3_, o1p = 4.29 ppm)

2.3 3,5-*O*-Bisbenzoylquinic acid
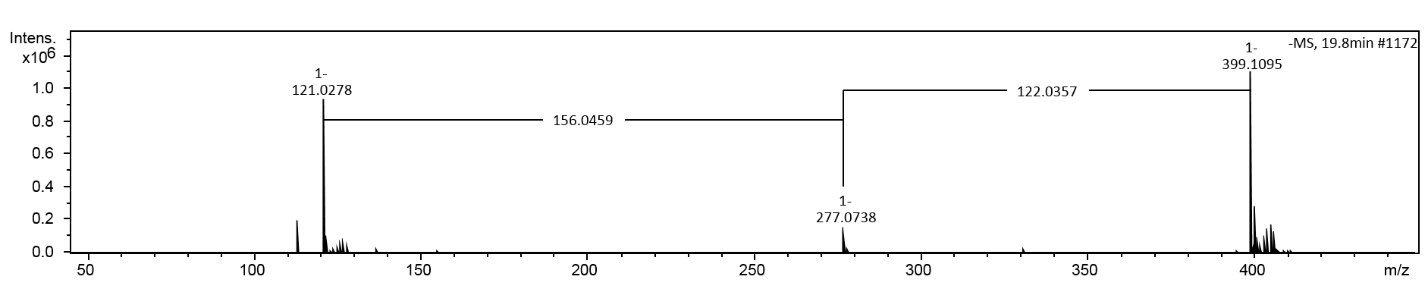


Figure 58: 3,5-O-bisbenzoyl quinic acid, HRESIMS spectrum, m/z 399.1095 [M-H]^-^


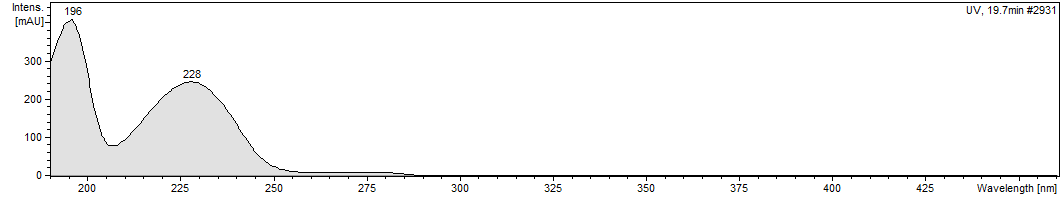


Figure 59: 3­,5-O-bisbenzoyl quinic acid, UV spectrum from HPLC-DAD

Figure 60: 3,5-O-bisbenzoyl quinic acid, structure with chemical shifts (MeOH-d_3_)


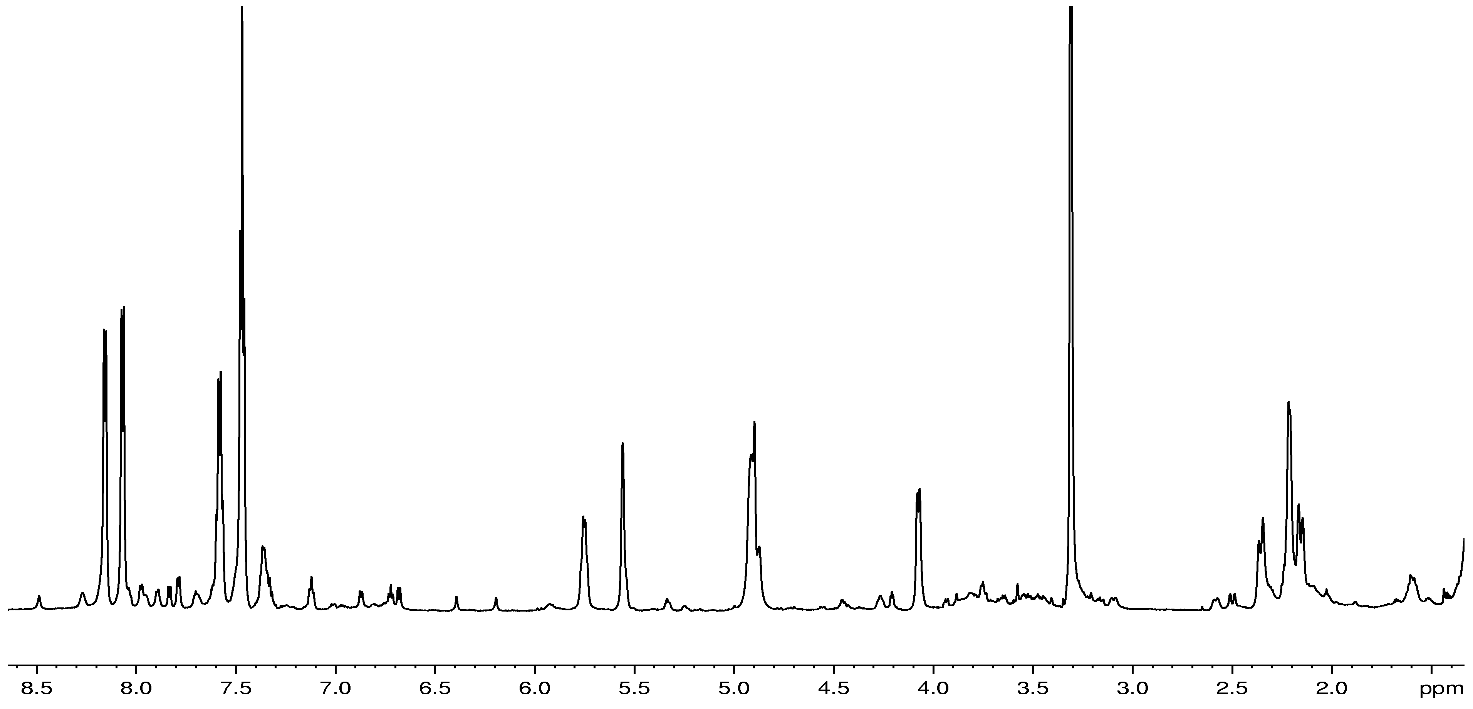


Figure 61: 3,5-O-bisbenzoyl quinic acid, ­^1^H-NMR spectrum (MeOH-d_3_)


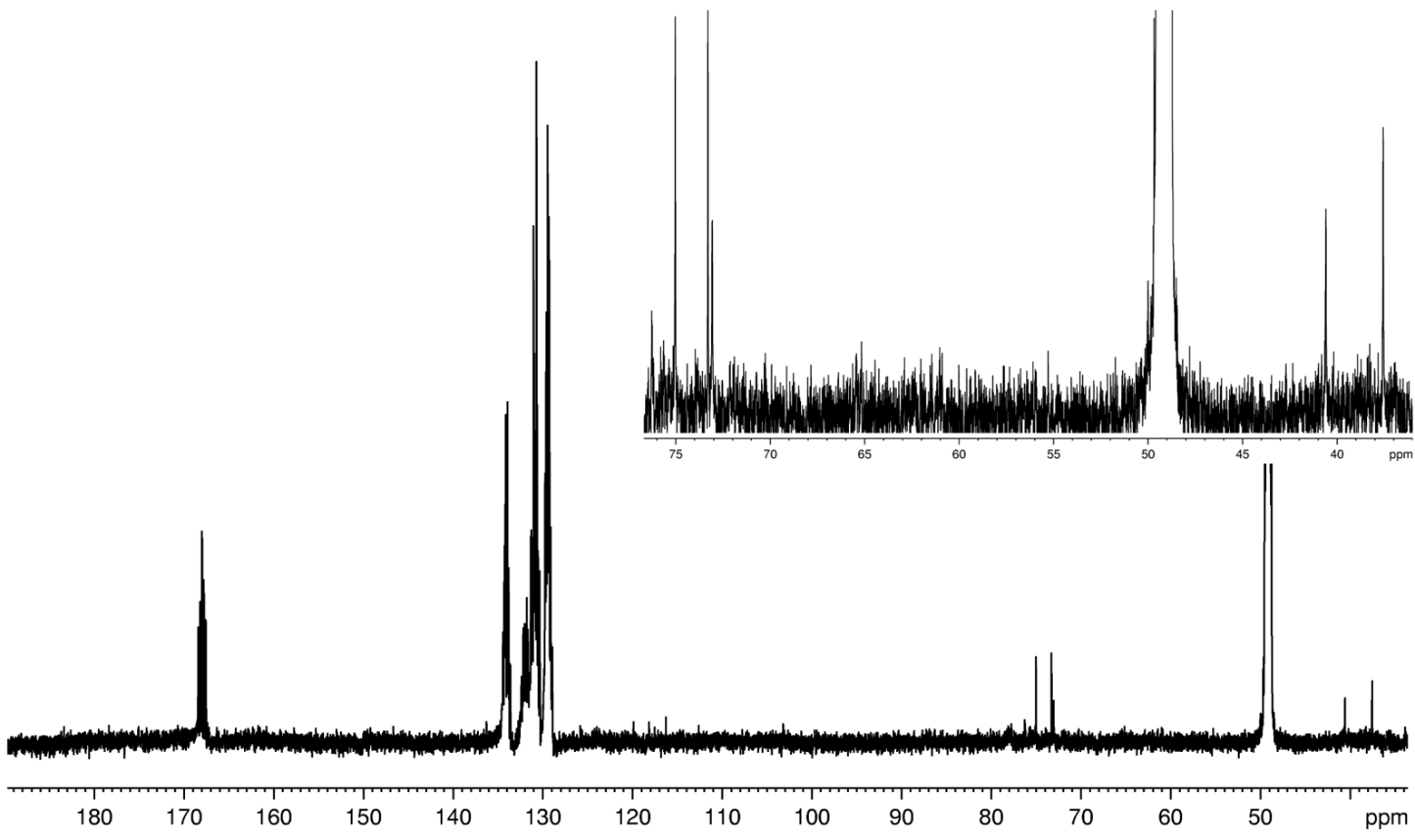


Figure 62: 3,5-O-bisbenzoyl quinic acid, ^13^C-NMR spectrum (MeOH-d_3_)


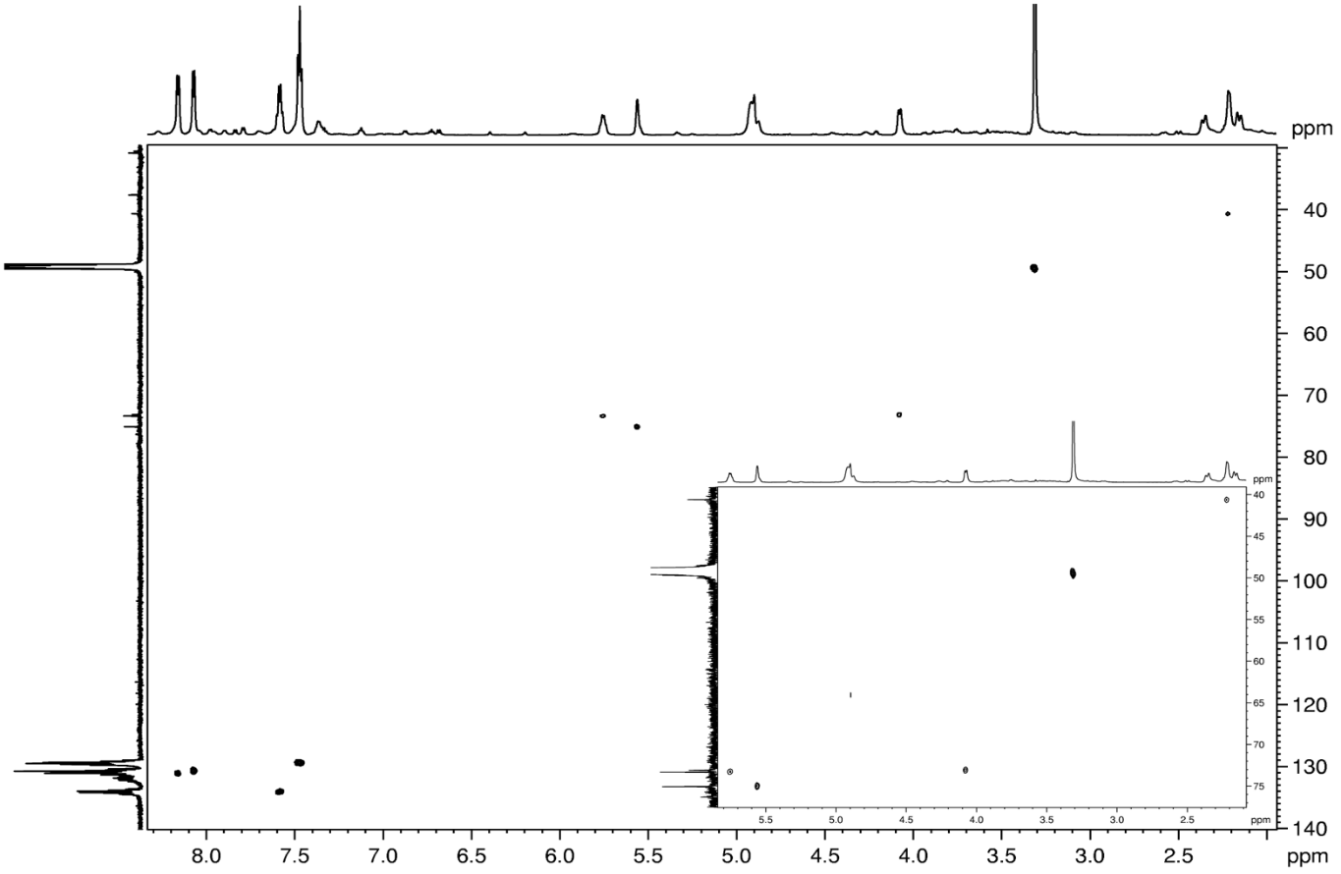


Figure 63: 3,5-O-bisbenzoyl quinic acid, HSQC spectrum (MeOH-d_3_)


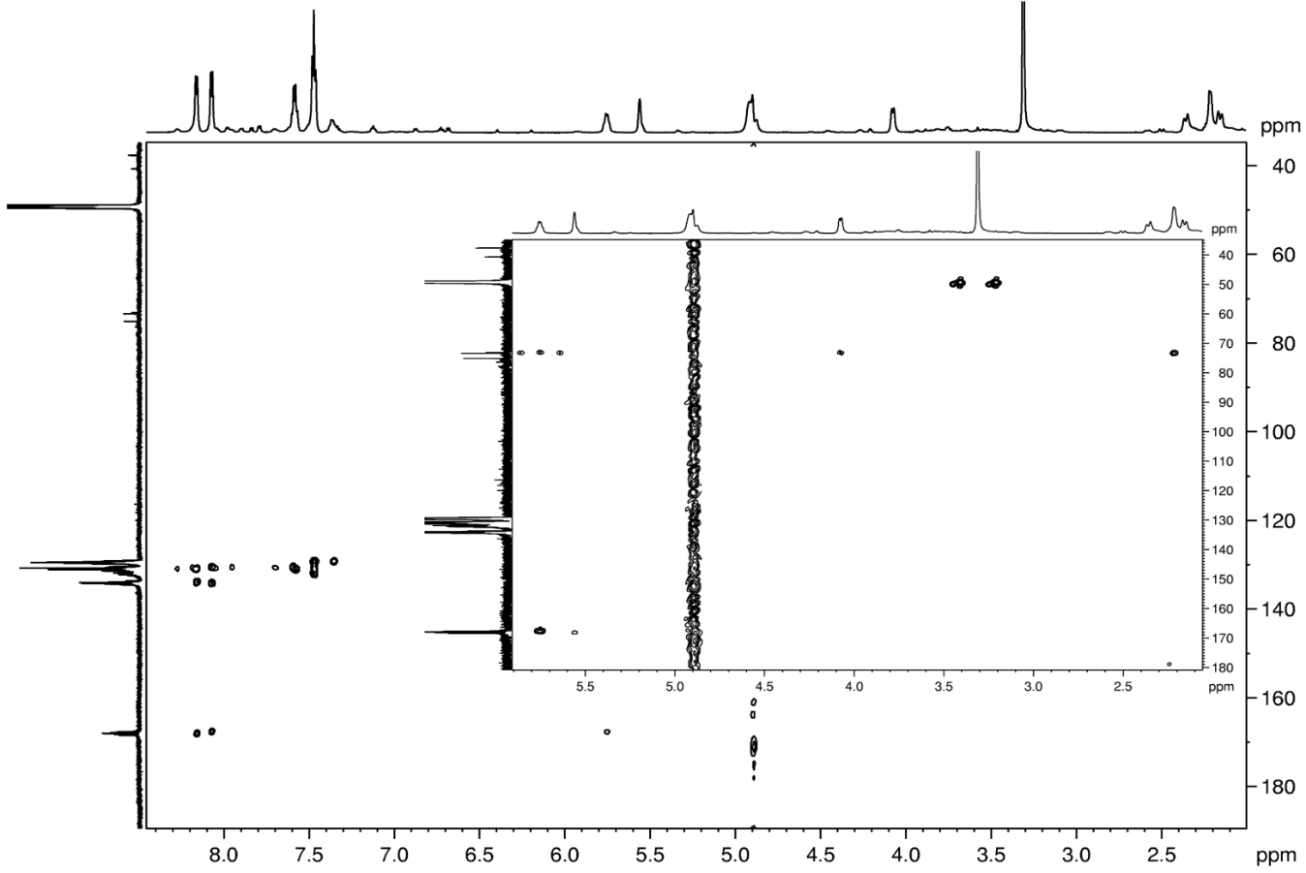


Figure 64: 3,5-O-bisbenzoyl quinic acid, HMBC spectrum (MeOH-d_3_)


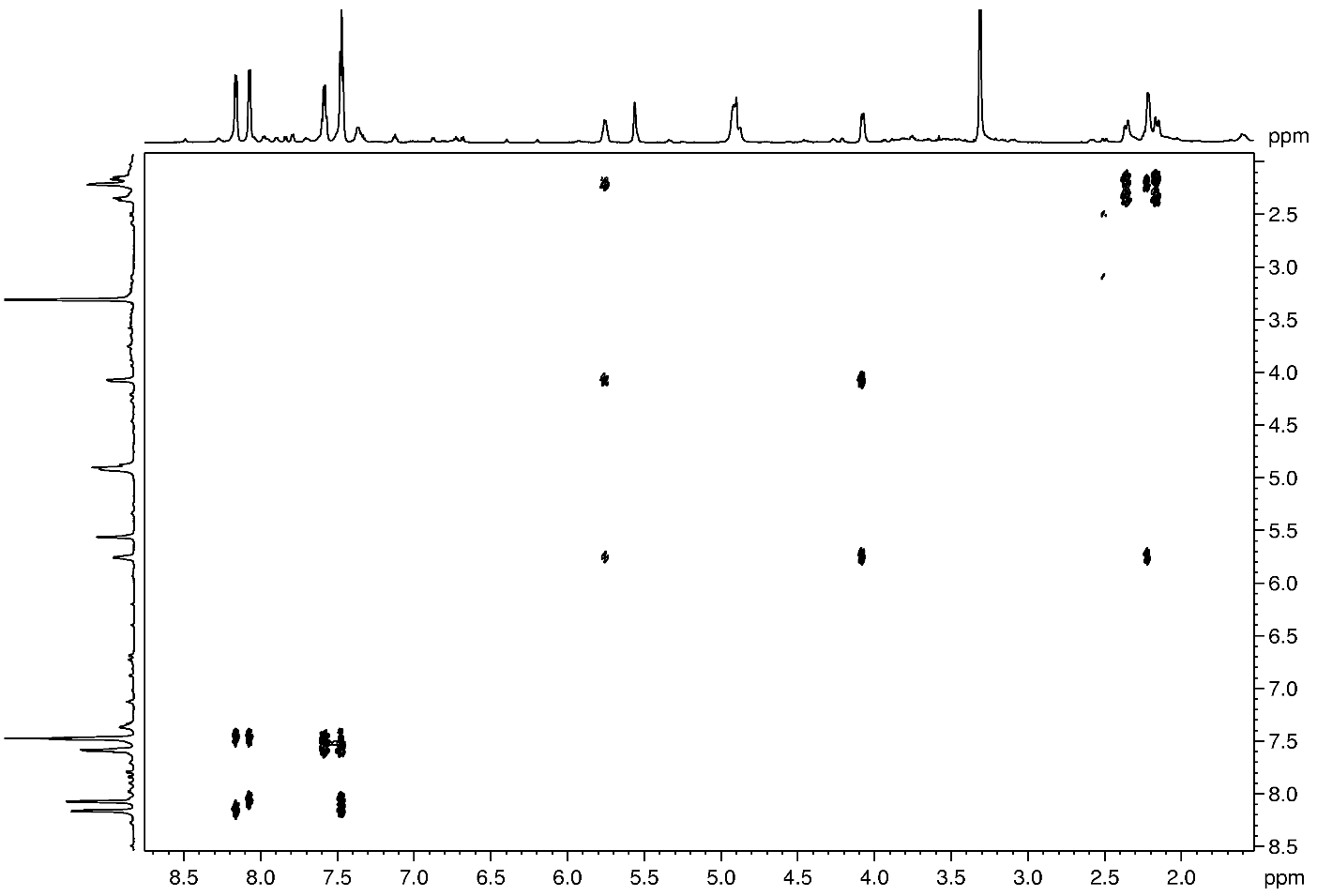


Figure 65: 3,5-O-bisbenzoyl quinic acid, ^1^H-^1^H COSY spectrum (MeOH-d_3_)

## 2.4 4,5-*O*-Bisbenzoylquinic acid


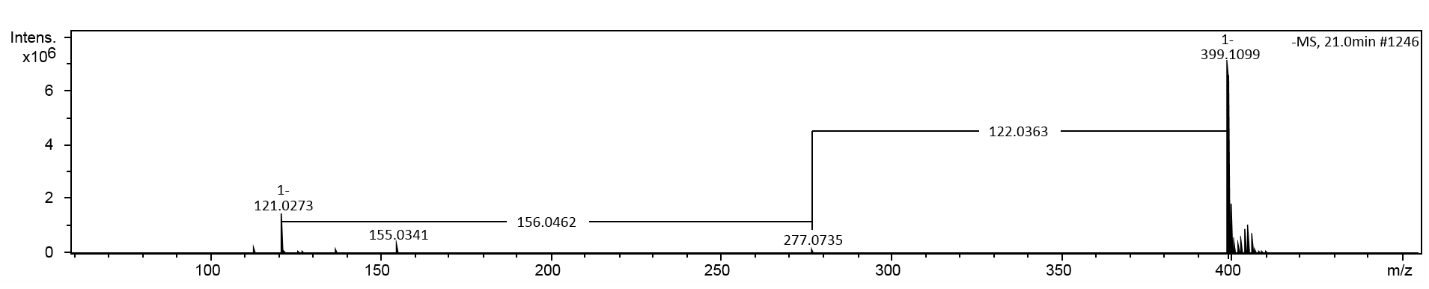


Figure 66: 4,5-O-bisbenzoyl quinic acid, HRESIMS spectrum, m/z 399.1090 [M-H]^-^


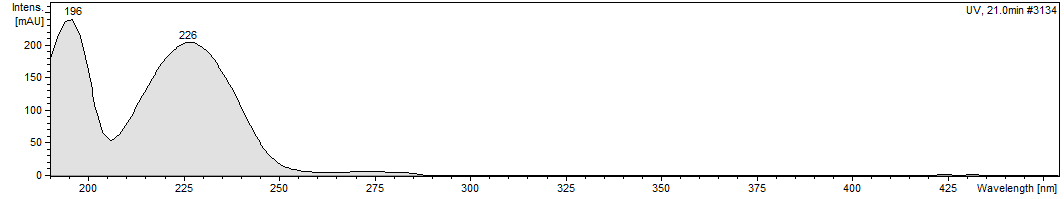


Figure 67: 4,5-O-bisbenzoyl quinic acid, UV spectrum from HPLC-DAD

Figure 68: 4,5-O-bisbenzoyl quinic acid, chemical structure with chemical shifts (MeOH-d_3_)


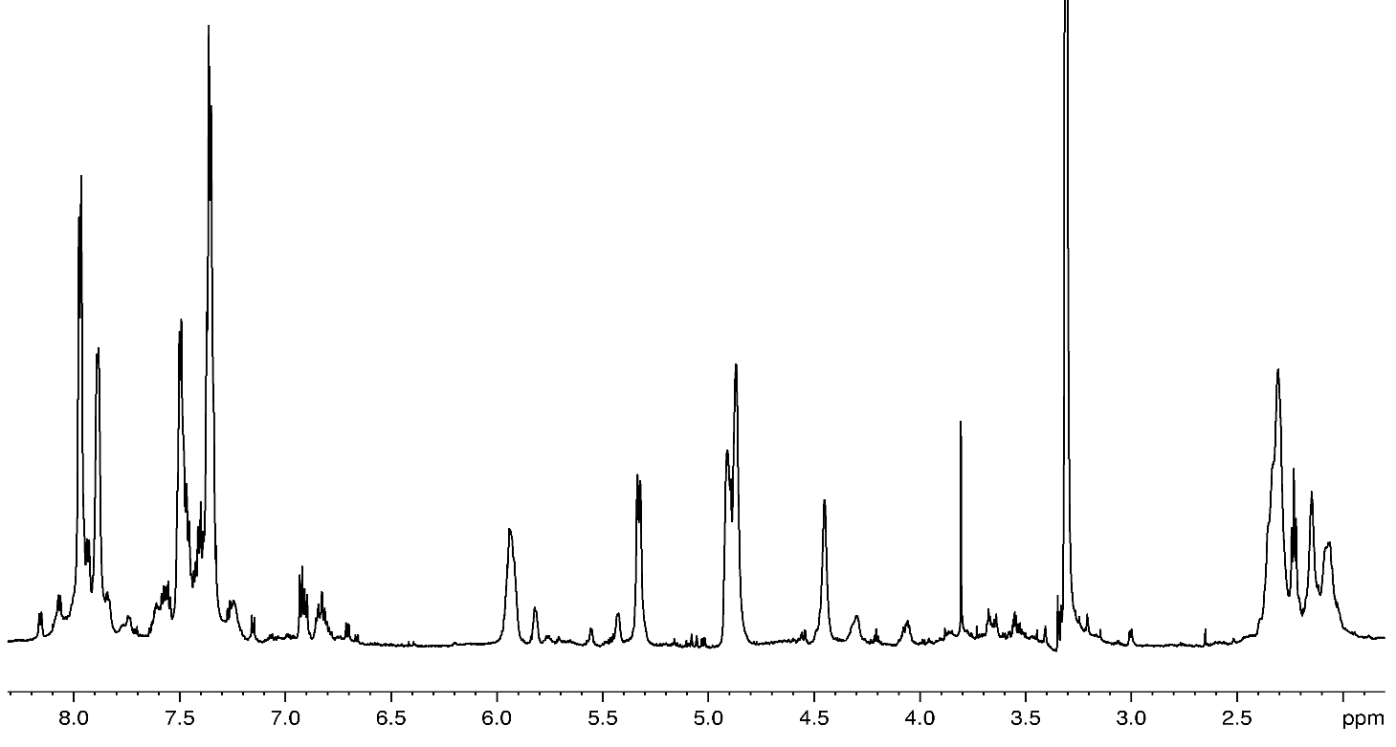


Figure 69: 4,5-O-bisbenzoyl quinic acid, ­^1^H-NMR spectrum (MeOH-d_3_)


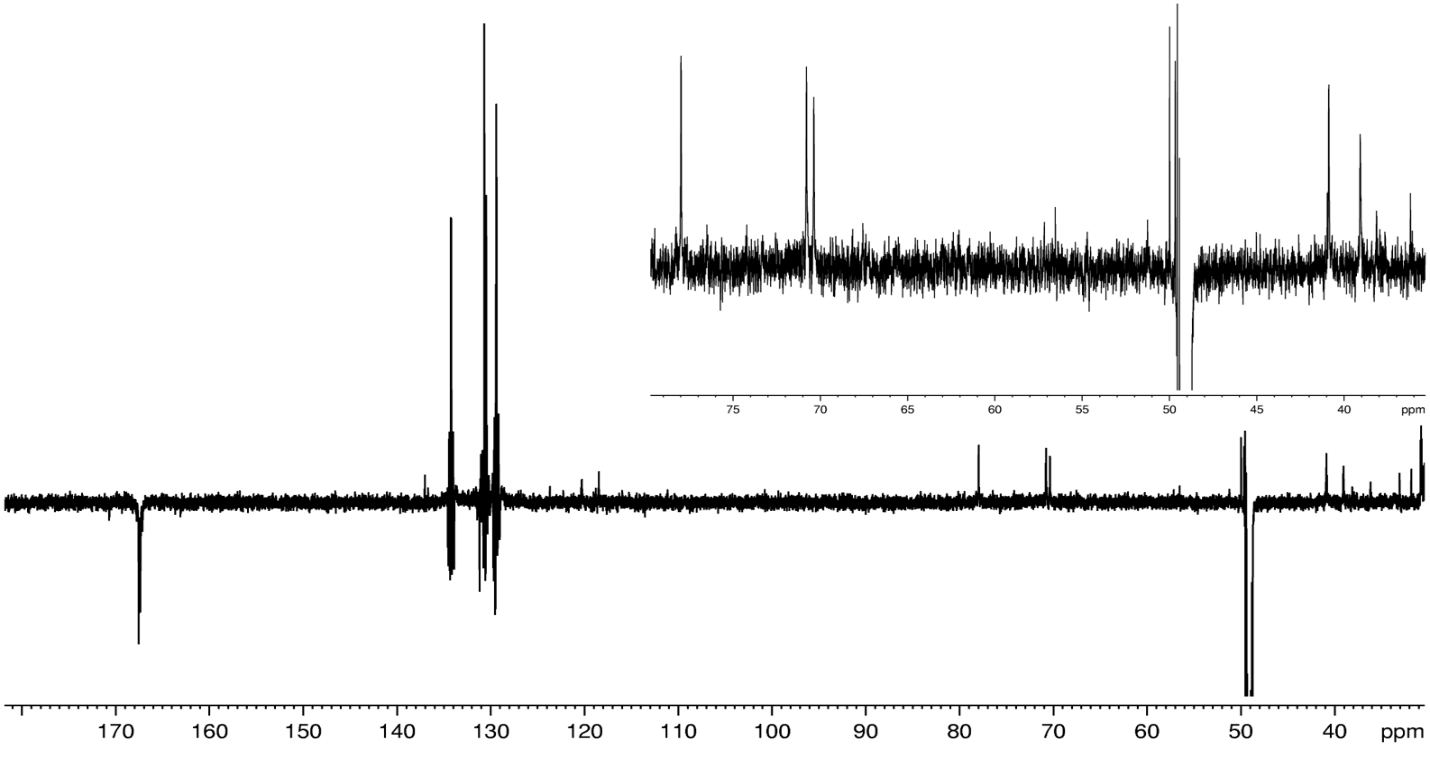


Figure 70: 4,5-O-bisbenzoyl quinic acid, DEPT spectrum (MeOH-d_3_)


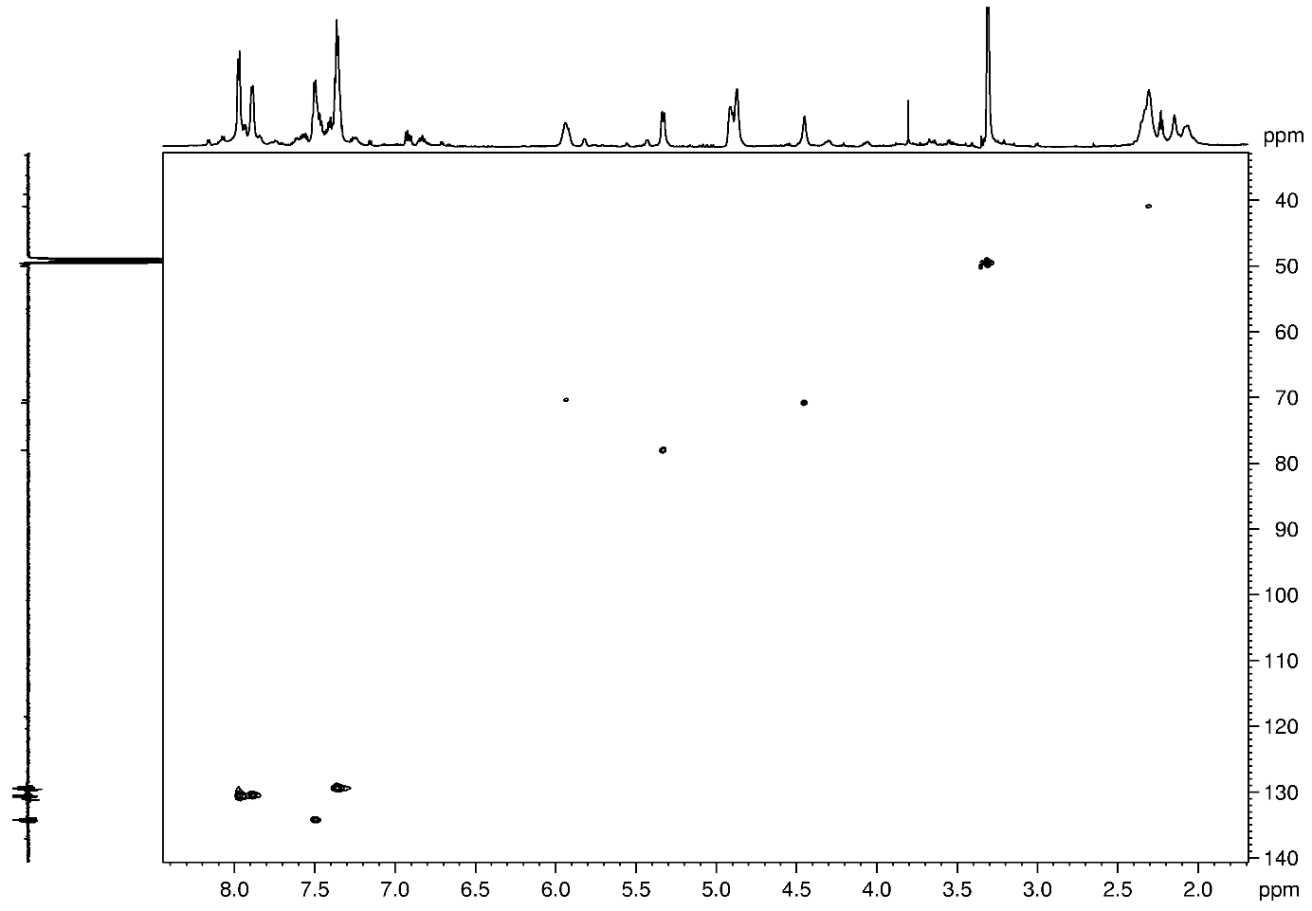


Figure 71: 4,5-O-bisbenzoyl quinic acid, HSQC spectrum (MeOH-d_3_)


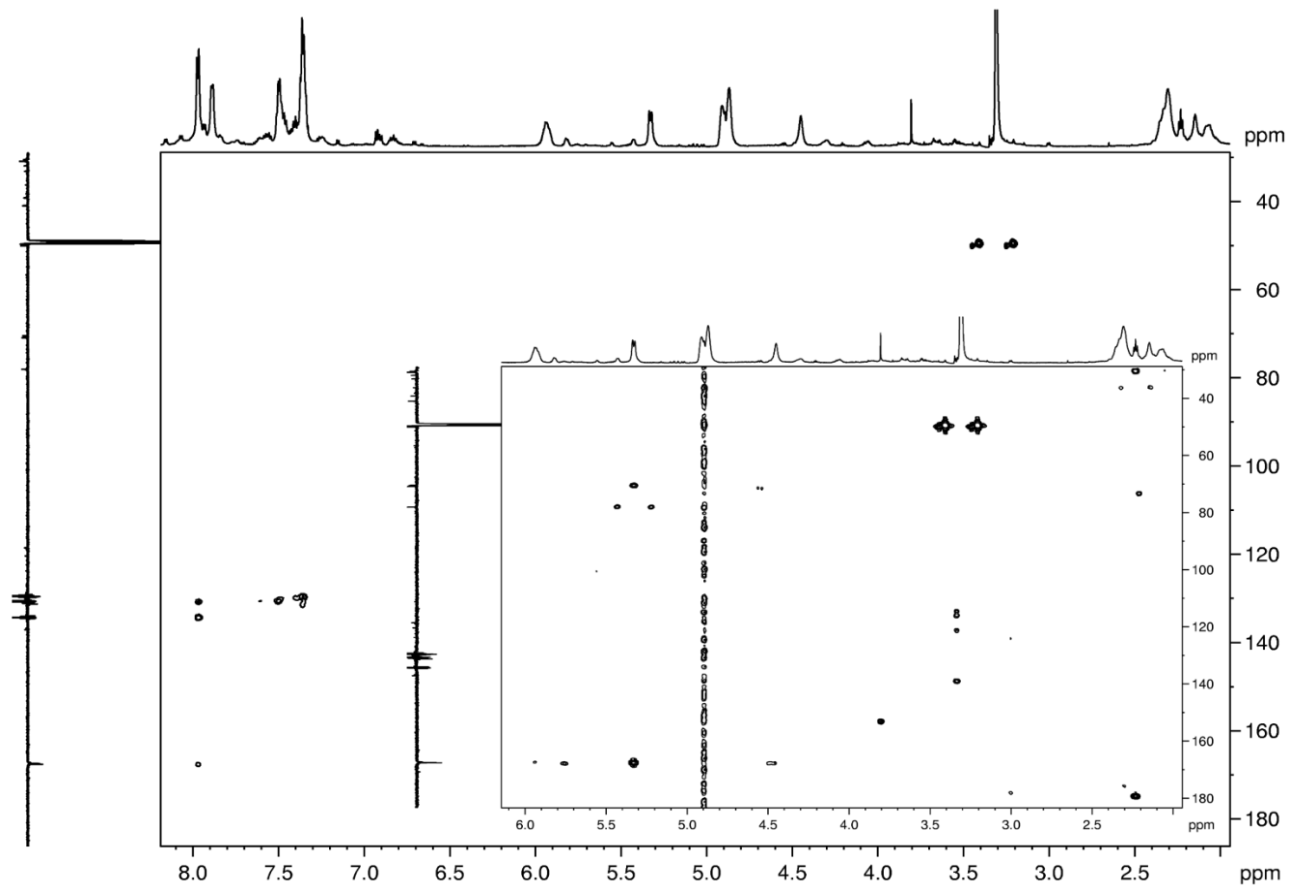


Figure 72: 4­,5-O-bisbenzoyl-quinic acid, HMBC spectrum (MeOH-d_3_)


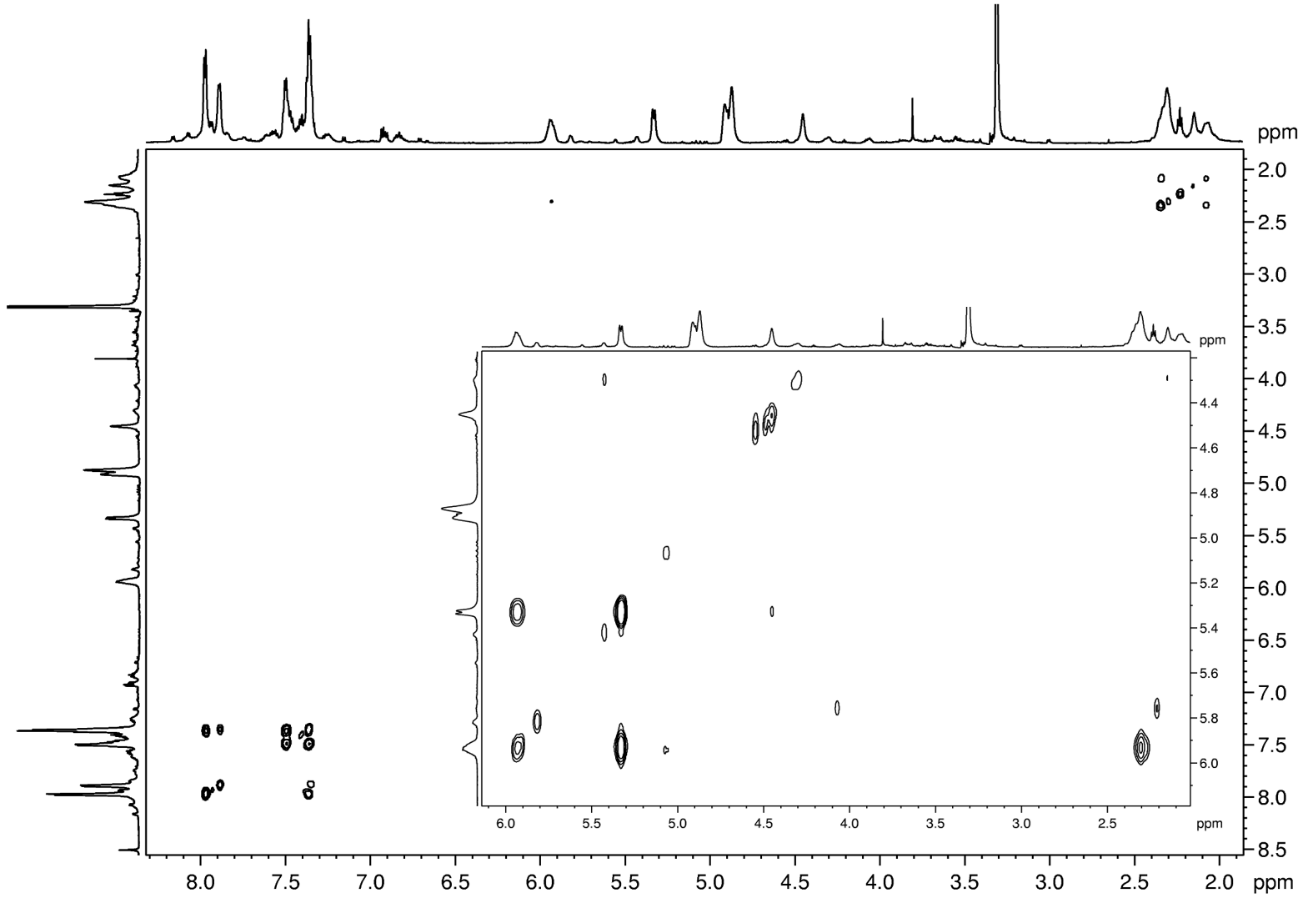


Figure 73: 4,5-O-bisbenzoyl-quinic acid, ^1^H-^1^H COSY spectrum (MeOH-d_3_)

## 2.5 2’,6’-*O*-Bisbenzoylsalicin

The title compound has a molecular formula of C_21_H_19_O_8_, as determined by HRESIMS ion at *m*/*z* 539.1548 [M-H+FA] ^-^ (calcd for C_28_H_27_O_11_, *m/z* 539.1559). The ^1^H-NMR spectrum indicated a glucosyl moiety with unshielded signals from H-1', H-2' and H-6' which were suggested to be substituted. The ^13^C-chemical shifts of the aromatic substituents attached positions 1, 3, and 6, C-2, C-1’’, and C-7’’’, were assigned from HMBC correlations. The substituents attached to positions 2' and 6' were benzoyl groups. ^1^H-NMR showed two partially overlapping sets of signals corresponding to phenyl rings, indicating that the glucosyl moiety was 2',6'-bisbenzoylated. Based on the coupling pattern of the remaining signals in the aromatic part of the ^1^H-NMR spectrum, the third aromatic substituent at position 1' was identified as saligenin. Because of the high ^13^C-labeling of the compound, the carbon backbone of both the glucosyl and saligenin moieties was elucidated from ^13^C-^13^C COSY correlations. All ^13^C-chemical shifts were extracted from HSQC or HMBC data and the title compound was identified as 2',6'-O-bisbenzoylsalicin.


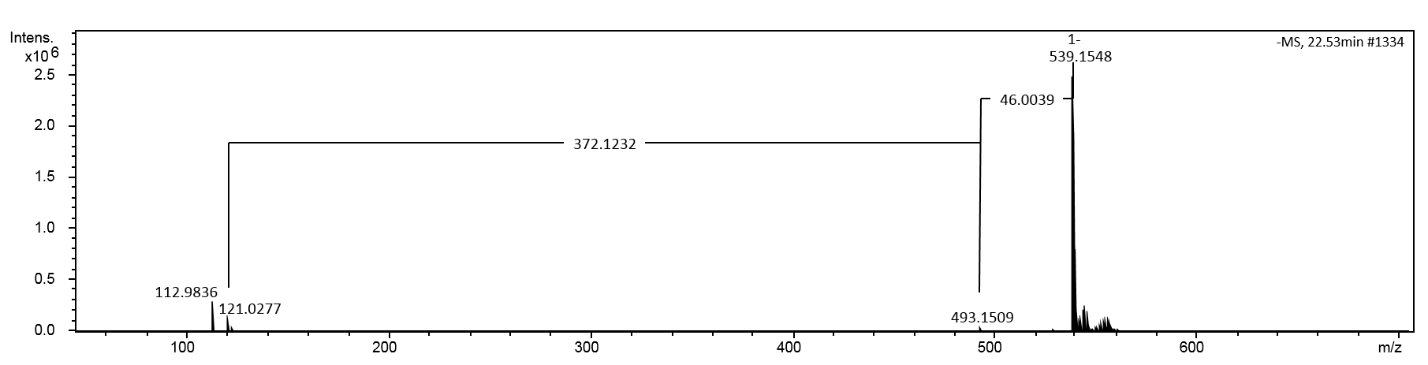


Figure 74: 2’,6’-O-bisbenzoylsalicin, HRESIMS spectrum, m/z 539.1548 [M-H+FA]^-^


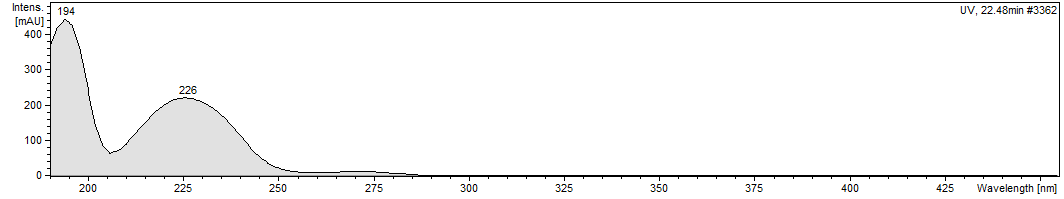


Figure 75: 2’,6’-O-bisbenzoylsalicin, UV spectrum from HPLC-DAD

Figure 76: 2’,6’-O-bisbenzoylsalicin, structure with chemical shifts (MeOH-d_3_)


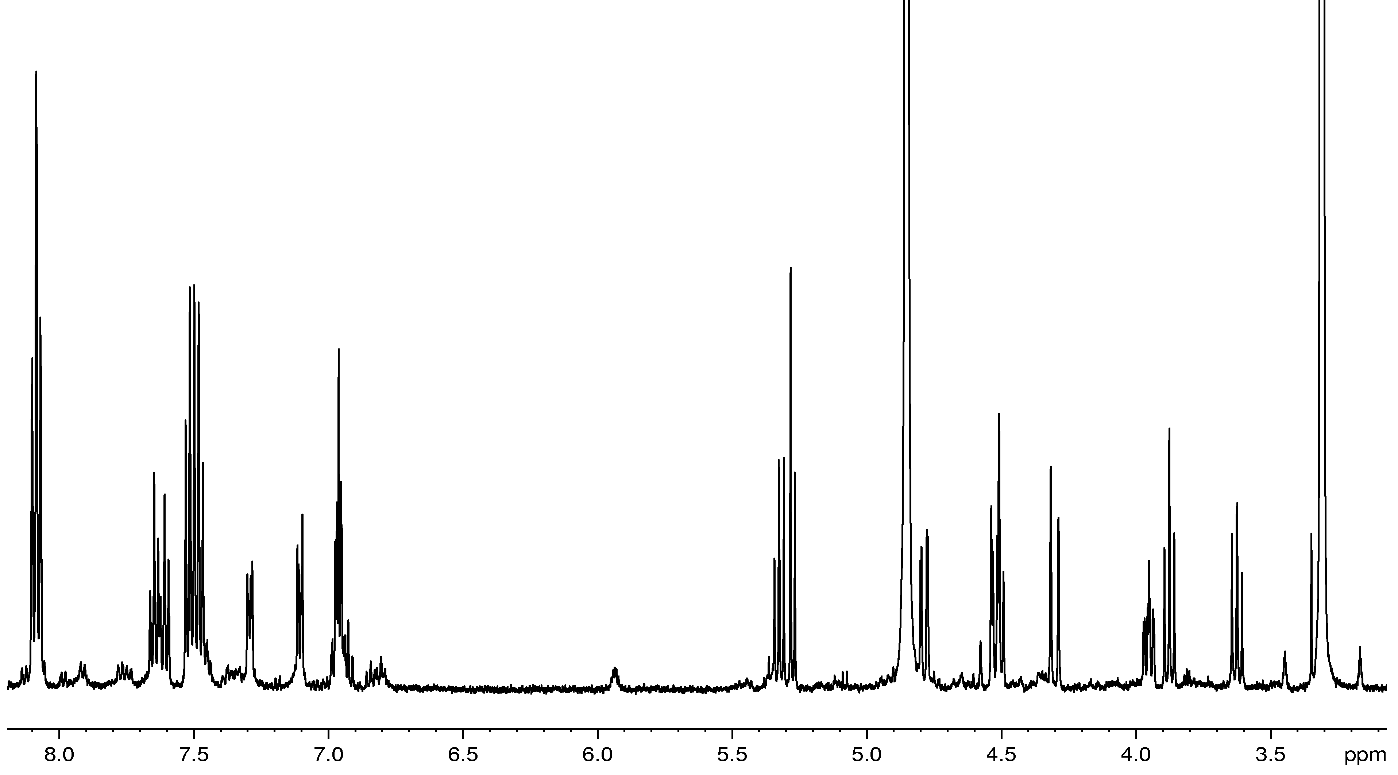


Figure 77: 2’,6’-O-bisbenzoylsalicin, ­^1^H-NMR spectrum (MeOH-d_3_)


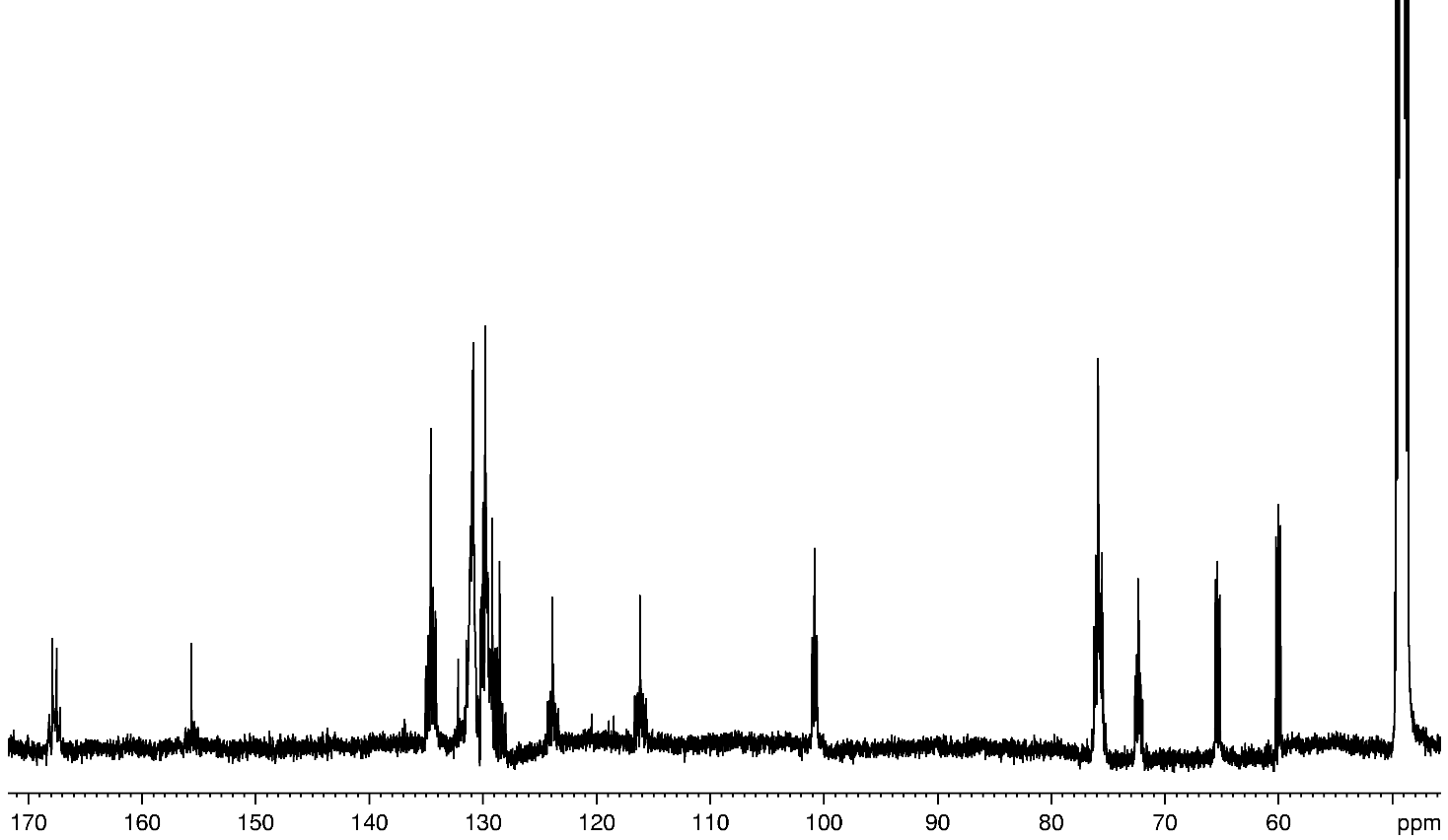


Figure 78: 2’,6’-O-bisbenzoylsalicin, ^13^C-NMR spectrum (MeOH-d_3_)


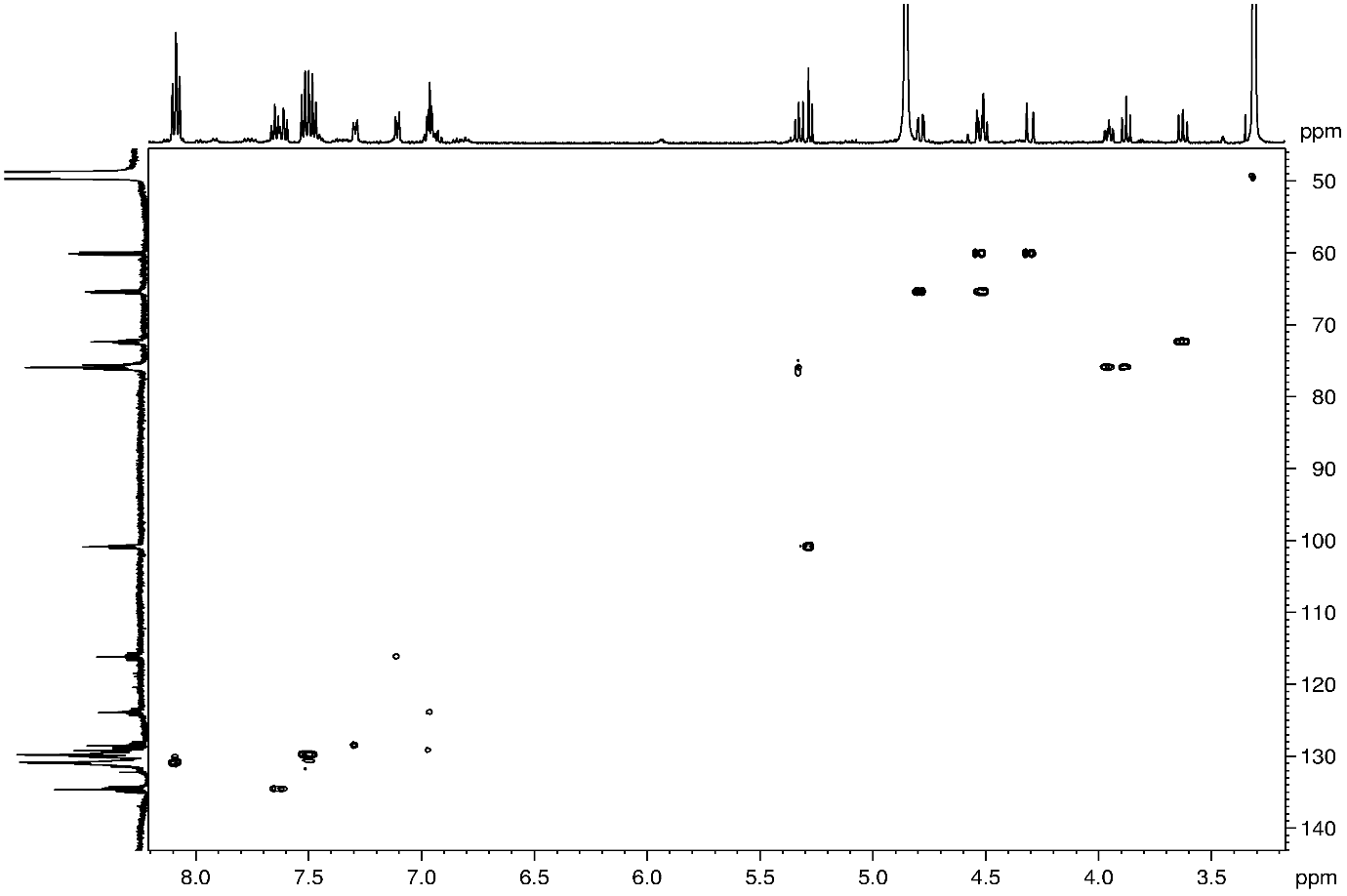


Figure 79: 2’6’-O-bisbenzoylsalicin, HSQC spectrum (MeOH-d_3_)


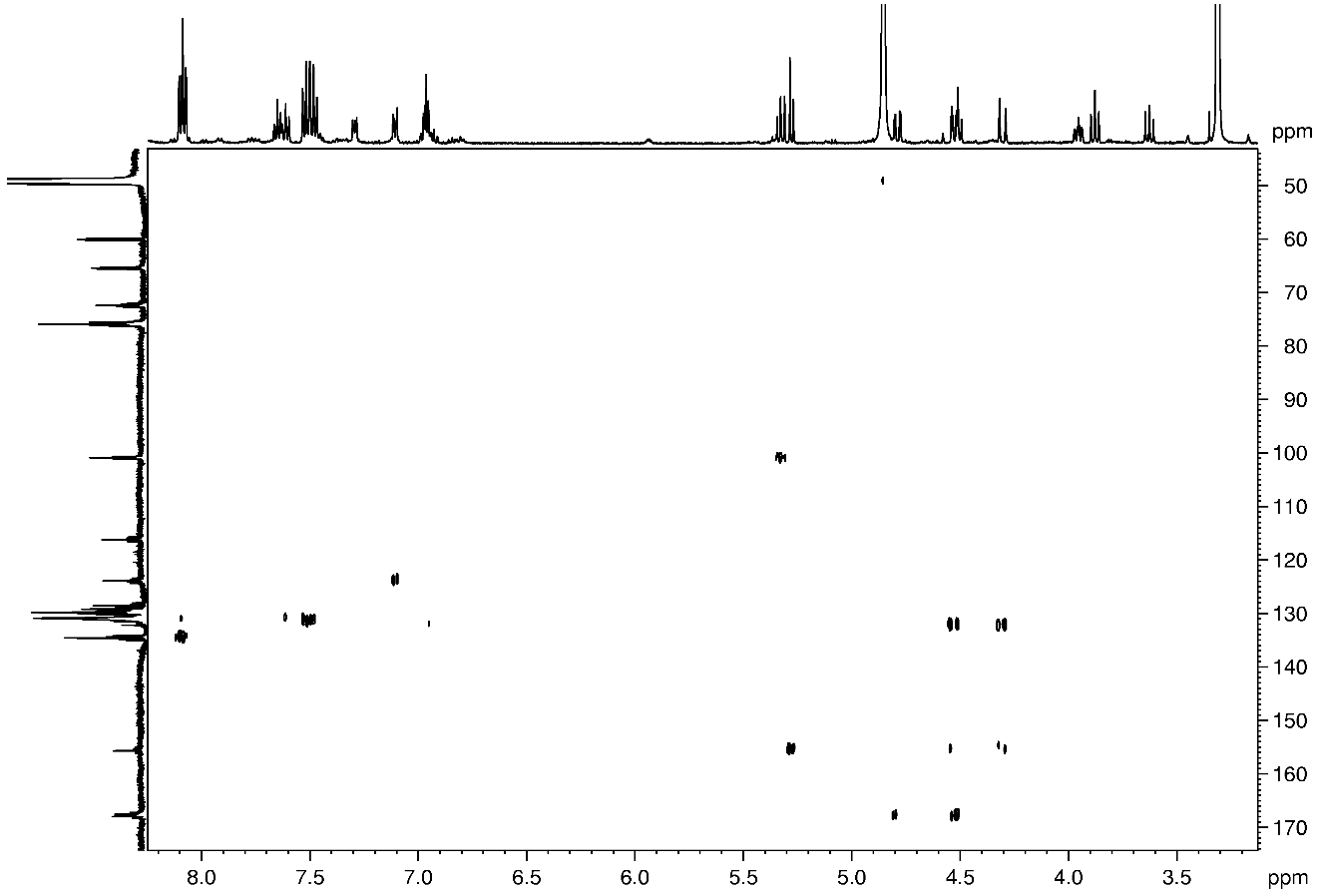


Figure 80: 2’6’-O-bisbenzoylsalicin, HMBC spectrum (MeOH-d_3_)


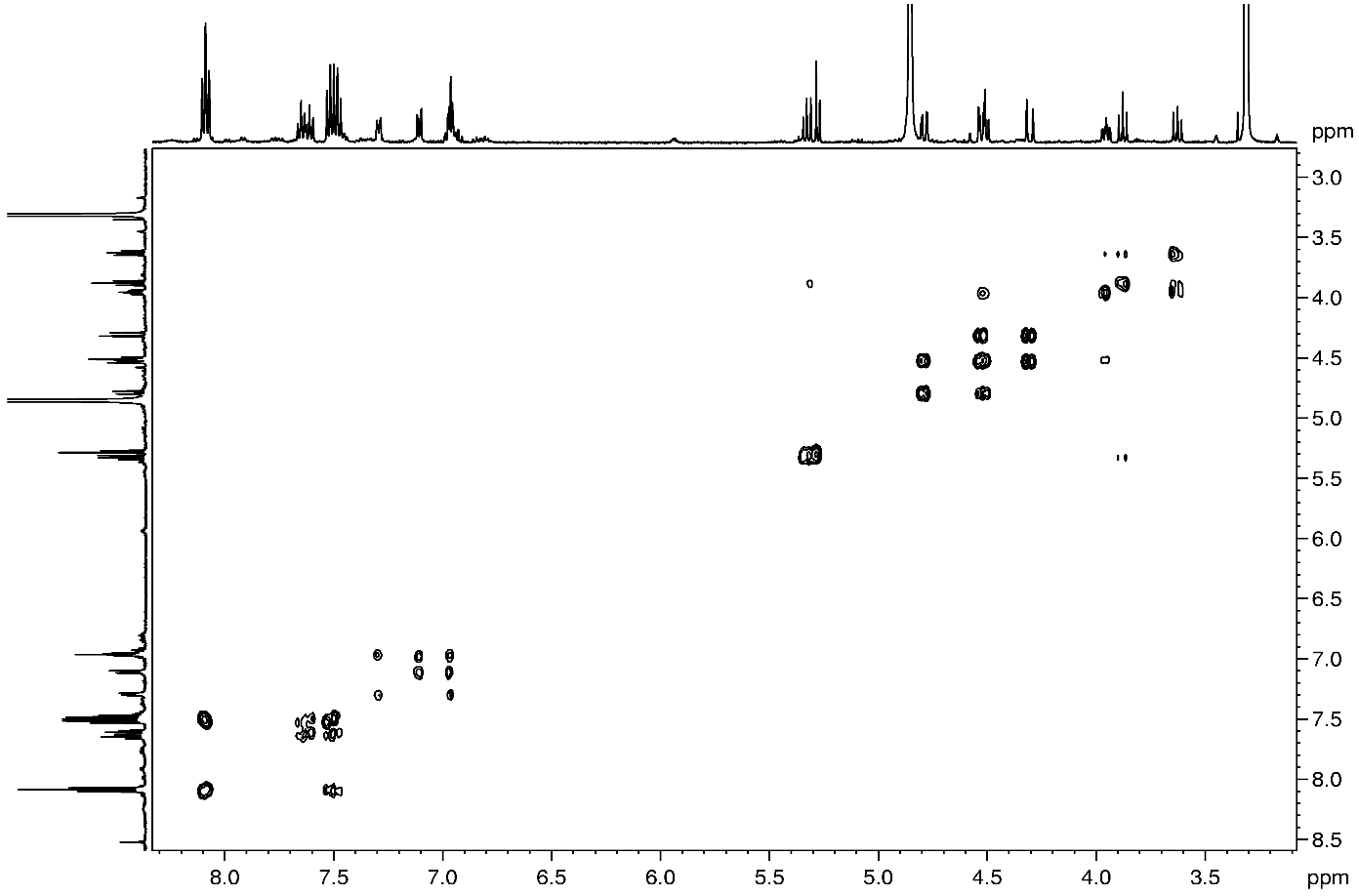


Figure 81: 2’,6’-O-bisbenzoylsalicin, ^1^H-^1^H COSY spectrum (MeOH-d_3_)


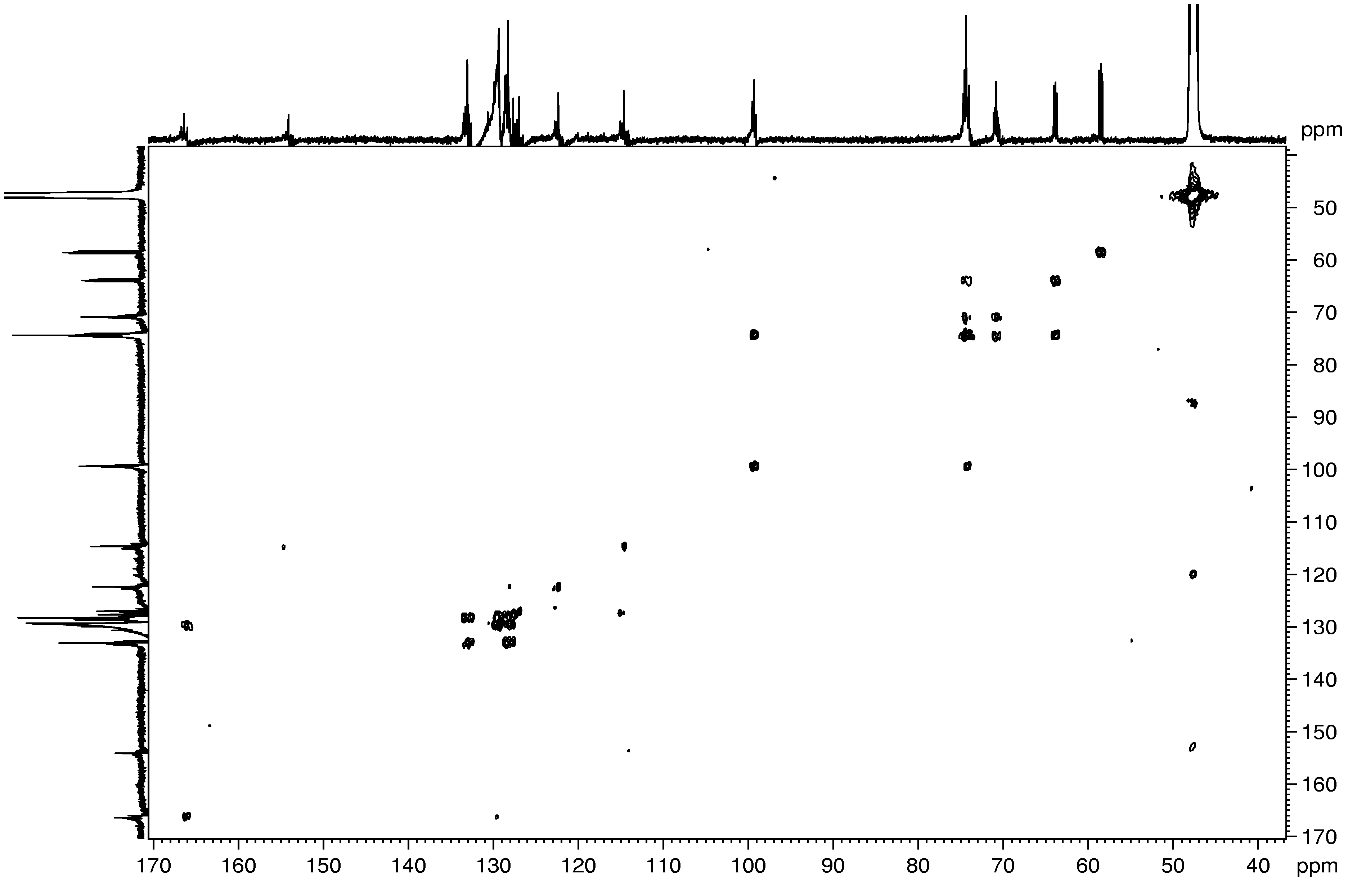


Figure 82: 2’,6’-O-bisbenzoylsalicin, ^13^C-^13^C COSY spectrum (MeOH-d_3_)

## 2.6 Catechol glucoside


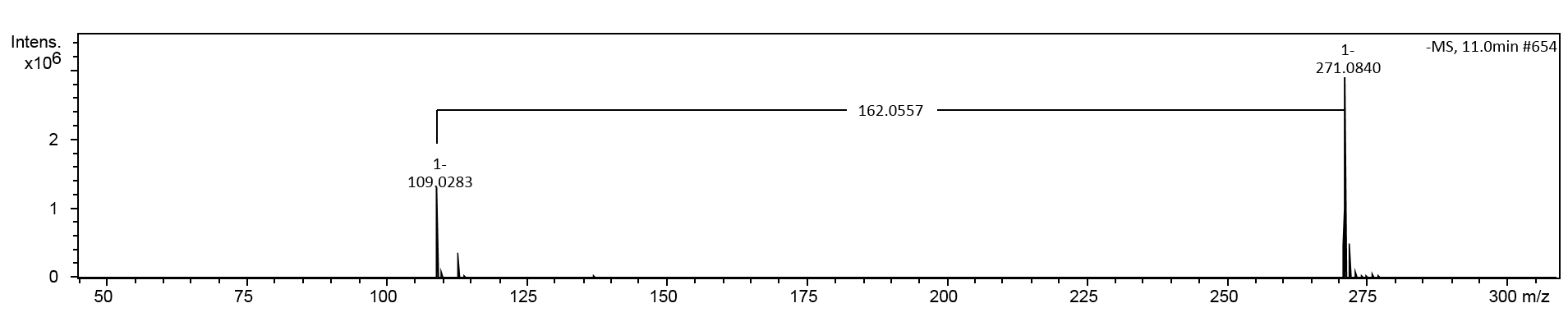


Figure 83: Catechol glucoside, HRESIMS spectrum, m/z 271.0840 [M-H]^-^


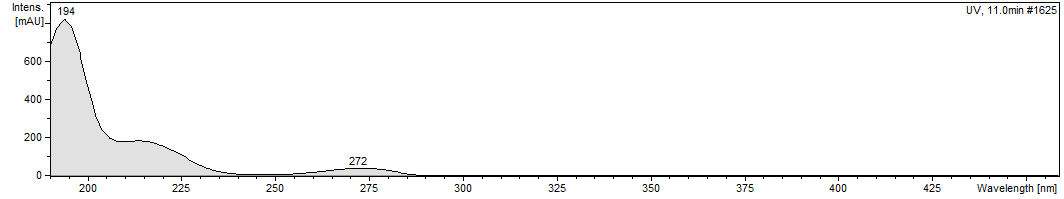


Figure 84: Catechol glucoside, UV spectrum from HPLC-DAD

Figure 85: Catechol glucoside, structure with chemical shifts (MeOH-d_3_), the acquired analytical data is in accordance with previous literature (Itoh et al. 2000)


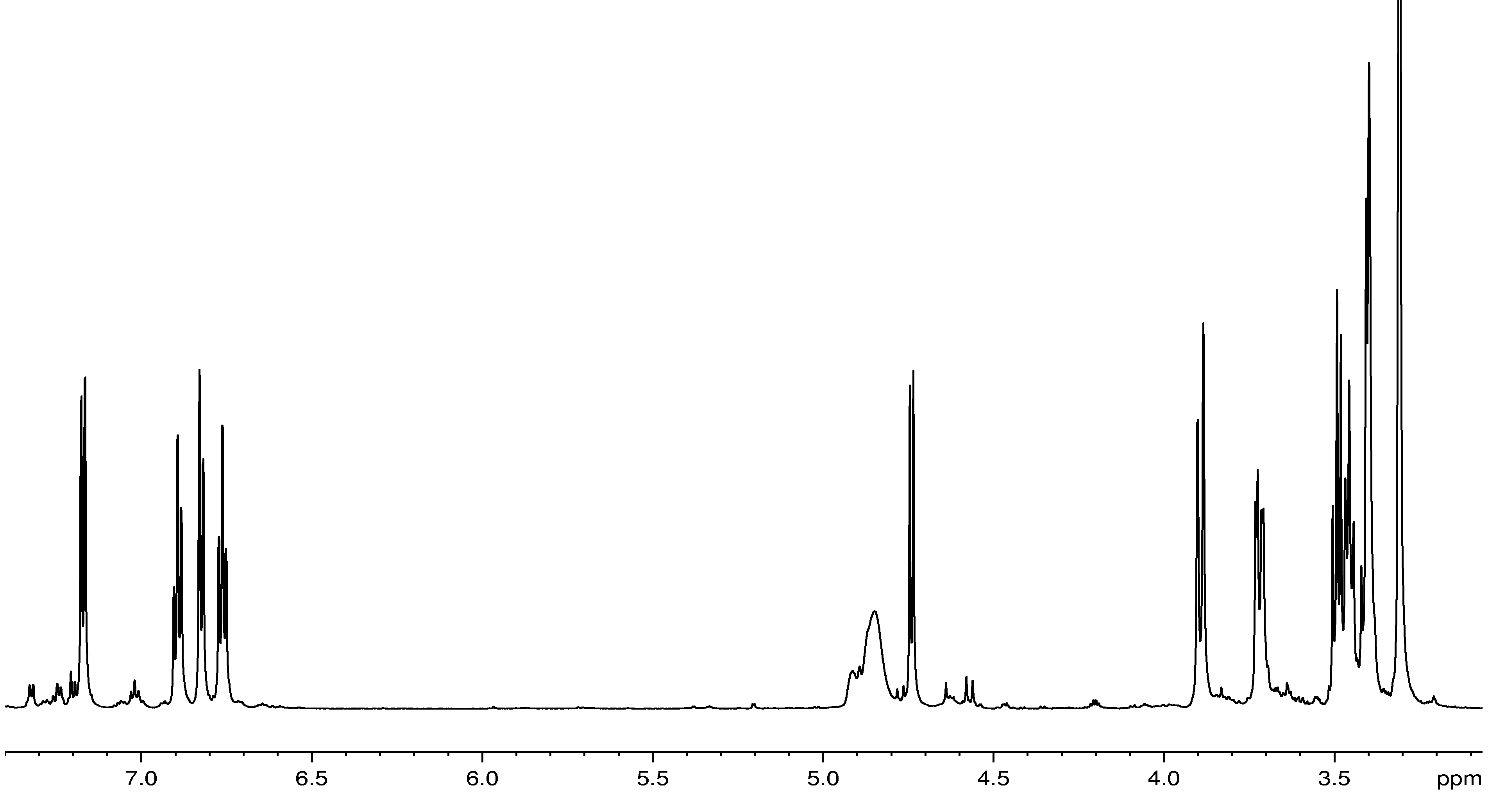


Figure 86: Catechol glucoside, ­^1^H-NMR spectrum (MeOH-d_3_)


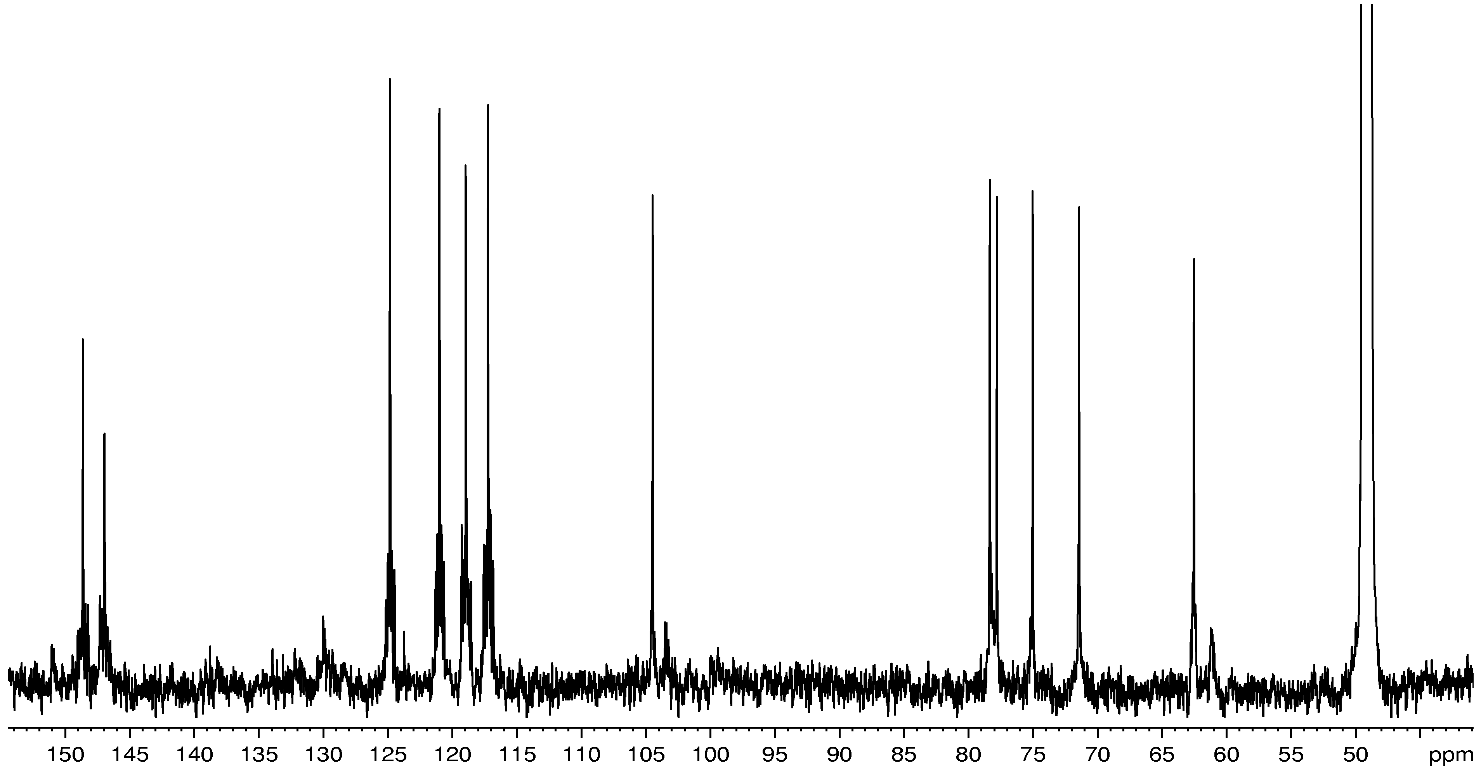


Figure 87: Catechol glucoside, ^13^C-NMR spectrum (MeOH-d_3_)


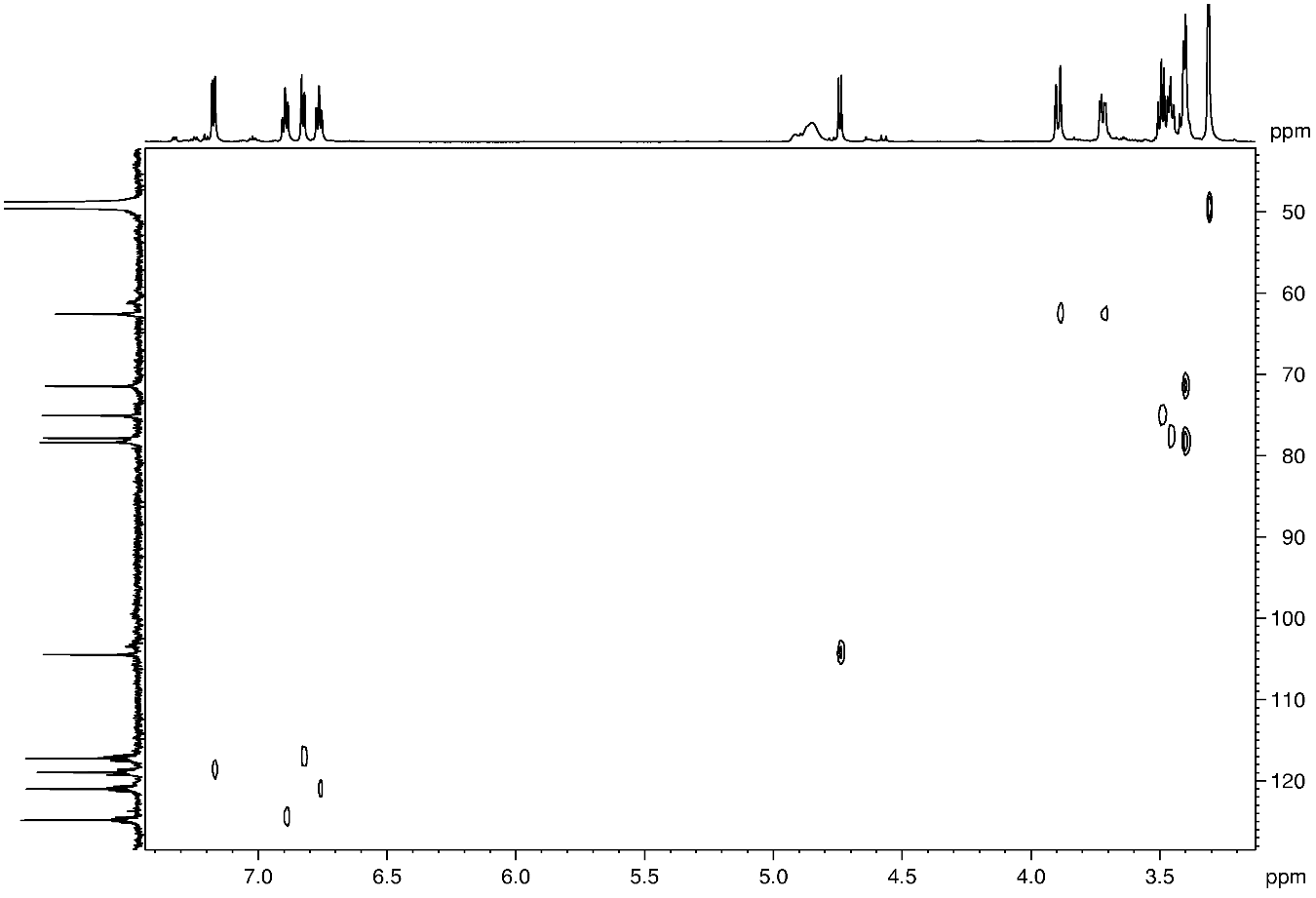


Figure 88: Catechol glucoside, HSQC spectrum (MeOH-d_3_)


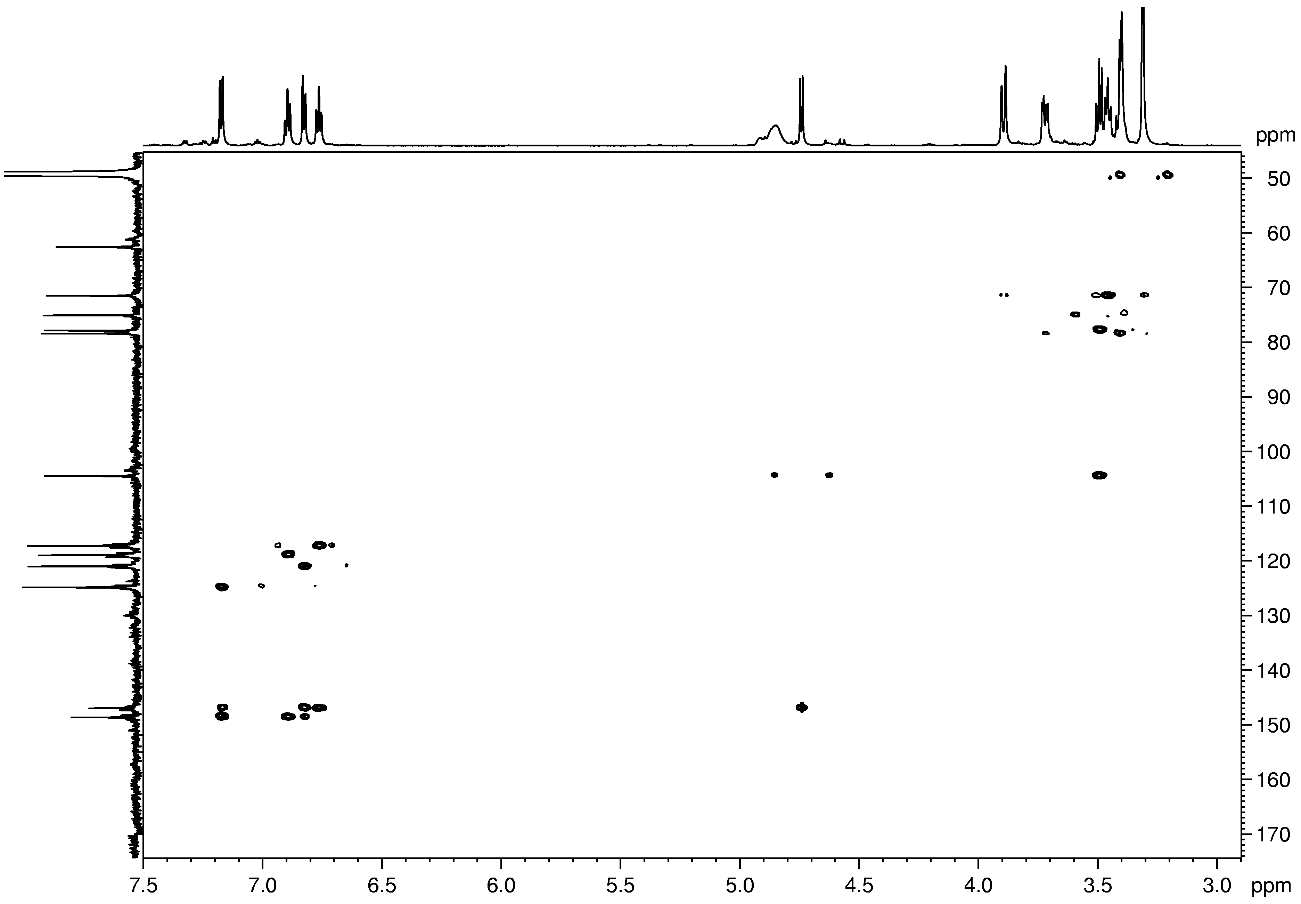


Figure 89: Catechol glucoside, HMBC spectrum (MeOH-d_3_)


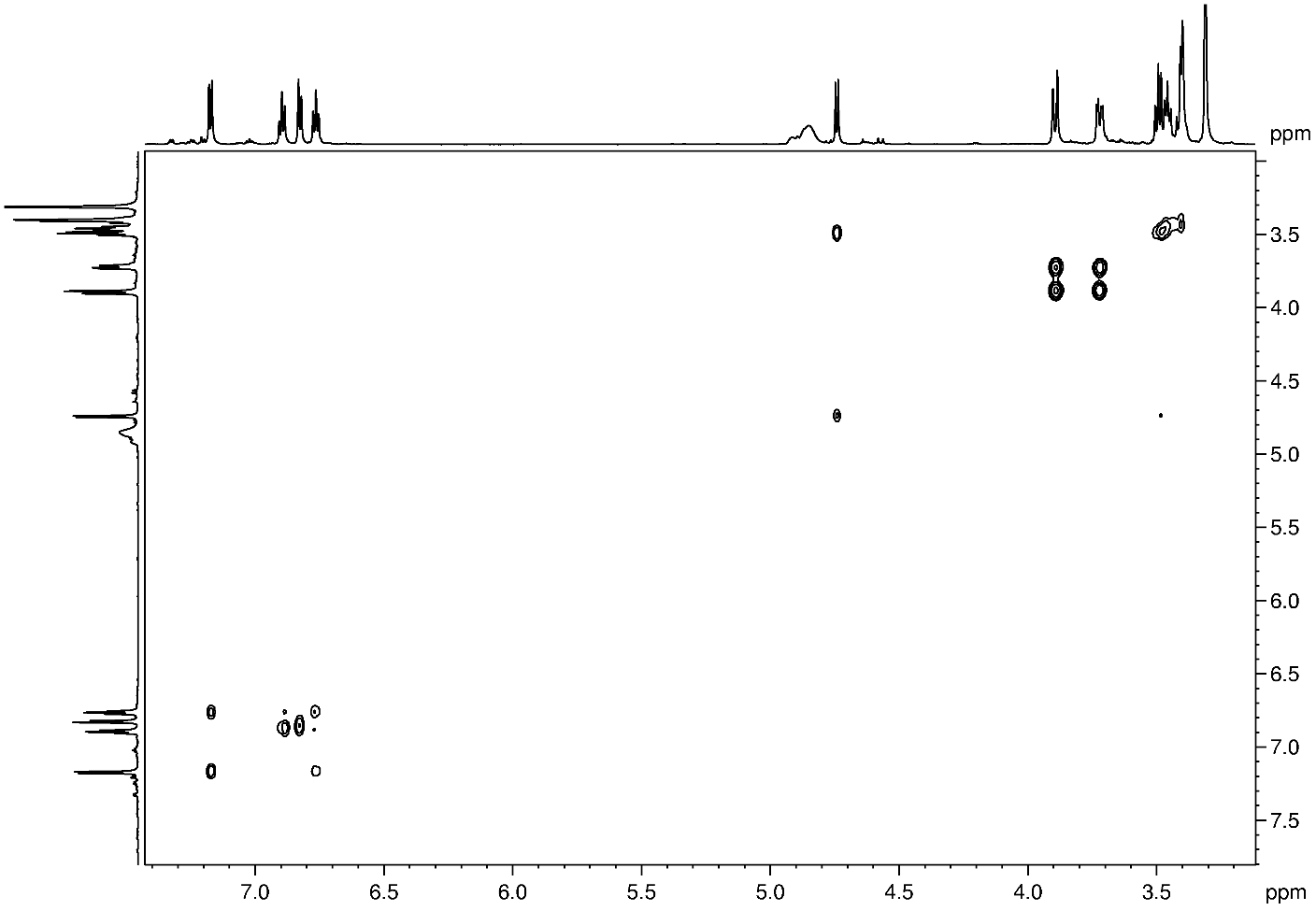


Figure 90: Catechol glucoside, ^1^H-^1^H COSY spectrum (MeOH-d_3_)

## 2.7 Isosalicin


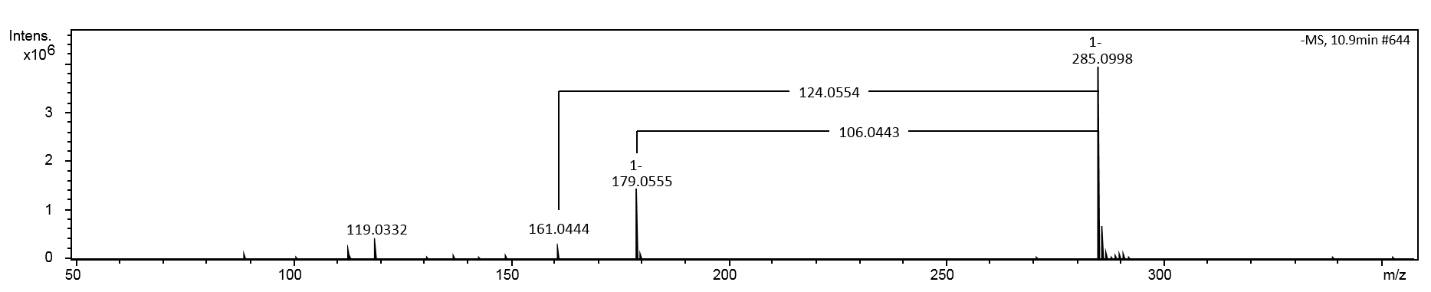


Figure 91: Isosalicin, HRESIMS spectrum, m/z 285.0998 [M-H]^-^


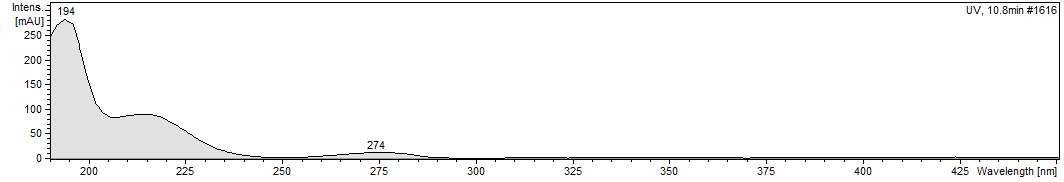


Figure 92: Isosalicin, UV spectrum from HPLC-DAD

Figure 93: Isosalicin, structure with chemical shifts (MeOH-d_3_), the acquired analytical data is in accordance with previous literature (Kanho et al. 2005)


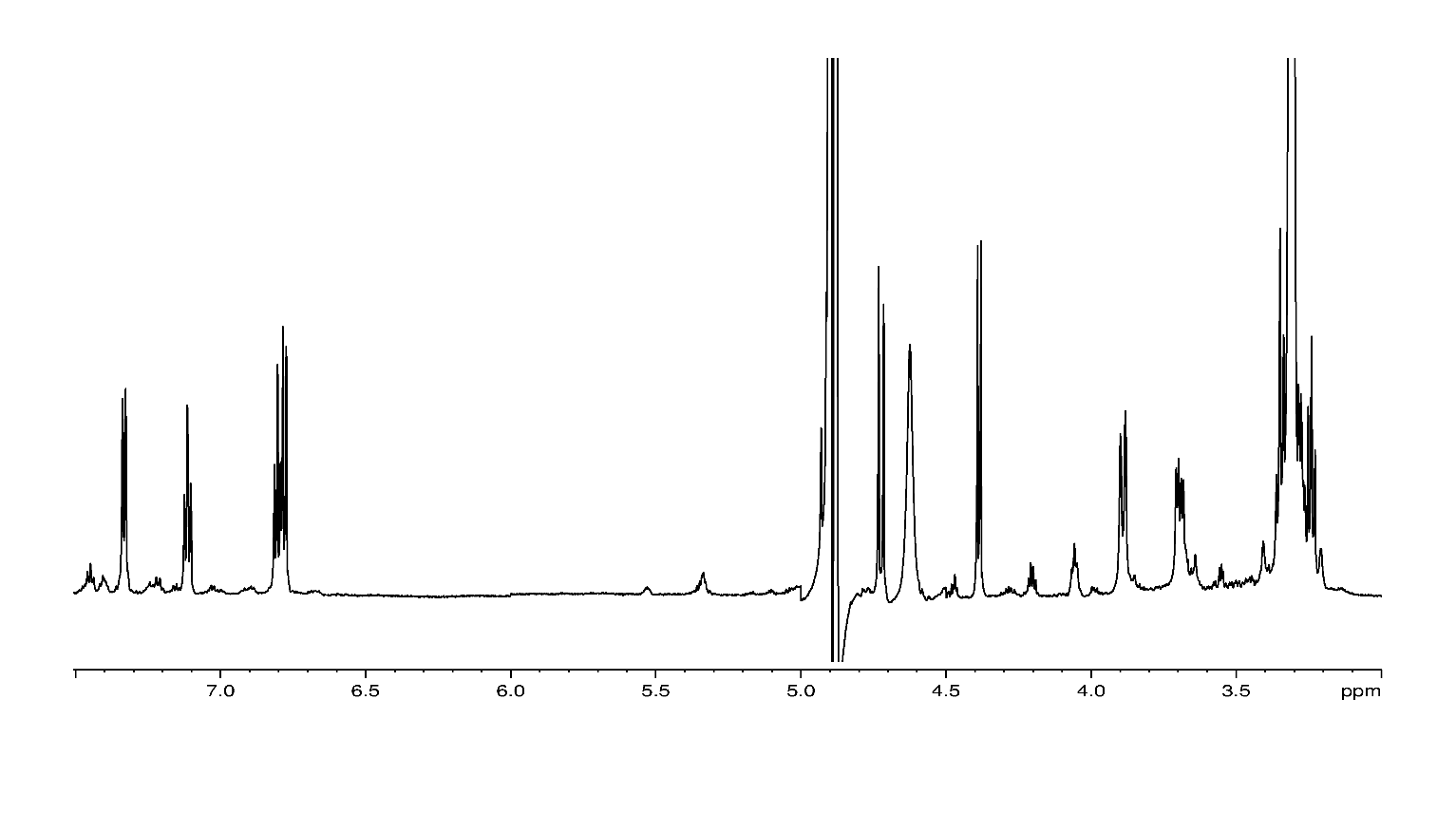


Figure 94: Isosalicin, ­^1^H-NMR spectrum (MeOH-d_3_)

Figure 95: Isosalicin, ^13^C-NMR spectrum (MeOH-d_3_)

Figure 96: Isosalicin, HSQC spectrum (MeOH-d_3_)

Figure 97: Isosalicin, HMBC spectrum (MeOH-d_3_)

## 2.8 Populin

Figure 98: Populin, HRESIMS spectrum, m/z 435.1310 [M-H+FA]^-^

Figure 99: Populin, UV spectrum from HPLC-DAD

Figure 100: Populin, structure with chemical shifts (MeOH-d_3_), the acquired analytical data is in accordance with previous literature (Dommisse et al. 1986)

Figure 101: Populin, ­^1^H-NMR spectrum (MeOH-d_3_)

Figure 102: Populin, ^13^C-NMR spectrum (MeOH-d_3_)

Figure 103: Populin, HSQC spectrum (MeOH-d_3_)

Figure 104: Populin, ^1^H-^1^H COSY spectrum (MeOH-d_3_)

## Nigracin

Figure 105: Nigracin, HRESIMS spectrum, m/z 451.1269 [M-H+FA]^-^

Figure 106: Nigracin, UV spectrum from HPLC-DAD

Figure 107: Nigracin, structure with chemical shifts (MeOH-d_3_), structure with chemical shifts (MeOH-d_3_), the acquired analytical data is in accordance with previous literature (Sashidhara et al. 2014)

Figure 108: Nigracin, ­^1^H-NMR spectrum (MeOH-d_3_)

Figure 109: Nigracin, HSQC spectrum (MeOH-d_3_)

Figure 110: Nigracin, HMBC spectrum (MeOH-d_3_)

## 2.10 3-*O*-glycosyl-2’-*O*-xylosyl-6’-*O*-rhamnosyl-quercetin

Figure 111: 3-O-glycosyl-2’-O-xylosyl-6’-O-rhamnosyl-quercetin, HRESIMS spectrum, m/z 741.1880 [M-H]^-^

Figure 112: 3-O-glycosyl-2’-O-xylosyl-6’-O-rhamnosyl-quercetin, UV spectrum from HPLC-DAD

Figure 113: 3-O-glycosyl-2’-O-xylosyl-6’-O-rhamnosyl-quercetin, structure with chemical shifts (MeOH-d_3_), structure with chemical shifts (MeOH-d_3_), the acquired analytical data is in accordance with previous literature (Lou et al. 2001)

Figure 114: 3-O-glycosyl-2’-O-xylosyl-6’-O-rhamnosyl-quercetin, ­^1^H-NMR spectrum (MeOH-d_3_)

Figure 115: 3-O-glycosyl-2’-O-xylosyl-6’-O-rhamnosyl-quercetin, HSQC spectrum (MeOH-d_3_)

Figure 116: 3-O-glycosyl-2’-O-xylosyl-6’-O-rhamnosyl-quercetin, HMBC spectrum (MeOH-d_3_)

Figure 117: 3-O-glycosyl-2’-O-xylosyl-6’-O-rhamnosyl-quercetin, ^1^H-^1^H COSY spectrum (MeOH-d_3_)

Figure 118: 3-O-glycosyl-2’-O-xylosyl-6’-O-rhamnosyl-quercetin, HSQC-TOCSY spectrum (MeOH-d_3_)

Figure 119: 3-O-glycosyl-2’-O-xylosyl-6’-O-rhamnosyl-quercetin - selective TOCSY spectrum (MeOH-d_3_, o1p = 5.40 ppm)

Figure 120: 3-O-glycosyl-2’-O-xylosyl-6’-O-rhamnosyl-quercetin - selective TOCSY spectrum (MeOH-d_3_, o1p = 4.80 ppm)

Figure 121: 3-O-glycosyl-2’-O-xylosyl-6’-O-rhamnosyl-quercetin - selective TOCSY spectrum (MeOH-d_3_, o1p = 6.89 ppm)

Figure 122: 3-O-glycosyl-2’-O-xylosyl-6’-O-rhamnosyl-quercetin - selective TOCSY spectrum (MeOH-d_3_, o1p = 1.11 ppm)

## 2.11 Quercetin-3-*O*-sambubioside

Figure 123: Quercetin-3-O-sambubioside, HRESIMS spectrum, m/z 595.1337 [M-H+FA]^-^

Figure 124: Quercetin-3-O-sambubioside, UV spectrum from HPLC-DAD

Figure 125: Quercetin-3-O-sambubioside, structure with chemical shifts (MeOH-d_3_), the acquired analytical data is in accordance with previous literature (Li et al. 2014)

Figure 126: Quercetin-3-O-sambubioside, ­^1^H-NMR spectrum (MeOH-d_3_)

Figure 127: Quercetin-3-O-sambubioside, HSQC spectrum (MeOH-d_3_)

Figure 128: Quercetin-3-O-sambubioside, HMBC spectrum (MeOH-d_3_)

Figure 129: Quercetin-3-O-sambubioside, HSQC-TOCSY spectrum (MeOH-d_3_)

Figure 130: Quercetin-3-O-sambubioside, ^1^H-^1^H COSY spectrum (MeOH-d_3_)

Figure 131: Quercetin-3-O-sambubioside – selective TOCSY spectrum (MeOH-d_3_, o1p = 5.49 ppm)

Figure 132: Quercetin-3-O-sambubioside - selective TOCSY spectrum (MeOH-d_3_, o1p = 4.77 ppm)

## 2.12 Rutin

Figure 133: Rutin, HRESIMS spectrum, m/z 609.1478 [M-H]^-^

Figure 134: Rutin, UV spectrum from HPLC-DAD

Figure 135: Rutin, structure with chemical shifts (MeOH-d_3_), the acquired analytical data is in accordance with previous literature (Han et al. 2004)

Figure 136: Rutin, ­^1^H-NMR spectrum (MeOH-d_3_)

Figure 137: Rutin, ­DEPT spectrum (MeOH-d_3_)

Figure 138: Rutin, HSQC spectrum (MeOH-d_3_)

## 2.13 Quercetin-3-*O*-glucoside

Figure 139: Quercetin-3-O-glucoside, HRESIMS spectrum, m/z 463.0906 [M-H]^-^

Figure 140: Quercetin-3-O-glucoside, UV spectrum from HPLC-DAD

Figure 141: Quercetin-3-O-glucoside, structure with chemical shifts (MeOH-d_3_) , the acquired analytical data is in accordance with previous literature (Han et al. 2004)

Figure 142: Quercetin-3-O-glucoside, ­^1^H-NMR spectrum (MeOH-d_3_)

Figure 143: Quercetin-3-O-glucoside, HSQC spectrum (MeOH-d_3_)

## 2.14 Quercetin-3-*O*-glucuronide

Figure 144: Quercetin-3-O-glucuronide, HRESIMS spectrum, m/z 477.0789 [M-H]^-^

Figure 145: Quercetin-3-O-glucuronide, UV spectrum from HPLC-DAD

Figure 146: Quercetin-3-O-glucuronide, structure with chemical shifts (MeOH-d_3_), the acquired analytical data is in accordance with previous literature (Kim et al. 2015)

Figure 147: Quercetin-3-O-glucuronide, ­^1^H-NMR spectrum (MeOH-d_3_)

Figure 148: Quercetin-3-O-glucuronide, HSQC spectrum (MeOH-d_3_)

Figure 149: Quercetin-3-O-glucuronide, HMBC spectrum (MeOH-d_3_)

Figure 150: Quercetin-3-O-glucuronide, ^1^H-^1^H COSY spectrum (MeOH-d_3_)

## 2.15 Kaempferol-3-*O*-glucuronide

Figure 151: Kaempferol-3-O-glucuronide, HRESIMS spectrum, m/z 461.0751 [M-H]^-^

Figure 152: Kaempferol-3-O-glucuronide, UV spectrum from HPLC-DAD

Figure 153: Kaempferol-3-O-glucuronide, structure with chemical shifts (DMSO-d_6_), the acquired analytical data is in accordance with previous literature (Parvez et al. 2021)

Figure 154: Kaempferol-3-O-glucuronide, ­^1^H-NMR spectrum (DMSO-d_6_)

Figure 155: Kaempferol-3-O-glucuronide, HSQC spectrum (DMSO-d­_6_)

Figure 156: Kaempferol-3-O-glucuronide, HMBC spectrum (DMSO-d_6_)

Figure 157: Kaempferol-3-O-glucuronide, ^1^H-^1^H COSY spectrum (DMSO-d_6_)

# Synthesis of reference compounds

## 3.1 Caffeic acid-4-*O*-glucoside

Caffeic acid-4-*O*-glucoside was synthesized according to Galland et Al. (Galland et al. 2007).

Figure 158: Caffeic acid-4-O-glucoside, HRESIMS spectrum, m/z 341.0878 [M-H]^-^

Figure 159: Caffeic acid-4-O-glucoside, structure with chemical shifts (MeCN-d_3_)

Figure 160: Caffeic acid-4-O-glucoside, ­^1^H-NMR spectrum (MeCN-d_3_)

Figure 161: Caffeic acid-4-O-glucoside, ­^1^H-^1^H COSY (MeCN-d_3_)

Figure 162: Caffeic acid-4-O-glucoside, HSQC spectrum (MeCN-d_3_)

Figure 163: Caffeic acid-4-O-glucoside, HMBC spectrum (MeCN-d_3_)

# Reference compounds

## 4.1 3-*O*-Salicyloyl quinic acid

Figure 164: 3-O-salicyloyl quinic acid, HRESIMS spectrum, m/z 311.0789 [M-H]^-^

Figure 165: 3-O-salicyloyl quinic acid, UV spectrum from HPLC-DAD

## 4.2 4-*O*-Salicyloyl quinic acid

Figure 166: 4-O-salicyloyl quinic acid, HRESIMS spectrum, m/z 311.0787 [M-H]^-^

Figure 167: 4-O-salicyloyl quinic acid, UV spectrum from HPLC-DAD

## 4.3 5-O-Salicyloyl-quinic acid

Figure 168: 5-O-salicyloyl quinic acid, HRESIMS spectrum, m/z 311.0797 [M-H]^-^

Figure 169: 5-O-salicyloyl quinic acid, UV spectrum from HPLC-DAD

## 4.4 4-*O*-Benzoyl quinic acid

Figure 170: 4-O-benzoyl quinic acid, HRESIMS spectrum, m/z 295.0846 [M-H]^-^

Figure 171: 4-O-benzoyl quinic acid, UV spectrum from HPLC-DAD

## 4.5 5-*O*-Benzoyl quinic acid

Figure 172: 5-O-benzoyl quinic acid, HRESIMS spectrum, m/z 295.0847 [M-H]^-^

Figure 173: 5-O-benzoyl quinic acid, UV spectrum from HPLC-DAD

## 4.6 3,4-*O*-bissalicyloyl quinic acid

Figure 174: 3,4-O-bissalicyloyl quinic acid, HRESIMS spectrum, m/z 431.1017 [M-H]^-^

Figure 175: 3,4-O-bissalicyloyl quinic acid, UV spectrum from HPLC-DAD

## 4.7 3,5-*O*-bissalicyloyl quinic acid

Figure 176: 3,5-O-bissalicyloyl quinic acid, HRESIMS spectrum, m/z 431.1009 [M-H]^-^

Figure 177: 3,5-O-bissalicyloyl quinic acid, UV spectrum from HPLC-DAD

## 4.8 4,5-*O*-bissalicyloyl quinic acid

Figure 178: 4,5-O-bissalicyloyl quinic acid, HRESIMS spectrum, m/z 431.1020 [M-H]^-^

Figure 179: 4,5-O-bissalicyloyl quinic acid, UV spectrum from HPLC-DAD

## 4.9 3-*O*-salicyloyl-4-*O*-benzoyl quinic acid

Figure 180: 3-O-salicyloyl-4-O-benzoyl quinic acid, HRESIMS spectrum, m/z 415.1069 [M-H]^-^

Figure 181: 3-O-salicyloyl-4-O-benzoyl quinic acid, UV spectrum from HPLC-DAD

## 4.10 3-*O*-salicyloyl-5-*O*-benzoyl quinic acid

Figure 182: 3-O-salicyloyl-5-O-benzoyl quinic acid, HRESIMS spectrum, m/z 415.1060 [M-H]^-^

Figure 183: 3-O-salicyloyl-5-O-benzoyl quinic acid, UV spectrum from HPLC-DAD

## 4.11 4-*O*-salicyloyl-5-*O*-benzoyl quinic acid

Figure 184: 4-O-salicyloyl-5-O-benzoyl quinic acid, HRESIMS spectrum, m/z 415.1068 [M-H]^-^

Figure 185: 3-O-salicyloyl-5-O-benzoyl quinic acid, UV spectrum from HPLC-DAD

## 4.12 Tremuloidin

Figure 186: Tremuloidin, HRESIMS spectrum, m/z 435.1324 [M-H+FA]^-^

Figure 187: Tremuloidin, UV spectrum from HPLC-DAD

## 4.13 *ortho*-Hydroxyhippuric acid

Figure 188: o-Hydroxyhippuric acid, HRESIMS spectrum, m/z 194.0459 [M-H]^-^

Figure 189: o-Hydroxyhippuric acid, UV spectrum from HPLC-DAD

## 4.14 Salicin

Figure 190: Salicin, HRESIMS spectrum, m/z 285.0980 [M-H]^-^

Figure 191: Salicin, UV spectrum from HPLC-DAD

## 4.15 Salicylic acid

Figure 192: Salicylic acid, HRESIMS spectrum, m/z 137.0244 [M-H]^-^

Figure 193: Salicylic acid, UV spectrum from HPLC-DAD

# ^13^C-Labelling of frass metabolites from feeding experiments

## 5.1 [U-^13^C]Caffeoylquinic acid experiments

Figure 194: P.nigra – quinic acid, labelling pattern is shown from the main isotope peak. CGA, chlorogenic acid

Figure 195: P.nigra – protocatechuic acid, labelling pattern is shown from the main isotope peak

Figure 196: P.nigra – protocatechuic acid-glucoside (exemplarily drawn as 4-O-glucoside), labelling pattern is shown from the main isotope peak

Figure 197: P.nigra – caffeic acid, labelling pattern is shown from the main isotope peak

Figure 198: P.nigra – caffeic acid-4-O-glucoside, labelling pattern is shown from the main isotope peak

Figure 199: P.nigra – caffeic acid-4-O-glucoside, labelling pattern is shown from the main isotope peak

Figure 200: P.nigra – 3-O-benzoylquinic acid, labelling pattern is shown from the main isotope peak

Figure 201: P.nigra – 4-O-benzoylquinic acid, labelling pattern is shown from the main isotope peak

Figure 202: P.nigra – 5-O-benzoylquinic acid, labelling pattern is shown from the main isotope peak

Figure 203: P.nigra – 3-O-salicyloylquinic acid, labelling pattern is shown from the main isotope peak

Figure 204: P.nigra – 4-O-salicyloylquinic acid, labelling pattern is shown from the main isotope peak

Figure 205: P.nigra – 5-O-salicyloylquinic acid, labelling pattern is shown from the main isotope peak

Figure 206: P.nigra – 3,4-O-bisbenzoylquinic acid, labelling pattern is shown from the main isotope peak

Figure 207: P.nigra – 4,5-O-bisbenzoylquinic acid, labelling pattern is shown from the main isotope peak

Figure 208: P.nigra – 3-salicyloyl-4-O-benzoylquinic acid, labelling pattern is shown from the main isotope peak

Figure 209: P.nigra – 4-salicyloyl-5-O-benzoylquinic acid, labelling pattern is shown from the main isotope peak

Figure 210: P.nigra – 3,4-O-bissalicyloylquinic acid, labelling pattern is shown from the main isotope peak

Figure 211: P.nigra – 4,5-O-bissalicyloylquinic acid, labelling pattern is shown from the main isotope peak

## 5.2 [U-^13^C]Salicortinoid experiments

Figure 212: P.nigra – salicin, labelling pattern is shown from the main isotope peak

Figure 213: P.nigra – isosalicin, labelling pattern is shown from the main isotope peak

Figure 214: P.nigra – isosalicin – glucose moiety, labelling pattern is shown from the main isotope peak

Figure 215: P.nigra – catechol glucoside, labelling pattern is shown from the main isotope peak

Figure 216: P.nigra – 3-O-benzoyl-quinic acid, labelling pattern is shown from the main isotope peak

Figure 217: P.nigra – 4-O-benzoyl-quinic acid, labelling pattern is shown from the main isotope peak

Figure 218: P.nigra – 5-O-benzoyl-quinic acid, labelling pattern is shown from the main isotope peak

Figure 219: P.nigra – 3-O-salicyloyl-quinic acid, labelling pattern is shown from the main isotope peak

Figure 220: P.nigra – 4-O-salicyloyl-quinic acid, labelling pattern is shown from the main isotope peak

Figure 221: P.nigra – 5-O-salicyloyl-quinic acid, labelling pattern is shown from the main isotope peak

Figure 222: P.nigra – 3,4-O-bisbenzoyl-quinic acid, labelling pattern is shown from the main isotope peak

Figure 223: P.nigra – 3,5-O-bisbenzoyl-quinic acid, labelling pattern is shown from the main isotope peak

Figure 224: P.nigra – 4,5-O-bisbenzoyl-quinic acid, labelling pattern is shown from the main isotope peak

Figure 225: P.nigra – 3-O-salicyloyl-4-O-benzoyl-quinic acid, labelling pattern is shown from the main isotope peak

Figure 226: P.nigra – 4-O-salicyloyl-5-O-benzoyl-quinic acid, labelling pattern is shown from the main isotope peak

Figure 227: P.nigra – 3-O-salicyloyl-5-O-benzoyl-quinic acid, labelling pattern is shown from the main isotope peak

Figure 228: P.nigra – 3,4-O-bissalicyloyl-quinic acid, labelling pattern is shown from the main isotope peak

Figure 229: P.nigra – 3,5-O-bissalicyloyl-quinic acid, labelling pattern is shown from the main isotope peak

Figure 230: P.nigra – 4,5-O-bissalicyloyl-quinic acid, labelling pattern is shown from the main isotope peak

Figure 231: P.nigra – populin, labelling pattern is shown from the main isotope peak

Figure 232: P.nigra – salicylic acid, labelling pattern is shown from the main isotope peak

Figure 233: P.nigra – saligenin, labelling pattern is shown from the main isotope peak

Figure 234: P.nigra – hippuric acid, labelling pattern is shown from the main isotope peak

Figure 235: P.nigra – o-hydroxy-hippuric acid, labelling pattern is shown from the main isotope peak

Figure 236: P.nigra – tremulacinol, labelling pattern is shown from the main isotope peak

Figure 237: P.nigra – saligenin sulfate, labelling pattern is shown from the main isotope peak

Figure 238: P.nigra – catechol sulfate, labelling pattern is shown from the main isotope peak

Figure 239: P.nigra – o-hydroxyhippuric acid glucoside, labelling pattern is shown from the main isotope peak

Figure 240: P.nigra – DHCH glucoside, labelling pattern is shown from the main isotope peak

Figure 241: P.nigra – N-benzoylalanine, labelling pattern is shown from the main isotope peak

Figure 242: P.nigra – N-salicyloylalanine, labelling pattern is shown from the main isotope peak

Figure 243: P.nigra – nigracin, labelling pattern is shown from the main isotope peak

Figure 244: P.nigra – tremuloidin, labelling pattern is shown from the main isotope peak

Figure 245: P.nigra – 2’,6’-O-bisbenzoylsalicin, labelling pattern is shown from the main isotope peak

Figure 246: P.tremula x tremuloides – salicin, labelling pattern is shown from the main isotope peak

Figure 247: P.tremula x tremuloides – catechol glucoside, labelling pattern is shown from the main isotope peak

Figure 248: P.tremula x tremuloides – 3-O-benzoyl-quinic acid, labelling pattern is shown from the main isotope peak

Figure 249: P.tremula x tremuloides – 4-O-benzoyl-quinic acid, labelling pattern is shown from the main isotope peak

Figure 250: P.tremula x tremuloides – 5-O-benzoyl-quinic acid, labelling pattern is shown from the main isotope peak

Figure 251: P.tremula x tremuloides – 3-O-salicyloyl-quinic acid

Figure 252: P.tremula x tremuloides – 4-O-salicyloyl-quinic acid, labelling pattern is shown from the main isotope peak

Figure 253: P.tremula x tremuloides – 5-O-salicyloyl-quinic acid, labelling pattern is shown from the main isotope peak

Figure 254: P.tremula x tremuloides – 3,4-O-bisbenzoyl-quinic acid, labelling pattern is shown from the main isotope peak

Figure 255: P.tremula x tremuloides – 3,5-O-bisbenzoyl-quinic acid, labelling pattern is shown from the main isotope peak

Figure 256: P.tremula x tremuloides – 4,5-O-bisbenzoyl-quinic acid, labelling pattern is shown from the main isotope peak

Figure 257: P.tremula x tremuloides – 3-O-salicyloyl-4-O-benzoyl-quinic acid, labelling pattern is shown from the main isotope peak

Figure 258: P.tremula x tremuloides – 4-O-salicyloyl-5-O-benzoyl-quinic acid, labelling pattern is shown from the main isotope peak

Figure 259: P.tremula x tremuloides – 3-O-salicyloyl-5-O-benzoyl-quinic acid, labelling pattern is shown from the main isotope peak

Figure 260: P.tremula x tremuloides – 3,4-O-bissalicyloyl-quinic acid, labelling pattern is shown from the main isotope peak

Figure 261: P.tremula x tremuloides – 3,5-O-bissalicyloyl-quinic acid, labelling pattern is shown from the main isotope peak

Figure 262: P.tremula x tremuloides – 4,5-O-bissalicyloyl-quinic acid, labelling pattern is shown from the main isotope peak

Figure 263: P.tremula x tremuloides – hippuric acid, labelling pattern is shown from the main isotope peak

Figure 264: P.tremula x tremuloides – N-benzoylalanine, labelling pattern is shown from the main isotope peak

Figure 265: P.tremula x tremuloides – o-hydroxy-hippuric acid, labelling pattern is shown from the main isotope peak

Figure 266: P.tremula x tremuloides – N-salicyloylalanine, labelling pattern is shown from the main isotope peak

Figure 267: P.tremula x tremuloides – salicylic acid, labelling pattern is shown from the main isotope peak

Figure 268: P.tremula x tremuloides – tremuloidin, labelling pattern is shown from the main isotope peak

Figure 269: P.tremula x tremuloides – populin, labelling pattern is shown from the main isotope peak

Figure 270: P.tremula x tremuloides – 2’,6’-O-bisbenzoyl-salicin, labelling pattern is shown from the main isotope peak

Figure 271: P.tremula x tremuloides – DHCH, labelling pattern is shown from the main isotope peak

Figure 272: P.tremula x tremuloides – saligenin sulfate, labelling pattern is shown from the main isotope peak

Figure 273: P.tremula x tremuloides – catechol sulfate, labelling pattern is shown from the main isotope peak

Figure 274: P.tremula x tremuloides – o-hydroxyhippuric acid glucoside, labelling pattern is shown from the main isotope peak

## 5.3 [U-^13^C]Salicortinoid labelling pattern vs. [U-^13^C]caffeoylquinic acid labelling pattern

Figure 275: Comparison of 4-O-benzoylquinic acid labelling pattern

Figure 276: Comparison of 4-O-salicylquinic acid labelling pattern

Figure 277: Comparison of 3,4-O-bisbenzoylquinic acid labelling pattern

Figure 278: Comparison of 3,4-O-bissalicyloylquinic acid labelling pattern

Figure 279: Comparison of 3-O-salicyloyl-4-O-bisbenzoylquinic acid labelling pattern

## 5.4 [U-^13^C]Trichocarpin experiment

Figure 280: P.nigra – 3-O-benzoyl-quinic acid (**19**), labelling pattern is shown from the main isotope peak

Figure 281: P.nigra – 4-O-benzoyl-quinic acid, labelling pattern is shown from the main isotope peak

Figure 282: P.nigra – 5-O-benzoyl-quinic acid, labelling pattern is shown from the main isotope peak

Figure 283: P.nigra – 3,4-O-bisbenzoyl-quinic acid, labelling pattern is shown from the main isotope peak

Figure 284: P.nigra – 3,5-O-bisbenzoyl-quinic acid, labelling pattern is shown from the main isotope peak

Figure 285: P.nigra – 4,5-O-bisbenzoyl-quinic acid, labelling pattern is shown from the main isotope peak

Figure 286: P.nigra – 3-O-salicyloyl-4-O-benzoyl-quinic acid, labelling pattern is shown from the main isotope peak

Figure 287: P.nigra – 4-O-salicyloyl-5-O-benzoyl-quinic acid, labelling pattern is shown from the main isotope peak

Figure 288: P.nigra – 3-O-salicyloyl-5-O-benzoyl-quinic acid, labelling pattern is shown from the main isotope peak

Figure 289: P.nigra – isotrichocarpin, labelling pattern is shown from the main isotope peak

Figure 290: P.nigra – 6’-O-benzoyltrichocarpin, labelling pattern is shown from the main isotope peak

Figure 291: P.nigra – trichocarpin, labelling pattern is shown from the main isotope peak

Figure 292: P.nigra – gentisic acid glucoside, labelling pattern is shown from the main isotope peak

Figure 293: P.tremula x tremuloides – 3-O-benzoyl-quinic acid, labelling pattern is shown from the main isotope peak

Figure 294: P.tremula x tremuloides – 4-O-benzoyl-quinic acid, labelling pattern is shown from the main isotope peak

Figure 295: P.tremula x tremuloides – 5-O-benzoyl-quinic acid, labelling pattern is shown from the main isotope peak

Figure 296: P.tremula x tremuloides – 3,4-O-bisbenzoyl-quinic acid, labelling pattern is shown from the main isotope peak

Figure 297: P.tremula x tremuloides – 3,5-O-bisbenzoyl-quinic acid, labelling pattern is shown from the main isotope peak

Figure 298: P.tremula x tremuloides – 4,5-O-bisbenzoyl-quinic acid, labelling pattern is shown from the main isotope peak

Figure 299: P.tremula x tremuloides – 3-O-salicyloyl-4-O-benzoyl-quinic acid, labelling pattern is shown from the main isotope peak

Figure 300: P.tremula x tremuloides – 3-O-salicyloyl-4-O-benzoyl-quinic acid, labelling pattern is shown from the main isotope peak

Figure 301: P.tremula x tremuloides – 3-O-salicyloyl-5-O-benzoyl-quinic acid, labelling pattern is shown from the main isotope peak

Figure 302: P.tremula x tremuloides – isotrichocarpin, labelling pattern is shown from the main isotope peak

Figure 303: P.tremula x tremuloides – 6’-O-benzoyltrichocarpin, labelling pattern is shown from the main isotope peak

Figure 304: P.tremula x tremuloides – benzyl-gentisate, labelling pattern is shown from the main isotope peak

Figure 305: P.tremula x tremuloides –trichocarpin, labelling pattern is shown from the main isotope peak

# Metabolism of [U-^13^C]salicortinoids and [U-^13^C]trichocarpin

Figure 306: Metabolism of [U-^13^C]salicortin in *C. vinula*. Red parts of the structures indicate ^13^C-labelled parts of the molecules.

Figure 307: Metabolism of [U-^13^C]tremulacin in *C. vinula*. Red parts of the structures indicate ^13^C-labelled parts of the molecules.

Figure 308: Metabolism of [U-^13^C]trichocarpin in C. vinula. Red parts of the structures indicate ^13^C-labelled parts of the molecules.

# Stability of salicortinoids on *P. nigra* leaf surfaces

Figure 309: Stability test of salicortin. **A**) Detail of a ^1^H-NMR spectrum of 1 mg of salicortin isolated from Populus leaf material. The sample was taken from a refrigerated (4°C) stock and the spectrum was measured in MeOH-d_3_ and referenced to the residual solvent signal at δ_H_ 3.31. **B**) The same sample from A) was applied to the adaxial side of a P. nigra leaf and was left on the leaf for 16 hrs. Subsequently, the surface of the leaf was washed with MeOH. The solvent was evaporated with N_2_ gas and the residue was suspended in 1 mL of water. This solution was loaded to a conditioned and equilibrated 100 mg (85µm) HR-X SPE column, which was subsequently washed with water (2 mL) and dried in vacuum. The column was then eluted with 1.5 mL of MeOH, the eluate was evaporated to dryness with N_2_ gas and reconstituted with the NMR solvent (MeOH-d_3_). The spectra are identical with no signs of decomposition. Hence, salicortin (and other salicortinoids) are stable when applied to leaf surfaces for at least 16 hrs at room temperature.

# References

Choi YH et al. (2006) NMR Metabolomics to Revisit the Tobacco Mosaic Virus Infection in *Nicotiana tabacum* Leaves. Journal of Natural Products 69:742-748. doi:10.1021/np050535b

Dommisse RA, Van Hoof L, Vlietinck AJ (1986) Structural analysis of phenolic glucosides from salicaceae by NMR spectroscopy. Phytochemistry 25:1201-1204. doi:<https://doi.org/10.1016/S0031-9422(00)81580-0>

Fedorova DD, Nazarova DS, Avetyan DL, Shatskiy A, Belyanin ML, Kärkäs MD, Stepanova EV (2020) Divergent Synthesis of Natural Benzyl Salicylate and Benzyl Gentisate Glucosides. Journal of Natural Products 83:3173-3180. doi:10.1021/acs.jnatprod.0c00838

Galland S, Mora N, Abert-Vian M, Rakotomanomana N, Dangles O (2007) Chemical Synthesis of Hydroxycinnamic Acid Glucosides and Evaluation of Their Ability To Stabilize Natural Colors via Anthocyanin Copigmentation. Journal of Agricultural and Food Chemistry 55:7573-7579. doi:10.1021/jf071205v

Han J-T, Bang M-H, Chun O-K, Kim D-O, Lee C-Y, Baek N-I (2004) Flavonol glycosides from the aerial parts of*Aceriphyllum rossii* and their antioxidant activities. Archives of Pharmacal Research 27:390-395. doi:10.1007/BF02980079

Itoh A et al. (2000) Five Phenolic Glycosides from *Alangium Chinense*. Journal of Natural Products 63:95-98. doi:10.1021/np990391z

Iwai K, Kishimoto N, Kakino Y, Mochida K, Fujita T (2004) In Vitro Antioxidative Effects and Tyrosinase Inhibitory Activities of Seven Hydroxycinnamoyl Derivatives in Green Coffee Beans. Journal of Agricultural and Food Chemistry 52:4893-4898. doi:10.1021/jf040048m

Kanho H, Yaoya S, Kawahara N, Nakane T, Takase Y, Masuda K, Kuroyanagi M (2005) Biotransformation of Benzaldehyde-Type and Acetophenone-Type Derivatives by <i>*Pharbitis nil*</i> Hairy Roots. Chemical and Pharmaceutical Bulletin 53:361-365. doi:10.1248/cpb.53.361

Kim SY, Lee HR, Park K-s, Kim B-G, Ahn J-H (2015) Metabolic engineering of *Escherichia coli* for the biosynthesis of flavonoid-*O*-glucuronides and flavonoid-*O*-galactoside. Applied Microbiology and Biotechnology 99:2233-2242. doi:10.1007/s00253-014-6282-6

Li X, Yang L, Liu S, Fei D, Zhang M, Zhang Y (2014) Effect of Quercetin-3-*O*-Sambubioside Isolated from *Eucommia ulmoides* Male Flowers on Spontaneous Activity and Convulsion Rate in Mice. Planta Med 80:974-977. doi:10.1055/s-0034-1382902

Lou H, Yuan H, Yamazaki Y, Sasaki T, Oka S (2001) Alkaloids and Flavonoids from Peanut Skins. Planta Med 67:345-349. doi:10.1055/s-2001-14319

Parvez MK, Ahmed S, Al-Dosari MS, Abdelwahid MAS, Arbab AH, Al-Rehaily AJ, Al-Oqail MM (2021) Novel Anti-Hepatitis B Virus Activity of *Euphorbia schimperi* and Its Quercetin and Kaempferol Derivatives. ACS Omega 6:29100-29110. doi:10.1021/acsomega.1c04320

Sashidhara KV, Singh SP, Varshney S, Beg M, Gaikwad AN (2014) Poliothrysoside and its derivatives as novel insulin sensitizers potentially driving AMPK activation and inhibiting adipogenesis. European Journal of Medicinal Chemistry 86:570-577. doi:<https://doi.org/10.1016/j.ejmech.2014.09.015>

Toyama-Kato Y, Kondo T, Yoshida K (2007) Synthesis of designed acylquinic acid derivatives involved in blue color development of hydrangea and their co-pigmentation effect. Heterocycles 72:239-254. doi:10.3987/com-06-s(k)6

Zhu X, Dong X, Wang Y, Ju P, Luo S (2005) Phenolic Compounds from *Viburnum cylindricum*. Helvetica Chimica Acta 88:339-342. doi:<https://doi.org/10.1002/hlca.200590017>
